# Species-resolved sequencing of low-biomass or degraded microbiomes using 2bRAD-M

**DOI:** 10.1186/s13059-021-02576-9

**Published:** 2022-01-26

**Authors:** Zheng Sun, Shi Huang, Pengfei Zhu, Lam Tzehau, Helen Zhao, Jia Lv, Rongchao Zhang, Lisha Zhou, Qianya Niu, Xiuping Wang, Meng Zhang, Gongchao Jing, Zhenmin Bao, Jiquan Liu, Shi Wang, Jian Xu

**Affiliations:** 1 Single-Cell Center, CAS Key Laboratory of Biofuels and Shandong Key Laboratory of Energy Genetics, Qingdao Institute of BioEnergy and Bioprocess Technology, Chinese Academy of Sciences, Qingdao, China; 2 Channing Division of Network Medicine, Brigham and Women’s Hospital and Harvard Medical School, Boston, USA; 3 University of Chinese Academy of Sciences, Beijing, China; 4 UCSD Health Department of Pediatrics and Center for Microbiome Innovation at Jacobs School of Engineering, University of California, San Diego, San Diego, USA; 5 Faculty of Dentistry, The University of Hong Kong, Hong Kong SAR, China; 6 Procter & Gamble Singapore Innovation Center, Singapore, Singapore; 7 Sars-Fang Centre & MOE Key Laboratory of Marine Genetics and Breeding, Ocean University of China, Qingdao, Qingdao, China; 8 Qingdao OE Biotechnology Company Limited, Qingdao, China; 9 Key Laboratory of Dairy Biotechnology and Engineering, Ministry of Education, Inner Mongolia Agricultural University, Hohhot, China; 10 Qingdao National Laboratory of Marine Science and Technology, Qingdao, China; 11 Laboratory of Tropical Marine Germplasm Resources and Breeding Engineering, Sanya Oceanographic Institution, Ocean University of China, Sanya, China

## Abstract

Microbiome samples with low microbial biomass or severe DNA degradation remain challenging for amplicon-based or whole-metagenome sequencing approaches. Here, we introduce 2bRAD-M, a highly reduced and cost-effective strategy which only sequences ~ 1% of metagenome and can simultaneously produce species-level bacterial, archaeal, and fungal profiles. 2bRAD-M can accurately generate species-level taxonomic profiles for otherwise hard-to-sequence samples with merely 1 pg of total DNA, high host DNA contamination, or severely fragmented DNA from degraded samples. Tests of 2bRAD-M on various stool, skin, environmental, and clinical FFPE samples suggest a successful reconstruction of comprehensive, high-resolution microbial profiles.

**Supplementary Information:**

The online version contains supplementary material available at 10.1186/s13059-021-02576-9.

## Introduction

Metagenome sequencing, widely used to derive the taxonomic profile of microbiome, typically adopts two strategies that target (i) amplicons of phylogenetic “marker genes” (e.g., 16S rRNA for bacteria and archaea, and 18S rRNA or internal transcribed spacer (ITS) for fungi) or (ii) the whole genomes (whole metagenome shotgun; WMS). Although less costly, marker gene analyses can be limited in taxonomic resolution (i.e., at the genus level) and susceptible to PCR bias in composition and abundance estimates [1]; moreover, they are usually unable to capture a landscape-like view that includes bacteria, archaea, fungi, and virus due to the lack of universal primers. In contrast, by sequencing the total DNA, WMS can resolve species- or strain-level taxonomy and offers a landscape-like view that includes all domains of organisms [2, 3]. However, although 1 ng is applicable via certain specialized kits [4], WMS usually requires a high amount of DNA as the starting material (≥50 ng preferred, 20 ng at a minimum) and moreover is not efficient in tackling DNA samples that are low in biomass, heavily degraded, or dominated by host DNA [1, 4]. Furthermore, WMS is typically much more costly, due to the much higher sequencing volume required for covering the whole genome (instead of just the marker gene). Therefore, new methods should be developed that cost-efficiently produce accurate, species-resolution, landscape-like taxonomic profiles for challenging samples like low-biomass, high host-contaminated, and degraded microbiomes.

Restriction site-associated DNA sequencing (RADseq) that utilizes restriction enzymes to digest genomic DNA from a broad range of organisms and sequences only the digested fragments has been applied to genotype variable genetic markers as well as for microbial species detection [5, 6]. Although RADseq has demonstrated the ability to produce species-resolution microbiome profiles with lower costs [7–10], the large size variation of DNA fragments after enzyme digestion results in a high bias in PCR-based amplification and thus the low fidelity of reconstructed taxonomic profile [11, 12]. Moreover, RADseq has not been thoroughly and systematically benchmarked against marker gene-based or WMS approaches [7–10]. Therefore, it remains unclear whether RADseq can provide accurate, landscape-like taxonomic profiles for low-biomass and severely degraded microbiome samples.

To address these challenges, here, we proposed the “2bRAD sequencing for Microbiome” (2bRAD-M) method that utilizes Type IIB restriction enzymes to produce exclusively iso-length DNA fragments of 25–33 bp (depending on the selection of enzymes) for sequencing [13–15]. This approach reduces the bias in PCR-based fragment amplification and thus ensures the high fidelity of the taxonomic profile. This offers significant benefits especially for low-biomass, heavily contaminated, or degraded microbial DNA which requires more PCR cycles. Tests on simulated datasets, mock, and actual microbiome samples illustrate that 2bRAD-M, by sequencing just about 1% of genomes, accurately generates species-level taxonomic profiles for challenging samples: (i) of merely 1 pg total DNA, (ii) of 99% host DNA contamination, or (iii) consisting of highly degraded fragments just 50-bp long. For real stool, skin, and environment-surface samples, it accurately reconstructs a comprehensive, species-resolution profile of bacteria, archaea, and fungi. Furthermore, microbiome in the formalin-fixed paraffin-embedded (FFPE) tissue samples which were otherwise recalcitrant to sequencing can now be analyzed, and a species-resolved classifier discriminated the healthy tissue, pre-invasive cancer, and invasive cancer with 91.1% accuracy. The ability to profile low-biomass microbiomes at the species level is pivotal to expanding the boundary of the known microbial world.

## Results

### The principle and workflow of 2bRAD-M

**Fig. 1:**
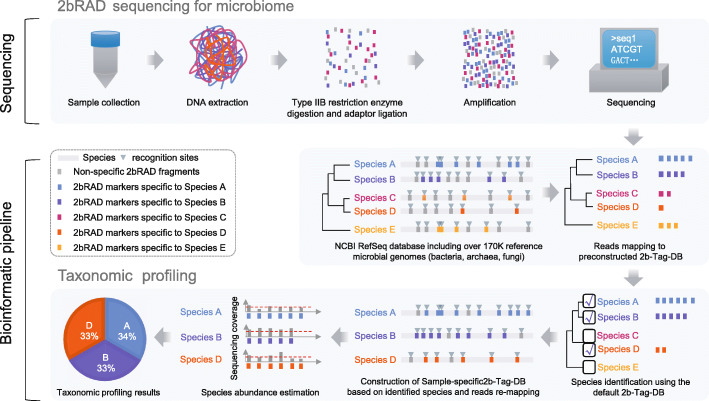
Scheme of the 2bRAD-M workflow. In the library preparation module of the 2bRAD-M pipeline, DNA samples were first digested using a type IIB restriction enzyme. The resulting 2bRAD fragments were enriched and amplified for DNA sequencing. In the computational module of the 2bRAD-M pipeline, we employed both prebuilt and sample-specific 2bRAD marker database to perform taxonomic profiling on 2bRAD data. Firstly, all reads were mapped against the default prebuilt unique 2bRAD marker database (2b-Tag-DB) to identify all candidate species in a 2bRAD-M sample. Next, to accurately estimate the abundance of identified species, we increased the number of taxa-specific 2bRAD markers for each candidate species by reconstructing a reduced 2bRAD marker database (sample-specific 2b-Tag-DB) which contains more 2bRAD markers specific to each candidate species than those in the default 2b-Tag-DB. All the 2bRAD sequences were then remapped to this sample-specific 2b-Tag-DB for abundance estimation of candidate species. In principle, the relative abundance of a given species was calculated as the read coverage of all species-specific 2bRAD markers. For more information, please refer to the “Materials and Methods” section

The principle and appealing features of 2bRAD-M are as follows (Fig. 1): (i) reliable enzyme-digested sequence tags can be derived that are specific to high-resolution taxa (e.g., species or strain) yet universally applicable for a broad range or all of bacterial, archaeal, and fungal genomes; (ii) these taxa-specific, iso-length sequence tags can be evenly amplified and sequenced; and (iii) the tag sequences can be mapped to reference genomes to reconstruct faithfully the taxonomic composition.

Specifically, the experimental workflow has two steps: (i) BcgI (a commercially available Type IIB restriction enzyme) is used, as an example, to digest total genomic DNA extracted from microbiome samples. BcgI recognizes the sequence of CGA-N_6_-TGC in the genomic DNA and cleaves on both upstream (12–10 bp) and downstream (10–12 bp) of this signature [13], producing short and iso-length DNA (32 bp without sticky ends) across all loci [14, 15]. (ii) These so-called 2bRAD fragments are ligated to adaptors, amplified, and then sequenced.

In the computational workflow, the foundation is a unique 2bRAD tag database (“2b-Tag-DB”), which contains taxa-specific 2bRAD tags identified from all the sequenced bacteria, fungi, and archaea genomes. Mapping the 2bRAD reads against 2b-Tag-DB thus identifies the presence of species in a sample. Subsequently, to estimate the relative abundance of the identified taxa, the mean read coverage of all 2bRAD tags specific to each taxon is derived. To improve the utilization rate of reads and classification accuracy, a secondary, sample-specific 2b-Tag-DB was dynamically derived from only those candidate taxa identified in a particular sample, which produces more species-specific 2bRAD tags than the original 2b-Tag-DB and results in more accurate modeling of relative abundance of taxa.

### The feasibility of 2bRAD-M for microbiome analysis via in silico simulation

To identify the taxa-specific tags, we first downloaded 173,165 microbial genomes (171,927 bacteria, 293 fungi, and 945 archaea; representing 26,163 species) from NCBI RefSeq (Oct 2019) to create a 2b-Tag-DB via in silico restriction digestion of these genomes using BcgI restriction enzyme [13]. This yields an average of 3010 iso-length (32 bp) 2bRAD tags per genome, and a total of 114,132,487 BcgI-digested unique species-specific 2bRAD tags that are of single-copy within a genome (average 1194 per species genome; Additional file 1: Supplemental Methods). Besides BcgI, species-specific 2bRAD tags (unique 2bRAD tags) were also identified for each of the other 15 Type IIB enzymes (AlfI, AloI, BaeI, BplI, BsaXI, BslFI, Bsp24I, CjeI, CjePI, CspCI, FalI, HaeIV, Hin4I, PpiI, and PsrI; Additional file 1: Table S1, Fig. S1a) to establish the usability of all Type IIB enzymes for 2bRAD-M. Notably, 2bRAD tags are detected in all genomes regardless of the Type IIB restriction enzyme used (Additional file 1: Fig. S1b). Moreover, the number of 2bRAD tags within a genome and their GC content are highly consistent with the length and the GC% of genomes (*r* > 0.98) (Additional file 1: Fig. S2) for all the 16 Type IIB restriction enzymes, suggesting unbiased and broadly applicable representation of the microbial genomes by these tags.

**Fig. 2:**
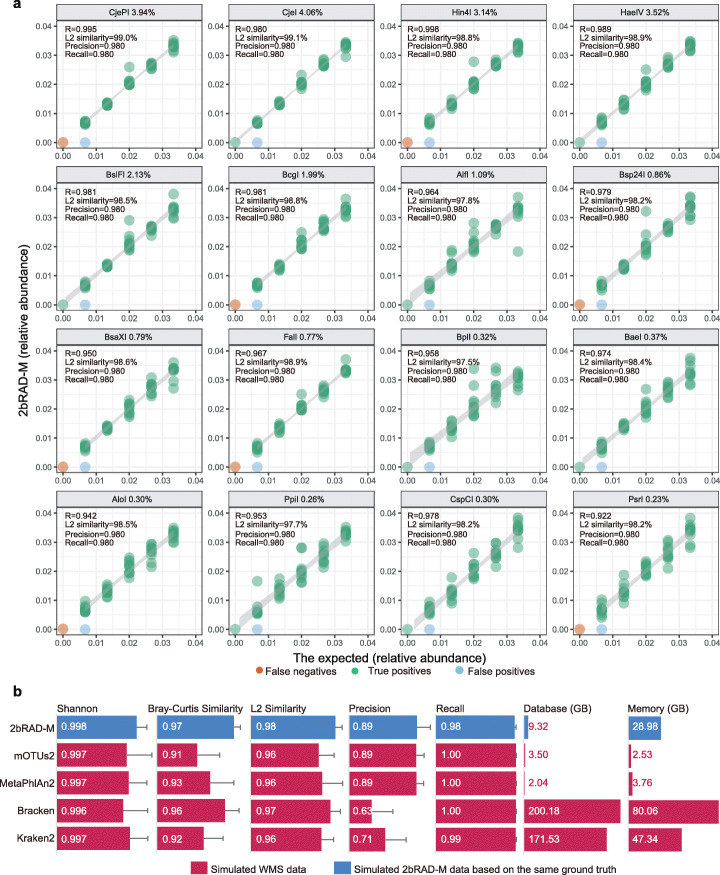
Benchmark measurements of 2bRAD-derived taxonomic profile. **a** Simulated microbial community data consisting of 50 microbes were profiled by each of the 16 type IIB restriction enzymes. The scatter plots indicate the correlation of the taxonomic abundance estimated from 2bRAD-M with the expected abundance for each enzyme. The percentage number indicated in each plot represents the average genome coverage (compare to the original 50 microbial genomes) after digesting by the enzymes. **b** Performance comparison of 2bRAD-M with Kraken2, Bracken, MetaPhlAn2, and mOTUs2 based on 25 simulated communities. Two types of abundance are used as the ground truth of the simulation data to evaluate the performance: sequence abundance is used to evaluate Bracken and Kraken2, while taxonomic abundance is used to evaluate 2bRAD-M, mOTUs2, and MetaPlAn2

To test whether these species-specific tags enable detection and abundance profiling of all known species in a community, a simulated 50-species microbiome was generated (one genome per species; randomly selected from RefSeq; Additional file 1: Table S2) and profiled using the default 2b-Tag-DB and sample-specific 2b-Tag-DB for each of the 16 Type IIB restriction enzymes. Performance of 2bRAD-M in species detection was assessed via precision and recall (with relative abundance threshold of 0.0001), while that of species abundance evaluated via L2 similarity score (a metric of similarity adapted from L2 distance [16]), by comparing to the ground truth (Fig. 2a). Precision, recall, and L2 similarity of the taxonomic profiling are all remarkably high (average for the 16 enzymes—precision = 98.0%, recall = 98.0%, L2 similarity = 96.9%), and this is achieved with an average genome coverage of 1.50% among the 50 selected genomes (Fig. 2a, Additional file 1: Fig. S3).

The accuracy and computational efficiency of 2bRAD-M were assessed by comparison with highly cited WMS profiling tools such as Kraken2 [17], Bracken [18], mOTUs2 [19], and MetaPhlAn2 [20], using the in silico simulated data of 25 microbial communities for benchmarking [21] (2bRAD-M applied BcgI-derived species-unique markers to profile the simulated data, while the others produced the abundance profiles from simulated pair-end 150-bp WMS reads; the “Materials and Methods” section). In terms of average Shannon similarity, Bray-Curtis similarity, L2 similarity (accuracy metrics evaluated based on the ground truth), precision, and recall, 2bRAD-M showed a level of 0.998, 0.97, 0.98, 0.89, and 0.98, respectively, which either outperformed or are equivalent to others (Fig. 2b). As for database storage and memory use, 2bRAD-M requires < 10-GB disk space to store the reference marker database and a relatively low RAM of 30 GB (equivalent to a desktop computer) as compared to Centrifuge, Kraken2, and Bracken (Fig. 2b). Thus, the 2bRAD-M bioinformatic pipeline can provide accurate profiling results with high computational efficiency.

### High reproducibility and sensitivity of 2bRAD-M under challenging conditions

To assess 2bRAD-M ability to handle low-biomass, highly degraded, or heavily contaminated samples, we first constructed a mock community (Mock-CAS) consisting of five prevalent oral or gut bacterial species in equal proportion. With this mock community stock, three series of samples were prepared: (i) “LoA” (low amount)—samples with total DNA amounts from 50 ng, 20 ng, 10 ng, 1 ng, 100 pg, 10 pg to only 1 pg; (ii) “HiD” (high degradation)—100-ng degraded DNA samples where the DNA mixture was randomly sheared by DNAse I into fragments of mostly 150 bp and 50 bp in length; and (iii) “HoC” (host contamination)—100-ng samples spiked with human DNA to simulate 90% or 99% contamination by host genome. Three technical replicates were included for each of the LoA (*n* = 7*3), HiD (*n* = 2*3), and HoC (*n* = 2*3) groups. Each of the 33 samples from the three series was then sequenced by 2bRAD-M. In addition, a sample of 100-ng DNA was then prepared from the mock community stock and sequenced by WMS to be referenced as the positive control (WMS of 50 ng or lower DNA failed).

**Fig. 3:**
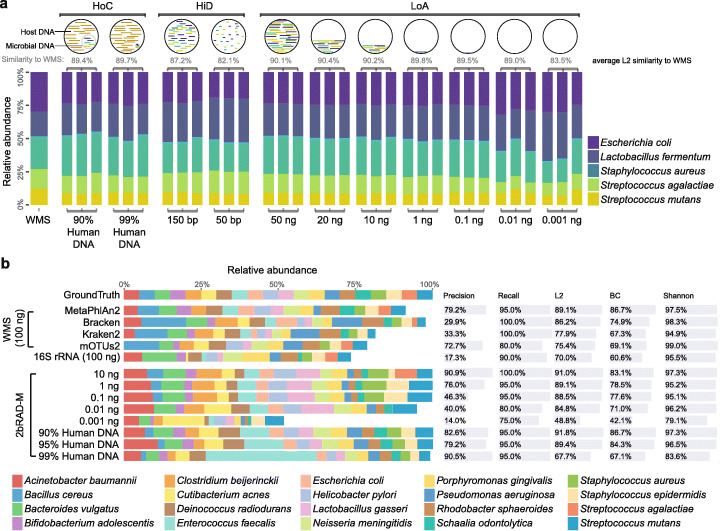
Comparison of taxonomic profiling results of 2bRAD-M, shotgun WGS, and 16S rRNA sequencing methods in mock microbial communities. **a** 2bRAD-M performance in samples with low amount (LoA), high DNA degradation (HiD), and high host DNA contamination (HoC) based on a mock community of five bacteria species (Mock-CAS). LoA are samples with a gradient of total DNA concentrations (50 ng, 20 ng, 10 ng, 1 ng, 0.1 ng, 0.01 ng, and 0.001 ng); HiD includes samples with fragmented bacterial DNA (50 bp or 150 bp). HoC are samples with a mixture of human DNA (90% or 99%) and bacterial DNA. Three technical replicates were included for each group. The L2 score that measures the microbial composition similarity between 2bRAD-M and WMS is shown on the head of each stacked bar plot. The false-positive identification rates of reads (i.e., reads mapped to species not in the mock community) are very low (0.9% in WMS and 1% in 2bRAD-M). **b** Benchmarking 2bRAD-M against conventional metagenomic approaches using the mock community of MSA 1002. Each stacked bar plot in the left panel shows the resulting taxonomic profile from a benchmarked method in a library preparation or bioinformatic setting. The white blank refers to false-positive identifications. For WMS data, we employed various bioinformatic tools (e.g., MetaPhlAn2, Bracken, Kraken2, and mOTUs2) to generate taxonomic profiles on MSA 1002. For 2bRAD-M data, we applied our own bioinformatic pipeline to generate the taxonomic profiles of MSA 1002 with low to high DNA amounts, and under various degrees of host DNA contamination. In the right panel, bars in each row indicate the corresponding precision, recall, L2 similarity, Bray-Curtis dissimilarity, and Shannon index similarity of the predicted taxonomic profile as compared to the ground truth. The bars in the “Shannon” column represent the correlation between the Shannon index of the profiling results and the ground truth (see the “Materials and Methods”)

To compare the results and minimize performance bias introduced by bioinformatic pipelines, we employed Centrifuge [22] to map both WMS (150-bp) and 2bRAD-M (32-bp) generated reads to the reference microbial genomes for taxonomic abundance estimation. For 2bRAD-M, tags from the actual data of each sample covered avg. 97.1% in silico predicted tags from the five genomes, indicating high consistency between observed and expected 2bRAD tags (which is key to accurate and reliable taxonomic profiling). Moreover, for each sample, the 2bRAD-M results are highly consistent among the three biological replicates (avg. L2 similarity: 95.4%; Fig. 3a).

Notably, for the LoA, HiD, and HoC groups, the average L2 similarity between 2bRAD-M profiles and the reference positive control is 88.9%, 84.6%, and 89.6%, respectively, with the global average being 88.2% (ranging from 82.1 to 90.4%; Fig. 3a). These observations are consistent across the technical replicates, indicating high reproducibility of 2bRAD-M. Specifically, in the LoA group, the L2 similarity of the 1-pg sample can still realize a respectable 83.5% as compared to 90.1% for the 50-ng sample (Fig. 3a). This suggests that 2bRAD-M offers high sensitivity and stable performance in low-biomass samples over a broad DNA-amount range (from 50 ng to 1 pg). In the HiD group, the L2 similarity is 87.2% and 82.1% for the 150-bp and 50-bp samples, respectively, indicating DNA degradation did not have a large negative effect and the 2b-RAD-M can effectively accommodate severe DNA degradation while providing reliable results. For the HoC group, the L2 similarity is 89.4% in the 90%-host-contaminated samples and 89.7% in 99%-host-contaminated samples (in addition, a certain degree of enrichment of microbiome-originated reads versus host-originated reads is apparent under HoC; Additional file 1: Table S3), suggesting 2bRAD-M’s ability to provide reliable microbial profiling in the presence of host DNA contamination.

To further probe the capability of 2bRAD-M in profiling a highly complex microbial community at low-biomass setting, we used ATCC MSA 1002, a standard, commercially available mock community consisting of 20 bacterial species (from 18 genera) with at equal DNA abundance among species [23]. Samples with total DNA amount ranging from 10 ng, 1 ng, 100 pg, 10 pg, and 1 pg and DNA host contamination from 90%, 95%, and 99% were prepared from this mock community for 2bRAD-M profiling. As a reference control, a 100-ng sample was profiled with the 16S-rRNA and WMS approaches (the 1-pg~10-ng samples failed). The precision for 2bRAD-M is 90.9%, 76.0%, 46.3%, 40.0%, 14.0%, 82.6%, 79.2%, and 90.5% for the 10 ng, 1 ng, 100 pg, 10 pg, 1 pg, 90% host DNA, 95% host DNA, and 99% host DNA samples, respectively. This is in contrast to the 53.7% (average number of different profilers) for WMS-100 ng and the 17.3% for 16S-rRNA-100 ng (Fig. 3b). Therefore, 2bRAD-M offers much lower false positives in detecting the species than 16S-rRNA and WMS. As for recall, 2bRAD-M is 100.0%, 95.0%, 95.0%, 80.0%, 75.0%, and 95% for 10 ng, 1 ng, 100 pg, 10 pg, 1 pg, and host DNA-contaminated samples, respectively, as compared to the 93.7% for WMS-100 ng and the 90.0% for 16S-rRNA-100 ng. Therefore, 2bRAD-M offers a comparable level of sensitivity to WMS and 16S-rRNA when starting with down to 100 pg of DNA. Moreover, the L2 similarity for 2bRAD-M is 91.0%, 89.1%, 88.5%, 84.8%, 48.5%, 91.8%, 89.4%, and 67.7% for 10 ng, 1 ng, 0.1 ng, 10 pg, 1 pg, 90% host DNA, 95% host DNA, and 99% host DNA samples, respectively, as compared to 82.1% for WMS-100 ng and 70.0% for 16S-rRNA-100 ng. As for alpha diversity (Shannon index) and beta diversity (Bray-Curtis similarity), profiling results of the low-biomass samples by 2bRAD-M are comparable to the corresponding high biomass samples by WMS and 16S as well (alpha diversity: averagely 92.5% by 2bRAD-M, 97.4% by WMS, and 95.5% by 16S; beta diversity: averagely 73.8% by 2bRAD-M, 74.6% by WMS, and 60.6% by 16S). Taken as a whole, these results demonstrated 2bRAD-M’s ability to profile complex microbiota with a high level of sensitivity and specificity.

### 2bRAD-M enables cost-effective deep microbiome profiling of real samples (fecal)

To assess the performance of 2bRAD-M on real samples, we performed and compared 2bRAD-M and 16S rRNA sequencing on human fecal samples (*n* = 3). In addition, each sample was also subjected to ultra-deep WMS sequencing (mean of 437 million reads or 239.73 Gb per sample) with the resultant taxonomic profiles used as evaluation reference [24].

**Fig. 4:**
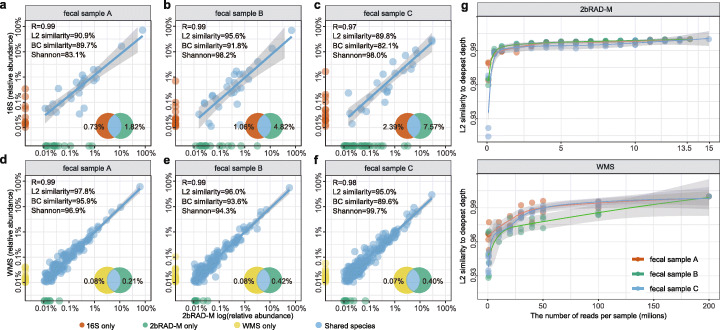
Comparison of taxonomic profiles and desired sequencing depth of 2bRAD-M, 16S, and WMS in fecal samples. **a**–**c** Comparison of taxonomic profiles at the genus level between 16S rRNA and 2bRAD-M. **d**–**f** Comparison of taxonomic profiles at the species level between 2bRAD-M and WMS. To perform the comparison in a fair manner, we extracted 2bRAD reads from WMS data and then used these WMS-originated 2bRAD reads as input for the 2bRAD-M computational pipeline. Then, species abundance generated by 2bRAD-M (using 2bRAD-M sequencing data) is used as the *X*-axis while the species abundance generated by 2bRAD-M (using WMS data) as the *Y*-axis for the scatter plot. **g** The rarefaction analysis of 2bRAD-M and WMS samples. The species-level compositions in the subsampled data at each given sequencing depth were compared to the pre-rarefaction result for each method

Taxonomic profiles from 2bRAD-M are compared with those from 16S rRNA sequencing (Fig. 4a–c) and WMS (Fig. 4d–f; Additional file 1: Supplemental Methods). Specifically, at the genus level, results of 2bRAD-M and 16S rRNA are highly consistent (mean Pearson correlation *R* = 0.997 and mean L2 similarity L2 = 92.0%), and avg. 95.27% of genus identified by 16S were also detected by 2bRAD-M (Additional file 1: Table S4). As for the species level, to perform the comparison in a fair manner, we extracted 2bRAD reads from WMS data and then used these WMS-originated 2bRAD reads as input for the 2bRAD-M computational pipeline; then, we found that WMS-originated 2bRAD reads and real 2bRAD sequencing data are also concordant in species-level profiling results, as evidenced by a high Pearson correlation (*R* = 0.99) and high L2 similarity (up to 97.8%). Moreover, only 0.40% of the taxa in WMS were not identified in 2bRAD-M (Additional file 1: Table S5). Thus, 2bRAD-M can produce highly complete and accurate species-level profiles that are equivalent to WMS.

One determinant of quality (and cost) of taxonomic profiling is sequencing depth [25]. To test how it influences 2bRAD-M performance, the fecal sequencing datasets were subsampled to various depths for 2bRAD-M (average 13.5 million reads per sample, subsampled at 0.1, 1, 2, 3, 5 to 13.5 million sequences per sample) or WMS (subsampled at 0.5, 10, 20, 30, 40, 50, 100, and 200 million sequences per sample since one 2b read can be identified and fetched from about 15 WMS reads) respectively. Then, we asked how many 2bRAD sequences are required to accurately quantify key ecological metrics such as alpha diversity (Shannon index), beta diversity (Bray-Curtis), and species compositions (species richness). In each of the fecal samples, with 20–40 million reads per sample, WMS identified averagely 170–182 species, but the Shannon diversity and species abundance estimates are far from saturation (i.e., reaching the values derived by sequencing ~ 400 million reads). In contrast, with just 3–4 million reads per sample, 2bRAD-M identified 173–188 species in each fecal samples and yield Shannon diversity and species abundance estimates comparable with those from the deepest (~ 13.5 million) read depth (Additional file 1: Fig. S4).

To evaluate the optimal sequencing depth of 2bRAD-M for species-level taxonomic profiling, we evaluated the similarity of taxonomic profiles derived at each reduced sequencing depth to its original taxonomic profile using the L2 similarity score. For 2bRAD-M, a sequencing depth of over 0.5 million reads per sample can achieve a L2 similarity score of 98.9% (1 million for 99.1%), which however requires many more (at least about 50 million) reads per sample in WMS (Fig. 4g). Overall, with 2~3 million reads (i.e., 60 Mb of sequencing data) per sample, 2bRAD-M can generate consistent, accurate, and stable alpha diversity estimates and taxonomic profile at the species level.

### 2bRAD-M enables species-resolved analysis of low-biomass skin, home, and car samples

To assess 2bRAD-M performance on actual low-biomass samples, we collected samples from the human skin surface (underarm, *n* = 20; Additional file 1: Table S6) for 16S-rRNA and 2bRAD-M analysis (WMS was aborted due to too-low DNA amounts for library construction). To gauge the potential impact of contaminating DNA (introduced from regents, in workflow, etc.) in the low-biomass samples [26], MSA 1002 was included as a control. MSA 1002 profiling revealed the expected microbial community profile while unforeseen microbes were absent which indicates minimal contamination. Between 16S rRNA and 2bRAD-M, a high degree of consistency in taxonomic profiles at the genus level was observed, with avg. L2 similarity of 81.1% (Table 1; Additional file 1: Fig. S5). In both methods, *Staphylococcus* (2bRAD-M: avg. 47.17%; 16S rRNA: avg. 44.43%) and *Corynebacterium* (2bRAD-M: avg. 10.38%; 16S rRNA: avg. 15.06%) are recognized as dominant microbes, consistent with existing literature [27–29].

**Table 1:** High concordance of dominant microbial taxa in 16S rRNA and 2bRAD-M profiling results of 20 underarm samples. The left three columns illustrate the top five genera in 16S profiling results of the 20 underarm samples with average relative abundance and ranking. The right three columns are the corresponding species’ average relative abundance and ranks identified by 2bRAD-M

Top genus in 16S	Relative abundance	Rank	Corresponding species in 2bRAD-M	Relative abundance	Rank
*Staphylococcus*	44.43%	1	*Staphylococcus epidermidis*	27.56%	1
			*Staphylococcus hominis*	12.57%	2
			*Staphylococcus capitis*	4.78%	5
			*Staphylococcus haemolyticus*	1.78%	11
			The other 4 species	0.48%	
			SUM	47.17%	
*Corynebacterium*	15.06%	2	*Corynebacterium ureicelerivorans*	2.58%	8
			*Corynebacterium jeddahense*	2.41%	9
			*Corynebacterium aurimucosum*	1.80%	10
			*Corynebacterium pseudogenitalium*	1.29%	15
			*Corynebacterium kefirresidentii*	1.23%	17
			*Corynebacterium tuberculostearicum*	1.08%	19
			The other 47 species	2.08%	
			SUM	12.47%	
*Uruburuella*	5.67%	3	*Uruburuella suis*	5.80%	4
*Enterococcus*	4.49%	4	*Enterococcus devriesei*	1.68%	12
			*Enterococcus dispar*	1.48%	13
			The other 3 species	0.45%	
			SUM	3.61%	
*Moraxella*	02.34%	5	*Moraxella osloensis*	2.77%	7
			The other 1 species	0.04%	
			SUM	2.81%	
SUM	**72.00%**		**SUM**	**71.86%**	

We further explored the application of 2bRAD-M, by testing on indoor environmental low-biomass samples, which are of significant health implications due to their contact with humans [30]. Surfaces of floor mat (*n* = 5) and cushion (*n* = 3) in the car and interior surfaces of a house (*n* = 4) were sampled and subjected to both 16S rRNA and 2bRAD-M (similarly, WMS was aborted due to the low DNA amounts; Additional file 1: Table S6). High L2 similarity (average 85.6% among samples; Additional file 1: Fig. S5) between 2bRAD-M and 16S rRNA was observed, suggesting stable performance of 2bRAD-M for such indoor samples. At the species level (Additional file 2), we identified 4929, 2335, and 1626 taxa for the three niches, respectively, whose dominant bacterial species are highly distinct: (i) floor mats are dominated by *Kocuria rosea* (23.23%), *Psychrobacter 1501_2011* (11.44%), and *Acinetobacter johnsonii* (10.43%); (ii) cushions are dominated by *Cutibacterium acnes* (42.19%), *Lactobacillus delbrueckii* (10.62%), and *Ralstonia pickettii* (10.14%); (iii) home surfaces are mainly colonized by *Streptococcus mitis* (49.64%), *Prevotella copri* (39.14%), and *Megamonas funiformis* (18.36%). Rarefaction of sequence depth (e.g., sequenced 2b-tags) via alpha diversity, beta diversity, and species-level compositions support high robustness of 2bRAD-M for such low-biomass indoor samples (Additional file 1: Fig. S6).

**Fig. 5:**
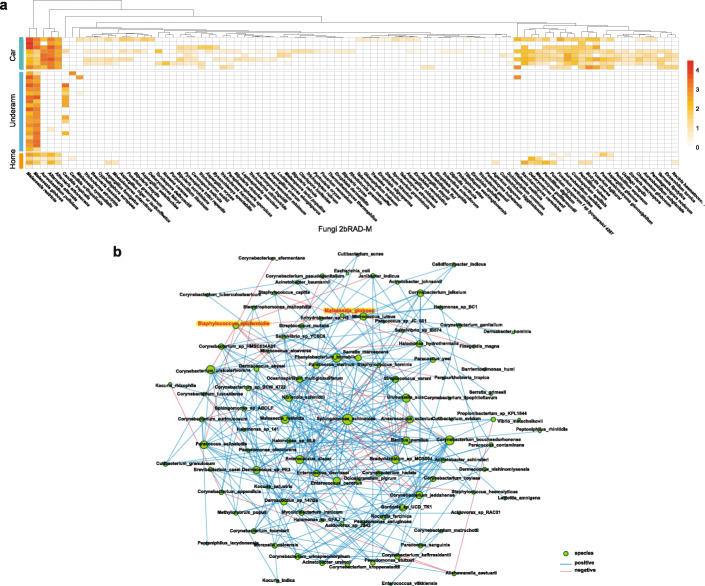
2bRAD-M analysis of low-biomass samples collected from underarm, home, and car surfaces. **a** Heat map shows identified fungal species via 2bRAD-M in the underarm, home, and car samples. **b** Co-occurrence network of bacterial and fungal species based on 20 underarm samples profiled by 2bRAD-M. Each green circle represents a species, and its size refers to the degree centrality score. Blue/red edges indicate positive/negative Spearman coefficients

Similar to WMS, 2bRAD-M enabled species-level profiles for fungi along with bacteria. In general, the relative abundance of fungi in the underarm and indoor environment samples is extremely low (0.83%) as compared to bacteria (99.16%) (Additional file 1: Table S7). Possible reasons for this observation include (i) micro-environments of these samples are not friendly fungal growth [31] and (ii) the relatively small number of available fungi genomes curated in our reference database limits the discovery of the fungi population. Nonetheless, among all the sites, the samples taken from the car cushion are found to harbor the highest amount of fungi (2.33%) with the surfaces from home being the lowest (0.06%). In addition, the 2bRAD-M species-level profile unveils distinctive patterns of fungal composition among the various sites (Fig. 5a). *Malassezia restricta* and *Malassezia globose*, known commensals on human skin surface [32], were found as the top abundance fungi at underarm and most of the indoor environmental samples. It was possible that the *Malassesia* sp. was transferred from humans, given the likely high contact frequency between the skin and these indoor environmental surfaces. Conversely, *Alternaria alteranta* was mostly found in indoor environments but absent in the underarm which is in agreement with existing reports [33, 34]. Thus, the fungi profiles derived from 2bRAD-M are consistent with literature, demonstrating the ability to reliably profile fungi (simultaneously with bacteria) in low-biomass samples.

Taking advantage of 2bRAD-M’s capability in profiling bacterial and fungal species simultaneously, an occurrence-network analysis revealed a negative correlation between the human skin commensal yeast of *Malassezia globosa* (which is associated with Seborrheic Dermatitis [35]) and the aforementioned *S. epidermidis* (recently proposed as a gatekeeper of healthy skin [36]; Spearman coefficient of −0.569; Fig. 5b). Such landscape-like, species-level correlations for low-biomass microbiomes can potentially reveal novel bacteria-fungi interactions.

### 2bRAD-M enables tumor microbiome profiling from FFPE tissue samples

Microbiota in human tumor or blood tissues were recently associated with the types, developmental stages, or chemotherapeutic efficiency of cancer [37–41]. Formalin-fixed, paraffin-embedded (FFPE) tissue, the gold standard of preserving tumor biopsy specimens [42, 43], represents a vast, irreplaceable historical clinical resource of enormous value for cancer microbiome studies [44]; however, profiling microbiome from FFPE tissues has been challenging due to the low microbial biomass, high human DNA contamination, severe DNA damage, and cross-links by chemical modification [45]. To test whether 2bRAD-M can tackle FFPE samples, we started with three pairs of healthy lung tissues from lung adenocarcinoma patients (typical of low microbial biomass), each derived both before (i.e., fresh tissue) and after FFPE (the “Materials and Methods” section). For each of the three pairs of lung tissue samples, a high consistency of microbial profiles derived using 2bRAD-M was reported between pre- and post-FFPE samples (Additional file 1: Fig. S7). These results thus support the ability of 2bRAD-M to accurately reconstruct microbiome structure from FFPE samples.

Next, we collected FFPE cervical tissue samples from 15 healthy controls (H), 15 pre-invasive cancerous (PreC; benign), and 15 invasive cancerous patients (InvaC; malignant) and subjected these samples to 2bRAD-M sequencing. DNA from the FFPE tissue was extracted from an area of 3cm^2^ with 4-μm thickness. On average, 25 ng/μl of DNA in each sample was detected as smears of size under 500 bp in agarose gels (Additional file 1: Fig. S8, Table S3), suggesting extremely low concentration and highly fragmented nature of DNA (both human and microbes [45]) in FFPE samples.

**Fig. 6:**
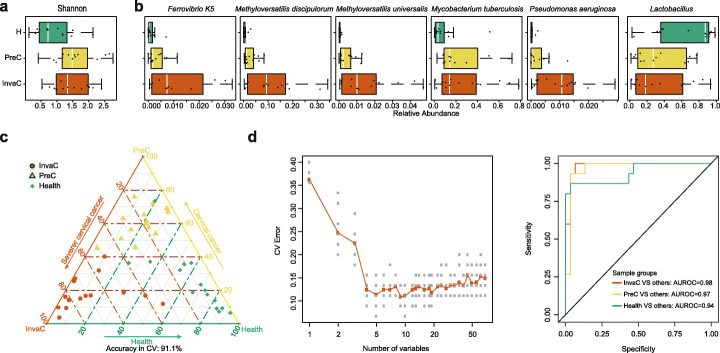
Microbiome-based diagnosis of FFPE thin section from cervical cancer samples as enabled by 2bRAD-M. **a** Shannon and Simpson index among 15 healthy controls (H), 15 pre-invasive cancerous (PreC; benign), and 15 invasive cancerous (InvaC; malignant) samples. **b** Comparison of differential species and *Lactobacillus* spp. among the three groups. **c** The Random Forest classifier for discriminating cancer and healthy samples. In the ternary plot, each dot represents a FFPE sample. The axes indicate the microbiome-based probability of being InvaC, PreC, and H for a FFPE sample. The closer one sample is to an apex, the more likely it is predicted as to be corresponding disease states. **d** Feature selection by rebuilding Random Forest classifiers using a series of reduced sets of features. The scatter plot shows that nine variables (species) in a reduced RF model (i.e., the AUC plot on the right) can maximize model performance. And the ROC curve shows an even better discriminate performance using binary categories (averagely 0.96)

The microbiome of the FFPE samples is mostly dominated by bacteria species (*n* = 243) with minimal fungal species (*n* = 2; Additional file 2). The alpha diversity (Shannon and Simpson index) in healthy controls (H) is significantly lower than PreC and InvaC (*p* = 0.044, Kruskal test; Fig. 6a). Among the identified bacterial species (Additional file 1: Fig. S9), samples in the PreC and InvaC groups are significantly enriched with *Methyloversatilis discipulorum* (*p*-value = 1.2e−5), *Mycobacterium tuberculosis* (*p*-value = 0.004), *Methyloversatilis universalis* (*p*-value = 1.8e−5), *Ferrovibrio K5* (*p*-value = 0.001), and *Pseudomonas aeruginosa* (*p*-value = 6.8e−5) (each increase in relative abundance from H to PreC and to InvaC; Fig. 6b). Conversely, *Lactobacillus* spp. (*L. paracasei*, *L. vaccinostercus*, *L. pentosus*, and *L. plantarum*) are greatly enriched in H (average abundance of 63.1%, in contrast to 33.9% and 32.9% in PreC and InvaC, respectively, Fig. 6b); this is consistent with a previous study [41] showing depletion of the *Lactobacillales* genera in “fresh,” non-FFPE cervical cancerous tissues. Notably, *Lactobacillus paracasei* shows anticancer potential against cervix cancer cells (HeLa) in vitro [46]. Furthermore, the enrichment of *Mycobacterium tuberculosis*, *Pseudomonas aeruginosa*, and *Staphylococcus aureus* in PreC- and InvC-phase PPFE samples is consistent with those “fresh” tissue-based studies that reported related genus-level (via 16S ^32^) or species-level (via WMS [41, 47]) taxa. Taken as a whole, 2bRAD-M has successfully captured the microbiome structure in FFPE samples and is able to reveal previously unknown discriminative microbial features between healthy and cancerous tissues. These features may serve as potential indicative novel markers for tumor onset and progression diagnosis. To evaluate this potential, we applied Random Forest on the FFPE taxonomic profiles at the species level, and the model distinguishes H, PreC, and InvaC samples with 91.1% accuracy (ten-fold cross-validation; Fig. 6c). Notably, we can achieve maximized discriminative performance (*AUC* 0.96) with the fewer features of the nine most important species in the RF model (Fig. 6d; Additional file 1: Table S8). Thus, 2bRAD-M offers a viable option for microbiome profiling on the vast archive of historical FFPE samples with potential application in early diagnosis and treatment of cancer.

## Discussion

Using mock microbiomes plus actual samples from the stool, skin, environment-surface, and frozen FFPE tissues, we demonstrated the ability of 2bRAD-M to profile microbiome samples that are challenging by using marker-gene or WMS sequencing approaches. Three key features are highlighted from this research. *Firstly*, it can analyze samples with low biomass (down to 1 pg), severe degradation, or high contamination. This advantage is built on the ability to (i) provide an unbiased (i.e., without the large size variation of DNA fragments) and reduced representation of metagenomes for sequencing, (ii) evenly cover all restriction sites, and (iii) generate short, iso-length reads and thus reliable quantification of tag abundance. With such advantage, 2bRAD-M is valuable to profiling microbiome samples from sparsely populated niches (e.g., indoor surfaces, blood, and skin), precious clinical specimen, or heavily degraded or contaminated tissues (e.g., FFPE sections and archeological samples). Notably, cervical cancer can be effectively cured in the preinvasive period [48], yet detection of this asymptomatic stage remains challenging which results in unnecessary delays in diagnosis and treatment [49]; therefore, 2bRAD-M sequencing of FFPE cervical cancer microbiomes, which is challenging for WMS or 16S rRNA approaches, promises to turn the enormous clinical repositories of FFPE tissue specimens into a treasure trove of microbiome-driven discoveries [42].

*Secondly*, it can provide species-level taxonomic information for bacteria, archaea, and fungi simultaneously. The multiple species-specific 2bRAD-M tags can uniquely identify the species from the microbial community, as resolution at the “species” level instead of the “genus” level can be crucial [50]. In this case, the ability to extract the species-level cancer-stage-specific markers from FFPE tissues, as well as to distinguish *S. epidermidis*, *S. hominis*, and *S. aureus* from underarm skin samples, is crucial to the diagnosis and mechanistic understanding of inflammation [29]. Interestingly, the overall ratio of fungal and bacterial abundance, a potential indicator of ecological balance [51], is 0.31% for underarm, 0.07% for home, and 1.27% for car (significantly different), suggesting characteristic ecological features among the sites.

*Finally*, using 2bRAD-M, the species-level taxonomic profiling can be achieved with a much lower sequencing cost than WMS. For example, only 1~5% of the sequencing data of WMS are required by 2bRAD-M to produce a taxonomic profile of equivalent accuracy, resulting in a cost reduction of 20–100 folds. Moreover, multi-isoRAD allows sequencing of five concatenated 2bRAD tags altogether via a single, 100–150-bp read from Illumina paired-end (PE) library [14], which further reduces the sequencing cost. The cost advantage is significant when high sequencing depth is required to address questions like unveiling the true microbial diversity or recovering rare but functionally important species in complex microbiota [25, 52]. Thus, 2bRAD-M appears especially suitable for such samples or circumstances. Notably, the performance of 2bRAD-M is limited by the availability and the potential bias of related reference genomes (e.g., 171927 bacterial and 945 archaeal genomes, yet only 293 fungal genomes in RefSeq), although the situation is rapidly improving with the steady increase of sequenced genomes for both cultured and uncultured microbes (e.g., via single-cell sequencing [53]).

## Conclusions

To overcome the bottlenecks of conventional metagenomic sequencing methods, we developed a new metagenomics method (2bRAD-M) that can cost-effectively produce accurate, species-resolution, landscape-like taxonomic profiles for challenging microbiome samples that are low-biomass, high-host-contaminated, and degraded. Tests on simulated datasets, mock microbiome, and actual microbiome samples showed that 2bRAD-M, by sequencing just about 1% of genomes, accurately generates species-level taxonomic profiles for samples that include merely 1 pg total DNA, are of 99% host DNA contamination, or consist of highly degraded fragments just 50 bp in length. Furthermore, it can accurately reconstruct a comprehensive, species-resolution profile of bacteria, archaea, and fungi for real stool, skin, environment-surface, and FFPE samples. Therefore, 2bRAD-M greatly expands the opportunities in microbiome research in challenging settings.

## Materials and methods

### Sample preparation and sequencing

#### Mock samples

Two mock microbial communities were used to validate the stability, sensitivity, and precision of 2bRAD-M. The first consists of five evenly mixed bacterial strains including *Streptococcus mutans* UA159, *Streptococcus agalactiae* ATCC13813, *Staphylococcus aureus* ATCC29213, *Escherichia coli* DH5α, and *Lactobacillus fermentum* ATCC9338. Then, three circumstances that simulated “challenging” microbiome samples were produced (with three replicates for each sample): (1) LoA: samples with low amount DNA; a concentration gradient from 10 ng to 1 pg was designed, with one-tenth of the concentration retained each time. (2) HiD: samples with highly degraded DNA; two samples with DNA lengths about 150 bp and 50 bp were included. (3) HoC: samples with host DNA contamination, which consist of either 90% or 99% human DNA. To avoid the bias introduced by human DNA removal, we did not perform any depletion methods before or after DNA extraction in this study.

The second mock used in this paper is the 20-Strain Even Mix Genomic Material (named as ATCC MOCK MSA 1002, 3.87 ng/μl; frozen 50 μl in Tris-HCl pH 8.5) which is purchased from ATCC. This mock comprises genomic DNA prepared from fully sequenced, characterized, and authenticated ATCC Genuine Cultures that were selected based on relevant phenotypic and genotypic attributes, such as Gram stain, GC content, genome size, and spore formation.

#### Fecal and FFPE tissue samples

The study protocol complies with the ethical guidelines of the 1975 Declaration of Helsinki, and institutional review board approval was obtained from Qingdao Institute of Bioenergy and Bioprocess Technology (QIBEBT), Chinese Academy of Sciences (CAS). Written informed consent and photography consent were obtained from each subject before enrollment. Three healthy adult individuals were enrolled as volunteers, and fecal samples were collected for deep WMS, 16S rRNA gene amplicon, and 2bRAD-M sequencing for comparison.

Three pairs of fresh and FFPE samples from healthy lung tissues of lung adenocarcinoma patients, and 45 cervical cancer-related sections in FFPE blocks (with a thickness of 4 μm and an area of 3 cm^2^), underwent microbiome profiling via 2bRAD-M. The 45 cervical cancer-related sections in FFPE blocks include 15 healthy controls (H), 15 pre-invasive cancerous (PreC; benign), and 15 invasive cancerous (InvaC; malignant) ones (determined by morphological evidence of polymorphonuclear infiltration). Each of the 45 samples was from a distinct individual, i.e., a cross-sectional design. To prepare the FFPE blocks, fresh tissue samples were fixed in phosphate-buffered formalin for 24–48 h, followed by tissue processing for 10 h, and paraffin embedding for 20 min [54]. Post-fixation processing of the tissues was completed in a histopathological laboratory using consistent processor protocols over years. Prior to microbiome profiling, the FFPE blocks were already stored at 17–22 °C and 20–60% humidity levels for 1–2 years. For these FFPE tissue sections, attempts to construct 16S amplicon or WMS libraries for microbiome sequencing both failed, due to the low quality of initial DNA (Additional file 1: Fig. S8).

#### Underarm, home, and car sampling collection protocol and processing

For the underarm sampling protocol, individuals were subjected to a washout period where the use of anti-bacterial products was not allowed for 4–5 days. After the washout period, a 22-mm D-squame tape strip (Cuderm) was applied onto the lower underarm skin surface (without hair) using a pressure applicator. The tape strips were then pre-treated with 200 mg of 0.1 mm Zirconia Silica beads and bead beat using Qiagen TissueLyser II (Valencia, CA) at 30 Hz for 3 min to lyse (via mechanical force) and dislodge the biomass from the tape strip. After dislodging the biomass from the tape strip, complete cell lysis is achieved via further enzymatic reaction by 2% w/v lysozyme, 0.05% w/v lysostaphin, and 1.2% Triton-X in TE buffer, followed by DNA extraction using Qiagen DNeasy Blood and Tissue Kit (Cat no. 69504, Valencia, CA). For the home and car sampling, the swab was used for wiping the surface of the cushion and carpet in the car and toy and toilet seat in the home. Specifically, wipe the swab for 20 times on an area of 4 × 4cm^2^. After collection, transfer the disposable swab to the tube containing sample storage liquid immediately and break the swab along the crease. Then, close the lid of the tube and make sure that there is no leakage. Finally, put the sample preservation solution tube in the biosafety bag for DNA extraction.

#### DNA extraction, 16S rRNA, and shotgun metagenomic sequencing

Genomic DNA was extracted from each fecal-containing tube using the Tissue and Blood DNA Isolation kit (Qiagen, Valencia, CA) following the manufacturer’s instructions with slight modifications. PCR amplification of the V1-V3 hypervariable regions of 16S rRNA genes was performed using the primer set (27F/534R) and followed the protocol developed by the Human Microbiome Project. PCR amplification reactions in triplicate for each sample were pooled at approximately equal amounts and sequenced, via the Illumina MiSeq 250 platform. All sequences were pre-processed following the standard QIIME (v.1.9) pipeline. Downstream bioinformatics analysis was performed using Parallel-Meta 3, a software package for comprehensive taxonomic and functional comparison of microbial communities. Clustering of OTUs was conducted at the 97% similarity level using a pre-clustered version of the Refseq database by BLASTN.

Paired-end metagenomic sequencing was performed for the two mock samples and fecal microbiota from three individuals via the Illumina HiSeq 2500 platform, yielding 239.73 ± 12.34 GB per sample (for fecal samples, with average fragment insert size of 350 bp and average read length of 150 bp). The reads were quality controlled by Trimmomatic (Sliding window 4:20; Minlength:100; MinPhred:25; Percentage of MinPhred: 80), and finally, 858,032,764 ± 13,140,670 clean reads per sample were generated and then profiled by mOTUs2 using default parameters.

#### 2bRAD-M sequencing

The 2bRAD-M library preparation basically followed the original protocol developed by Wang et al. [13] with minor modifications. Library preparation began with the digestion of 1 pg–200 ng genomic DNA in a 15-μl reaction using 4 U BcgI (NEB) at 37 °C for 3 h. Five microliters of the digested product was run on a 1% agarose gel to verify digestion. Next, ligation reaction was performed at 4 °C for 16 h in a 20-μl volume containing 10 μl of digested product, 0.2 μM each of library-specific adaptors (Ada1 and Ada2), 1 mM ATP (NEB), 1 × T4 DNA Ligase Buffer, and 800 U T4 DNA ligase (NEB). Then, heat inactivation was performed for BcgI at 65 °C for 20 min.

Ligation products were amplified in 40-μl PCRs, each composed of 7 μl ligated DNA, 0.1 μM each primer (Primer1 and Primer2 for Illumina), 0.3 mM dNTP, 1× Phusion HF buffer, and 0.4 U Phusion high-fidelity DNA polymerase (NEB). PCR was conducted in a DNA Engine Tetrad 2 thermal cycler (Bio-Rad) with 16–28 cycles of 98 °C for 5 s, 60 °C for 20 s, and 72 °C for 10 s and then a final extension of 10 min at 72 °C. The target band (Illumina ~ 100 bp) was excised from 8% (wt/vol) polyacrylamide gel, and the DNA diffused from the gel into nuclease-free water for 12 h at 4 °C. Finally, barcodes were introduced by means of PCR with platform-specific barcode-bearing primers. Forty-microliter PCR reaction contained 50 ng of gel-extracted PCR product, 0.2 μM of each primer (Primer1 and Primer3 for Illumina), 0.6 mM dNTP, 1× Phusion HF buffer, and 0.8 U Phusion high-fidelity DNA polymerase; seven cycles of the PCR profile listed above were performed. PCR products were purified by QIAquick PCR purification kit (Qiagen, Valencia, CA) and subjected to Illumina HiSeq platform sequencing. All primer and adaptor sequences are provided at Additional file 1: Table S9.

### Data analysis

#### Rationale of 2bRAD-M in overcoming high host contamination

We attribute the ability of 2bRAD-M to overcome high host contamination to the following. (i) By sequencing the representative 1% of the human genome and microbial genomes, 2bRAD-M can dramatically reduce the cost and hence greatly increase the sequencing depth. (ii) Due to the highly imbalanced presentation of enzyme sites in microbial genomes and the human genome, microbial genomes can generate many more 2bRAD tags than the human genome. For example, if we set the 1:99 as the microbial DNA vs human DNA ratio in the mock samples (MOCK CAS), the sequencing reads ratio would change to 3:97 in the actual sequencing results. (iii) The 2bRAD fragments in the human genome are completely different from those in microbial genomes (Additional file 1: Fig. S10). Consequently, qualitative analyses such as abundance estimation for microbial species are robust against host DNA interference, and the FPs would be greatly reduced.

#### Identification of species-specific 2bRAD-M markers from the most comprehensive genome database

Firstly, a total of 173,165 microbial genomes (including bacteria, fungi, and archaea) were downloaded from the NCBI RefSeq database. Then, built-in Perl scripts (GitHub: https://github.com/shihuang047/2bRAD-M) were used to sample restriction fragments from microbial genomes by each of 16 type 2B restriction enzymes, which formed a huge 2bRAD microbial genome database. The set of 2bRAD tags sampled from each genome was assigned under the GCF number, as well as GCF’s taxonomic information corresponding to the whole genome. Finally, all 2bRAD tags from each GCF that occur once within the genome were compared with those of all the others. Those 2bRAD tags are specific to a species-level taxon (having no overlap with other species) were developed as species-specific 2bRAD markers, collectively forming a 2bRAD marker database. The species-specific marker database has been shown to outperform other reference databases using all 2bRAD tags or full genomes in taxonomic profiling (Additional file 1: Fig. S11).

#### Simulation of 2bRAD-M sequencing data

To test the generalizability of our 2bRAD markers for microbial profiling, we first simulated 25 abundance profiles representing microbiota from distinct habitats (gut, oral, skin, vagina, and building, with five profiles for each) with known abundance profiles (abundance was created randomly from a log-normal distribution using the function *rlnorm* in the R language with the following parameters: meanlog = 0 and sdlog = 1). Given a specified species composition (taxonomic abundance), their sequence abundance can be inferred accordingly (taxonomic abundance equals sequence abundance divided by their genome length), and then Wgsim (https://github.com/lh3/wgsim) was used (with default parameters) to simulate the sequences. The intersection of organisms in the mOTU2, MetaPhlaAn2, and Kraken2 reference databases was used as the source genomes for the simulation. The simulation scripts for metagenomic data can be found in https://github.com/shihuang047/re-benchmarking.

#### Usage of metagenomic profilers

For shotgun metagenomic sequencing data, we validated the taxonomic profiles with state-of-the-arts profilers, such as Kraken2 [17], Bracken [18], mOTUs2 [19], MetaPhlAn2 [20], and Centrifuge [22]. The detailed procedures are listed below.

Kraken2 (v2.0.8-beta) is a *k-mer*-based taxonomic classification method. It searches for 35-bp *k-mers* from the query sequence in a precomputed database that matches k-mers to the lowest common ancestor (LCA) taxon of all genomes that contain that taxon. The default database was constructed using complete bacterial and viral genomes from NCBI RefSeq (2019 Oct). A filtering abundance threshold of 0.01 (default) was selected. The Kraken2 command below was used: “*kraken2 --threads 32 --fastq-input --gzip-compressed --paired input_1.fastq.gz input_2.fastq.gz –output output.reads --report output.report*”.

Bracken (v2.5) utilizes the read classification output from standard Kraken for a Bayesian re-estimation of taxonomic abundances, which significantly improves the false-positive issue of standard Kraken and implicitly normalizes for genome length. The kraken-filter was used to filter raw classifications at the 0.01 threshold. The Bracken command of “*est_abundance.py -i input -k db -o output*” was used.

MetaPhlAn2 (2.96.1) is a marker-gene alignment approach that relies on a precomputed database containing clade-specific marker genes. Query reads are aligned via bowtie2 to the marker genes for microbial identification and abundance estimation. The database version used is mpa v296 CHOCOPhlAn 201901. The MetaPhlAn2 command of “*metaphlan2.py input_1.fastq.gz, input_2.fastq.gz --input_type fastq --nproc threads --bowtie2out output_bowtie2out.txt -o output.report*” was used.

mOTUs2 (v2.5.1, database version 2.5.0) is a marker-based method that compiles a large variety of phylogenetic marker genes from multiple biomes. Query reads are aligned using bwa mem and further processed to generate an abundance profile. The mOTUs2 command of “*motus profile -f input_1.fastq.gz -r input_2.fastq.gz -o output.report -t threads*” was used.

Centrifuge (v1.0.3-beta) is a microbial classification engine based on FM-index which enables rapid, accurate, and sensitive taxonomic labeling of short reads. To analyze 2bRAD sequencing data of MOCK CAS (Fig. 3a), we employed Centrifuge to perform the 2bRAD-tag-level taxonomic classification that none of other existing profilers can handle. We started by customizing a database for Centrifuge that only contains genomes of the five species in MOCK CAS. Next, Centrifuge was used as a search engine to calculate the 2bRAD read-level proportion of five microbial species. Accordingly, we repeated this process using WMS for benchmarking purpose.

#### Calculation of relative abundance in 2bRAD-M

Firstly, to identify microbial species within each sample, all sequenced 2bRAD tags after quality control were mapped (using a built-in Perl script) against the 2bRAD marker database which contains all 2bRAD tags theoretically unique to each of 26,163 microbial species in RefSeq database (Fig. 1). To control the false-positive in the species identification, a *G* score was derived for each species identified within a sample as below, which is a geometric mean of the proportion of the species-specific markers that have been captured (by sequencing) and the number of all detected species-specific markers (by sequencing) of this species. The *G* score is more sensitive as the threshold than the relative abundance used in conventional WMS (Additional file 1: Fig. S12). The threshold of *G* score for a false-positive discovery of microbial species was set as 10.

$$ G\ {score}_{species\ i}=\sqrt{S_i\times {t}_i} $$

*S*: the number of reads assigned to all 2bRAD markers belonging to species *i* within a sample.

*t*: number of all 2bRAD markers of species *i* that have been sequenced within a sample

We estimated the relative abundance of each microbial species in a sample using the formula as below. We first calculated the average read coverage of all 2bRAD markers for each species, which represent the number of individuals belonging to a species present in a sample at a given sequencing depth. The relative abundance of a given species is then calculated as the ratio of the number of microbial individuals belonging to a species against the total number of individuals from known species that can be detected within a sample, with the default *G* score of 10.

$$ Relative\ {abundance}_{species\ i}=\frac{S_i/{T}_i}{\sum_{i=1}^n{S}_i/{T}_i} $$

*S*: the number of reads assigned to all 2bRAD markers of species *i* within a sample

*T*: the number of all theoretical 2bRAD markers of species *i*

#### Calculation of precision, recall, L2 similarity, and Pearson coefficient

For overall performance assessment, we applied precision and recall to evaluate the accuracy of microbial identification, while L2 distance [16] was employed to evaluate the accuracy of abundance estimation within a sample. Precision is the proportion of true positive species against the total number of species identified by a method, whereas recall is defined as the proportion of true positive species against the number of all species actually existing in a sample. To evaluate the accuracy of abundance profiles, we calculate the L2 distance between ground-truth abundance profile to each of the taxonomic profiles at a given taxonomic level (e.g., species or genus) produced by metagenomic sequencing methods. To visualize the benchmarking results more intuitively, we further employed L2 similarity calculated as the 1-L2 distance for performance comparisons. As for Shannon correlation, it is denoted by the difference between 1 and the absolute value of 1 minus the ratio of the predicted Shannon index and its ground truth. In addition, in Pearson correlation analysis, for those unique species that are identified only in one method, we replaced their abundance with 0 in calculating the Pearson correlation coefficient.

#### Diagnosis model of cancer samples

Random Forest models were trained to identify cancer status using the taxonomy profiles on the species level. Default parameters of the R implementation of algorithm were applied (R package ‘randomForest’, ntree=5000, using default mtry of *p*/3 where *p* is the number of input taxa). The performance of RF models based on microbiota was evaluated with a tenfold cross-validation approach.

Additional details are provided in Additional file 1: Supplemental Methods.

## Supplementary Information

**Additional file 1.** Supplemental Methods, Figures S1-S12, Tables S1-S9

**Additional file 2.** Additional data files

**Additional file 3.** Review history

## Data Availability

The sequencing data of 16S, WMS, and 2bRAD-M for MOCK (MOCK CAS and ATCC MOCK MSA 1002) and FFPE (only 2bRAD-M sequencing data) were submitted to figshare: 10.6084/m9.figshare.12272360.v8. The sequencing data of 16S and 2bRAD-M for typical low-biomass samples (underarm, home, and car) and fecal samples were submitted to 10.6084/m9.figshare.13514960.v4. All the above sequencing data were also submitted to SRA (SRP300672) under PRJNA689204 in NCBI [55] as well as the ultra-deep WMS data of stool samples. The 2bRAD-M computational pipeline and related database files are publicly available at Github [56]: https://github.com/shihuang047/2bRAD-M. The computational pipeline of 2bRAD-M is licensed under the MIT license. All source data and codes for the generation of figures and tables in the manuscript can be downloaded from: https://github.com/shihuang047/2bRAD-M-manuscript.
