# Supplementary material for: Species-resolved sequencing of low-biomass or degraded microbiomes using 2bRAD-M
Source: Genome Biol. 2022 Jan 26;23:36. doi: 10.1186/s13059-021-02576-9 (PMC8789378; doi:10.1186/s13059-021-02576-9)
Supplement: Supplementary file 1 — Additional file 1. Supplemental Methods, Figures S1-S12, Tables S1-S9 [file 13059_2021_2576_MOESM1_ESM.pdf]

Supplementary Materials for

Species-resolved sequencing of low-biomass microbiomes by 2bRAD-M

This file includes:

Supplemental Methods

Fig S1 to S12.

Tables S1-S9.

Supplemental Methods

Supplemental Figures

Fig S1. Distribution of unique 2bRAD tags and theoretically existent 2bRAD tags on different taxonomy levels.

Fig S2. The theoretical 2bRAD tags generated by 2bRAD-M and their originated genomes.

Fig S3. Comparison of the profiling performance between individual Type IIB restriction enzymes and a combined set of them.

Fig S4. Rarefaction analysis reveals the desirable sequencing depth for 2bRAD-M and WMS for reliable taxonomic profiling.

Fig S5. Comparison of the genus-level taxonomic profiles based on 16S rRNA sequencing and 2bRAD-M in each of the underarm, car or home samples.

Fig S6. Rarefaction analysis reveals the desirable sequencing depth of 2bRAD-M for taxonomic profiling of the representative built-environment and FFPE samples.

Fig S7. Comparing the taxonomic profiling results of 2bRAD-M between fresh (i.e., pre-FFPE) and post-FFPE lung tissues.

Fig S8. Agarose gel analysis of the DNA extracted from cervical FFPE tissue samples.

Fig S9. Species abundance profiles of the FFPE samples from healthy tissue, pre-invasive cancer and invasive cancer.

Fig S10. Very few 2bRAD fragments are shared across kingdoms.

Fig S11. Comparison of MSA 1002 profiling results using different databases.

Fig S12. G score provides a higher precision in taxonomic profiling than the relative abundance based on simulated sequencing data.

Supplemental Tables

Table S1. Availability of 2bRAD-M markers for taxonomic profiling at each of the taxonomic levels.

Table S2. Expected abundance of bacterial species in simulation data and profiling results by the 2bRAD-M computational pipeline.

Table S3. The relative enrichment of 2bRAD reads originated from microbial species versus those originated from host in the high-host-contamination (HoC) group.

Table S4. The relative abundance of major taxa identified in the three fecal samples at the species level using 2bRAD-M or WMS or at the genus level using 16S rRNA gene amplicon sequencing.

Table S5. The relative abundance of microbial species that are uniquely detected in the WMS or 2bRAD-M data of fecal samples.

Table S6. The initial DNA content and metadata for underarm skin, home and car samples.

Table S7. The 2bRAD-M profiling results for underarm, home and car samples.

Table S8. The relative abundance of bacteria, fungi and archaea in the indoor built-environmental samples.

Table S9. Species-level microbial organismal markers for the highly reliable diagnosis of cervical cancer from FFPE samples.

Table S10. The adaptors and primers used in 2bRAD-M sequencing (5'-3').

Supplementary Methods

Feasibility of 2bRAD-M for microbiome profiling by additional type IIB restriction enzymes

To test the feasibility of 2bRAD-M for microbiome profiling, two fundamental questions would be addressed in the *in silico* experiments based on an extensive set of microbial genomes: (*i*) whether the surveyed 2bRAD-M data is a reliable reduced representation of the microbial genomes; (*ii*) whether the surveyed 2bRAD-M data harbor phylogenetic markers that can enable the taxonomic profiling of microbial taxa at the species level.

We started by downloading 173,165 microbial genomes from NCBI RefSeq (Oct, 2019), including 15,162 bacterial, archaea and fungal complete genomes. The digital restriction digestion of all these genomes by an IIB restriction enzyme (such as BcgI) resulted in averagely 2930.38±2790.84 2bRAD-M tags per genome. To date, there are totally 16 type IIB restriction enzymes discovered, and they have distinct DNA recognition sites. We thus performed the digital digestion of all microbial genomes using all these 16 type IIB restriction enzymes, which produced multiple and flexible reduced representations of each microbial genome (**Fig. S1b**). Collectively, we identified and collected the restriction fragments of all microbial genomes using 16 restriction enzymes, which represent the most comprehensive 2bRAD-M reference genome database.

To assess whether the surveyed restriction fragments represent a random subset of a given microbial genome, we compared a number of features of the digitally digested DNA fragments from a given microbial genome to those from the entire genome. We found that the surveyed fragments are typically evenly distributed along a microbial genome, and across genic and non-genic regions. Likewise, %G + C content (53%) of surveyed 2bRAD-M tags are very similar to the genome-wide

averages (Pearson’s correlation R=0.992). Furthermore, the number of 2bRAD-M tags is highly correlated with the genome size of a given microbe (Pearson’s correlation R=0.976). This suggests that 2bRAD-M fragments can be employed to survey genome-wide features of microbes without requiring sequencing the full genome, regardless of the specific type-2B enzyme used here (**Fig. S2**).

We next sought to identify universal 2bRAD-M phylogenetic markers from a total set of 173,165 reference microbial genomes. Different strategies have been introduced to determine microbial community compositions and estimate their abundances from metagenomic data. Our approach is to identify taxa-specific DNA markers by analyzing the 2bRAD-M reference genome database and further quantify the read coverage of those markers for taxonomic profiling from 2bRAD-M data. Therefore, desired DNA markers in our study should be specific to taxa (i.e., species), iso-length (around 33 bp long) and short DNA fragments that only occur once per genome. Overall, the higher taxonomic level, the more 2bRAD-M tags are available (**Fig. S1a**). At the Kingdom level, almost all 2bRAD-M tags are kingdom-specific, thus there are very few shared 2bRAD-M tags among bacteria, fungi, archaea and human, regardless of the restriction enzymes. This suggested that the abundance ratio between the kingdoms can be readily derived from the 2bRAD-M data (yet can be challenging for WMS). The phylum-specific 2bRAD-M markers accounted for up to 90% ~ 97% of all theoretical 2bRAD-M tags produced from a given restriction enzyme from a given microbial genome. We next explored the 2bRAD-M markers specific to the 26,163 microbial species. Among all 521,289,189 restriction fragments produced by one typical type IIB restriction enzyme (BcgI), 99.21% are single-copy within a given microbial genome, while averagely 21.86% are specific to species-level taxa (**Table S1**). The other restriction enzymes can also generate distinct sets of 2bRAD-M tags from each

microbial genome. In fact, 18.81%-25.80% single-copy species-specific markers were identified from the 2bRAD-M genomes digested by the other restriction enzymes. Therefore, in principle, 2bRAD-M data provides a rich and highly flexible source of phylogenetic markers for metagenomic profiling.

Supplementary Figures

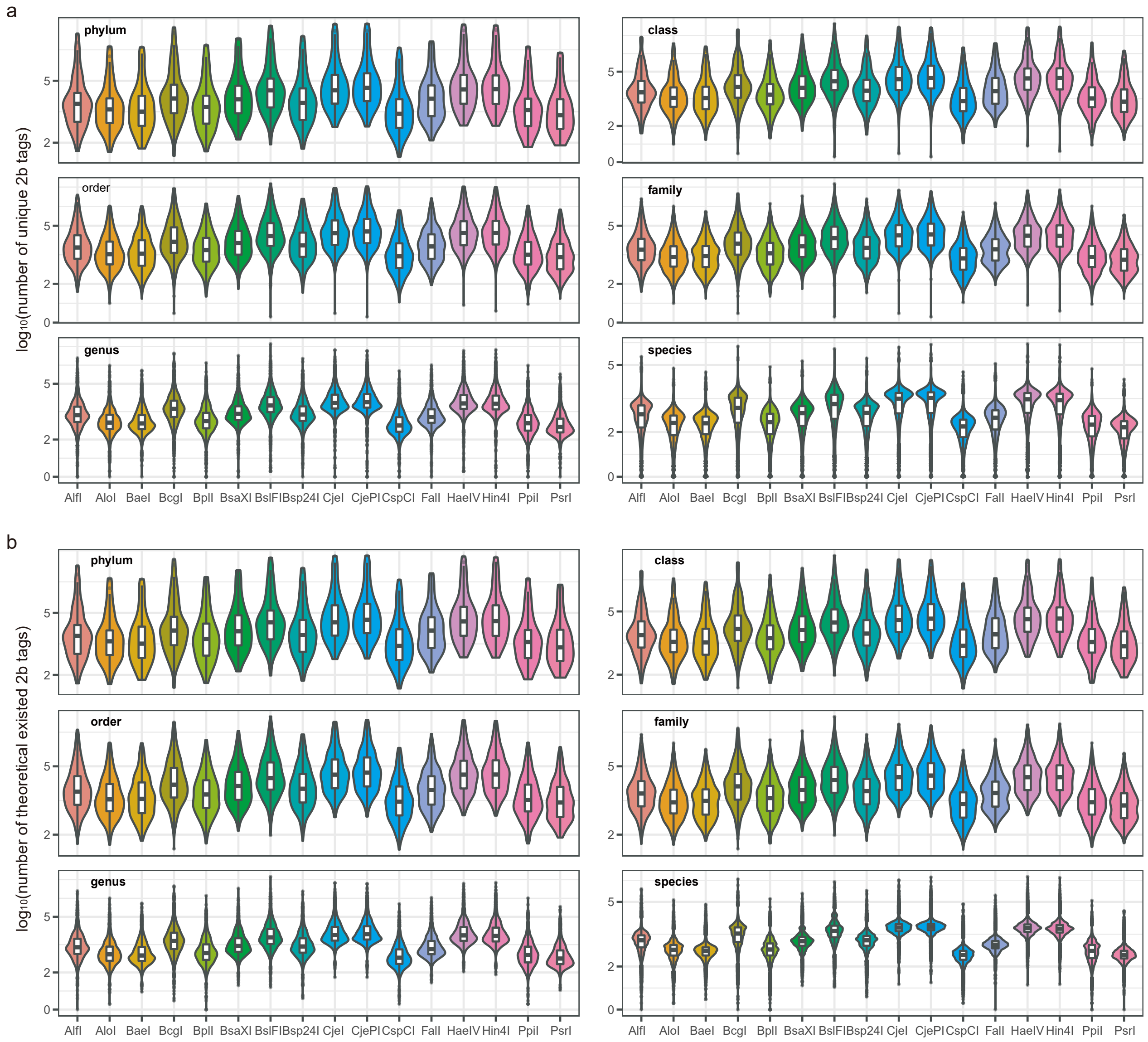

Fig S1. Distribution of unique 2bRAD tags and theoretically existent 2bRAD tags on different taxonomy levels. (**a**) Distribution of unique 2bRAD tags at various taxonomy levels. The 2bRAD tags were first generated by *in silico* digestion of 173,165 microbial genomes, and then non-redundantly merged based on their taxonomy annotation. We selected those 2bRAD tags that are not duplicated between any two taxa and named them as unique 2bRAD tags. The numbers of unique 2bRAD tags generated by different enzymes are shown at various taxonomy levels. The Type IIB restriction enzymes in X-axis are ordered by alphabet. (**b**) Distribution of theoretically existent 2bRAD tags on different taxonomy levels.

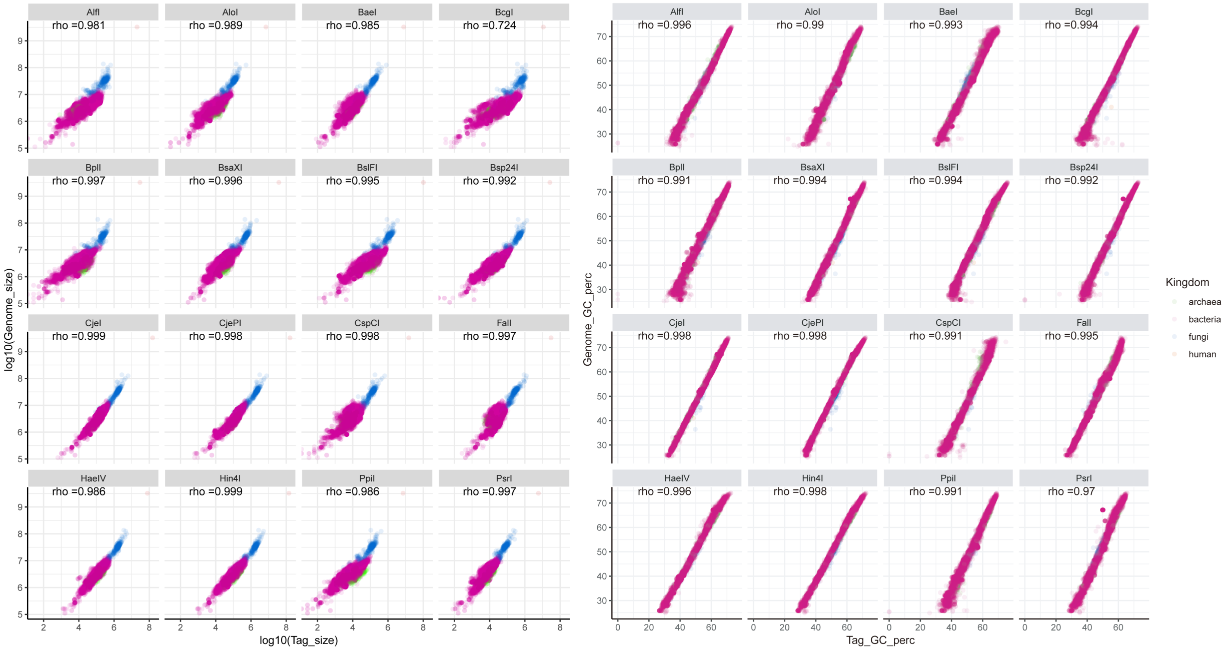

Fig S2. The theoretical 2bRAD tags generated by 2bRAD-M and their originated genomes. Correlation of fragment size (left panel) or GC content (right panel) is shown. For a given genome, the collective size of all DNA tags cleaved by a type IIB restriction enzyme corresponds to a reduction in sequencing for one to two orders of magnitude (depending on genome size).

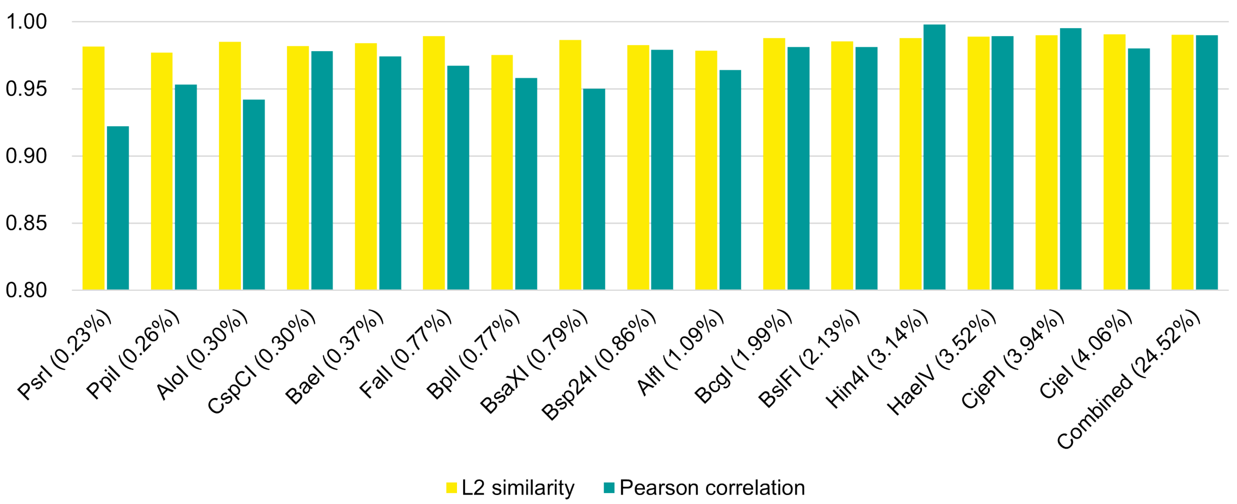

Fig S3. Comparison of the profiling performance between individual Type IIB restriction enzymes and a combined set of them. The bar plot shows the taxonomic profiling performance (L2 similarity and Pearson correlation) of the 2bRAD marker set from one of the 16 Type IIB restriction enzymes or a combined set. The X-axis shows Type IIB restriction enzymes and the percentage of original microbial genomic content that corresponding 2bRAD fragments can represent, whereas Y-axis shows the profiling performance. The yellow bars represent the L2 similarity between predicted and ground-truth abundances, and the green bars refers to the Pearson correlation between them. The combined marker set from 16 Type IIB restriction enzymes does not significantly improve performance in abundance estimation as compared to that from individual Type IIB restriction enzymes.

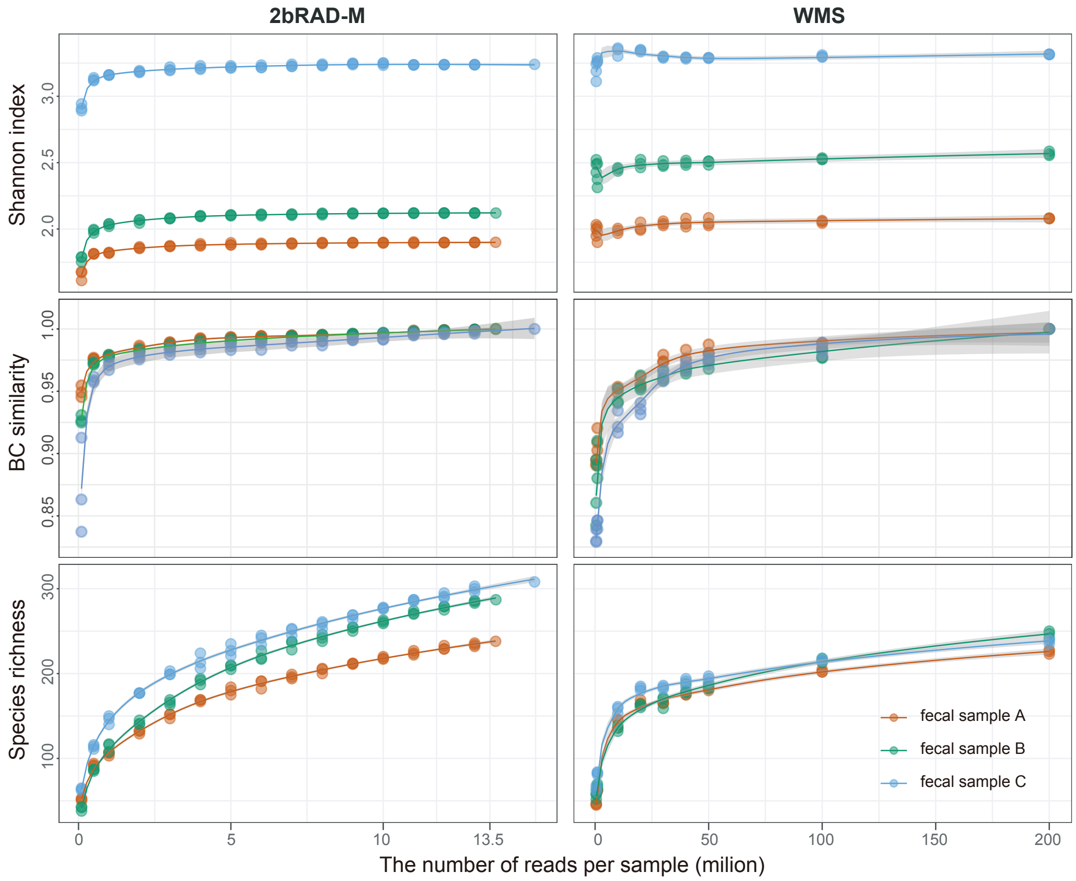

Fig S4. Rarefaction analysis reveals the desirable sequencing depth for 2bRAD-M and WMS for reliable taxonomic profiling. In each scatter plot, we compared the profiling results of a fecal sample based on a method (either 2bRAD-M or WMS) at deep or shallow (by subsampling) depth of sequencing, via Shannon diversity, beta diversity (on the Bray-Curtis similarity), and species richness. Based on the Shannon diversity and Bray-Curtis similarity, profiling performance of 2bRAD-M quickly saturates at a shallow sequencing depth (2-3 million reads per sample). In contrast, for the same metrics, WMS-based taxonomic profiles saturate at a far deeper sequencing depth (about 50 million reads per sample), suggesting much higher sequencing costs. The microbial richness (number of species-level taxa detected) based on both methods still grows as the sequencing depth increases, which is consistent with current knowledge on metagenomic diversity analysis.

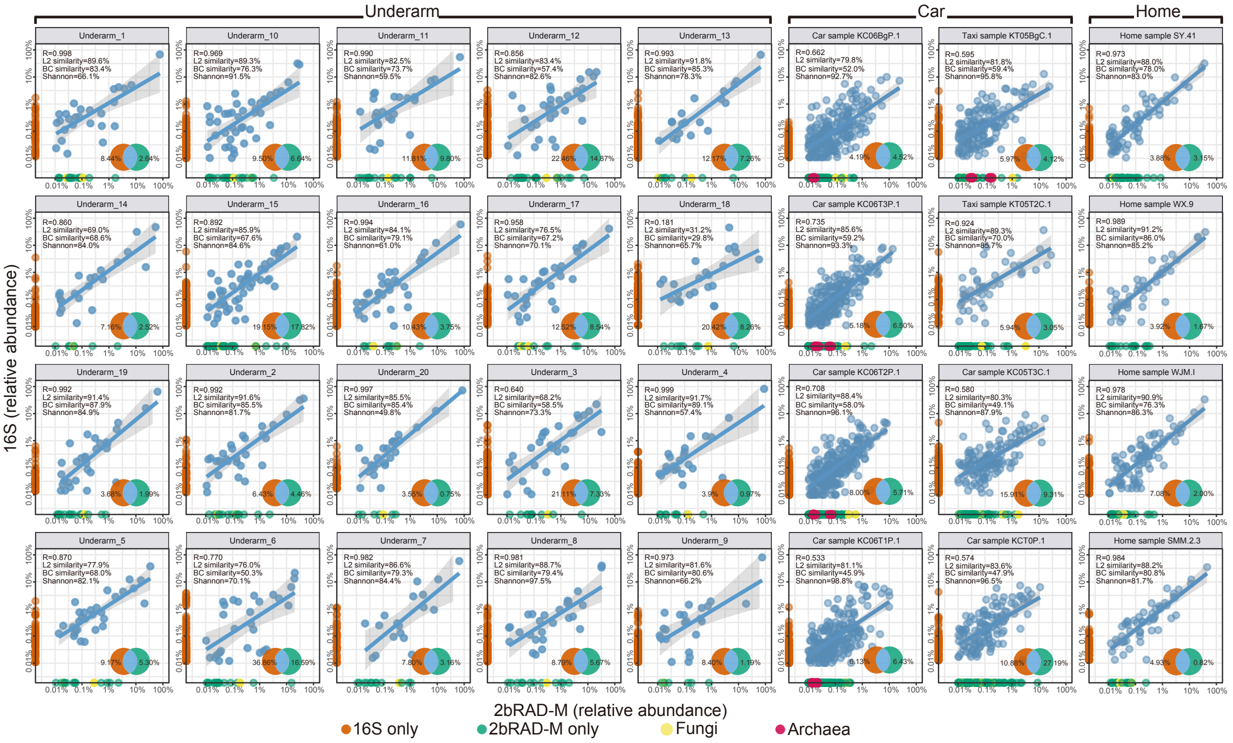

Fig S5. Comparison of the genus-level taxonomic profiles based on 16S rRNA sequencing and 2bRAD-M in each of the underarm, car or home samples. In each scatter plot, blue points represent the genus-level taxa shared between 16S rRNA sequencing and 2bRAD-M, while red points and green points refer to the unique genera identified by 16S rRNA sequencing or 2bRAD-M separately. Each yellow point represents a fungal taxon detected in a given sample by 2bRAD-M. The inset (Venn diagram) shows the overlapping fraction of identified taxa between 16S rRNA and 2bRAD-M profiles. Results for each of the 32 microbiome samples from underarm, car surfaces and home surfaces were presented.

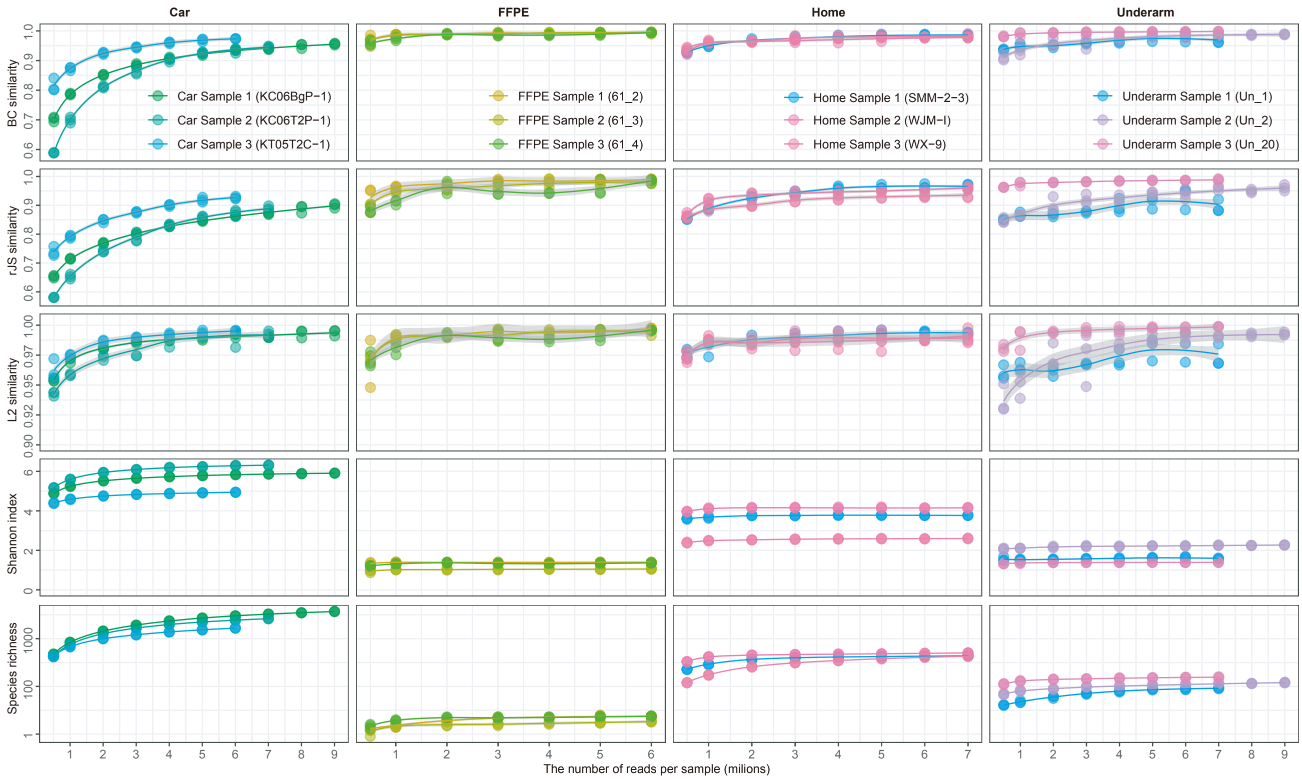

Fig S6. Rarefaction analysis reveals the desirable sequencing depth of 2bRAD-M for taxonomic profiling of the representative built-environment and FFPE samples. From each of the sample categories (car surfaces, FFPE, home surfaces, and underarm skin), three representative samples were shown. For each sample, we compared key performance metrics of the taxonomic profiling (i.e., alpha diversity, beta diversity, and species-level compositions) at several shallow sequencing depths (by subsampling) with those at deep sequencing depth. The scatter plot in each panel indicates the relationship between a performance metric (Y-axis) and the sequencing depth (X-axis).

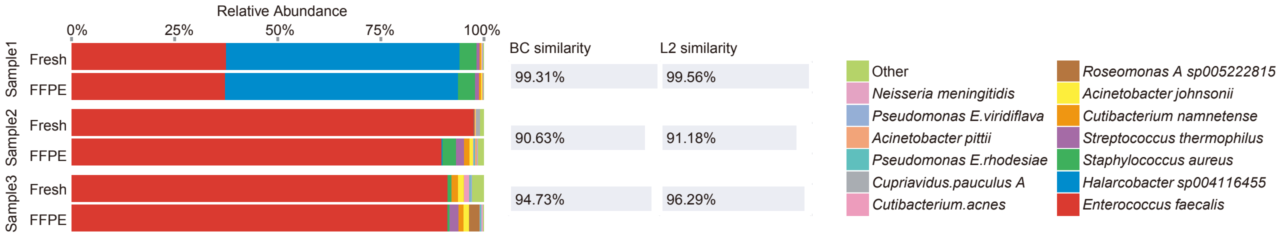

Fig S7. Comparing the taxonomic profiling results of 2bRAD-M between fresh (i.e., pre-FFPE) and post-FFPE lung tissues. Three pairs of healthy lung tissues from lung adenocarcinoma patients, both before and after FFPE processing, were sequenced via 2bRAD-M. Bar plots illustrate their microbial composition. The bars on the same row indicate L2 similarity and BC similarity between each pair of fresh and FFPE samples.

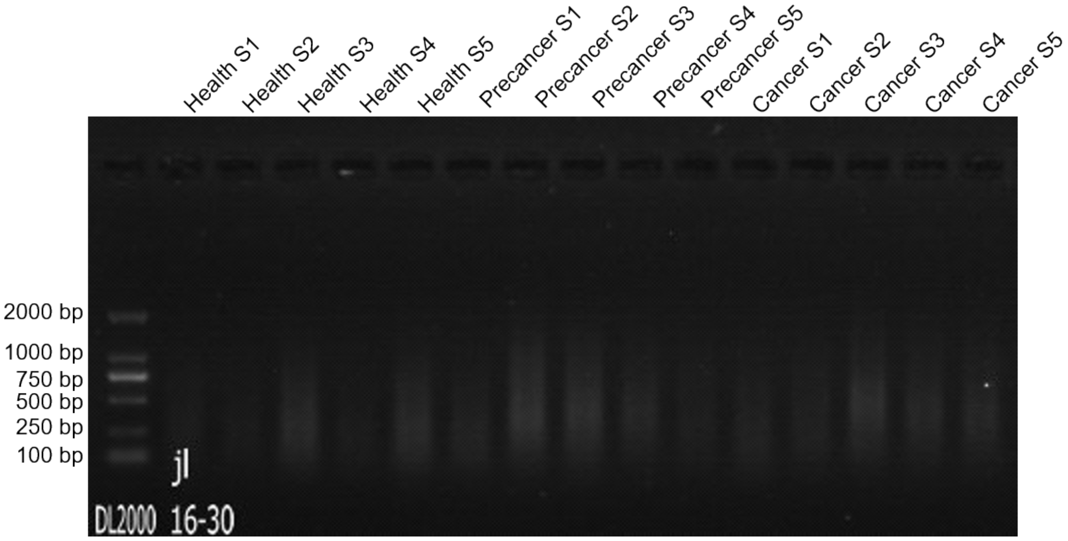

Fig S8. Agarose gel analysis of the DNA extracted from cervical FFPE tissue samples. The quality of DNA from 15 cervical related FFPE tissue samples (five from each group) was assessed by analyzing ~100 ng DNA on a 1% agarose gel at 100V for 25 min.

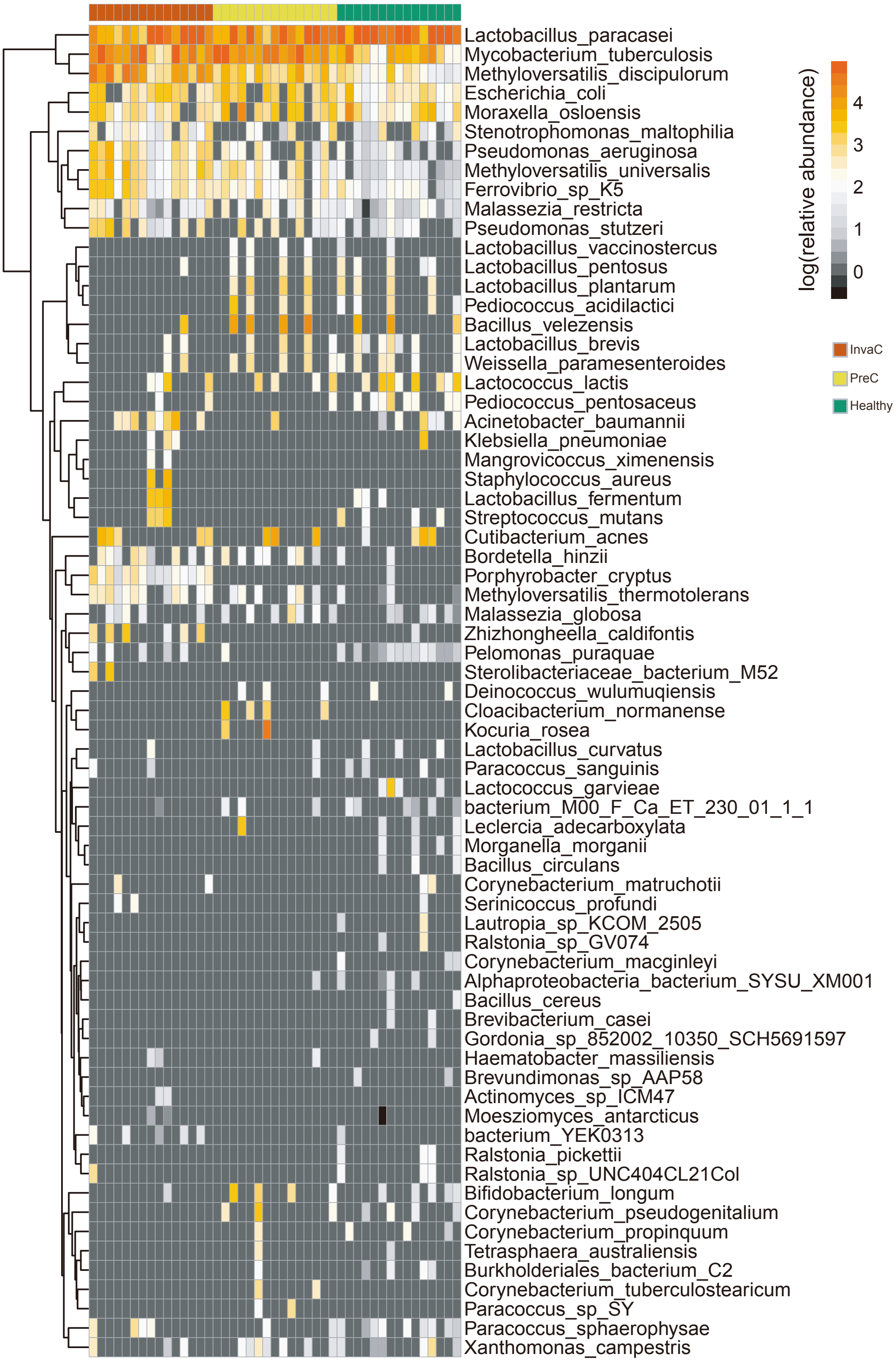

Fig S9. Species abundance profiles of the FFPE samples from healthy tissue, pre-invasive cancer and invasive cancer. The species with a positive importance score in the RF model were presented in the heat map.

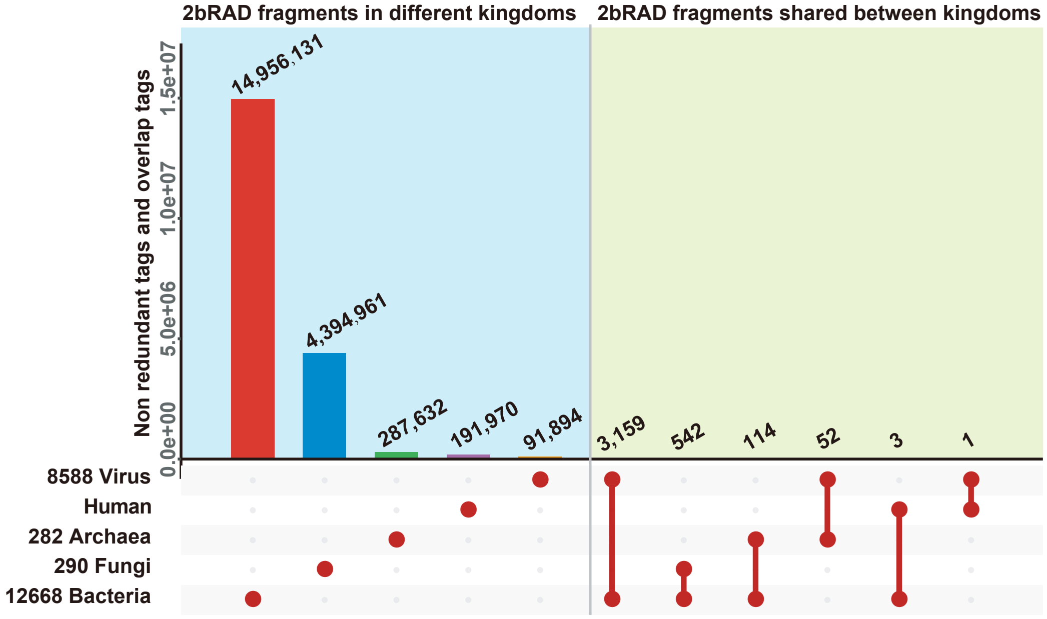

Fig S10. Very few 2bRAD fragments are shared across kingdoms. This *in silico* analysis was attempted to investigate how many 2bRAD fragments are shared across kingdoms or are uniquely identified in certain Kingdom. We collected the complete genomes in RefSeq (8588 virus genomes, 282 archaea genomes, 290 fungi genomes, and 12668 bacteria genomes), and applied BcgI as a representative 2bRAD enzyme to perform this analysis. Human shares almost no 2bRAD fragments with the microbes. Thus, the sequenced 2bRAD fragments from the human host will not interfere with the microbial identification.

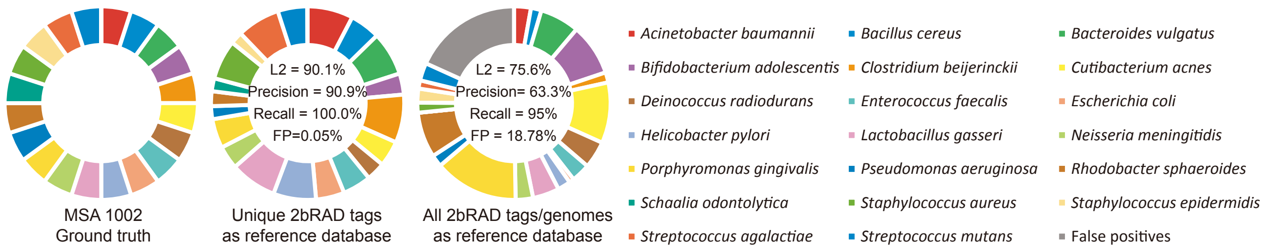

Fig S11. Comparison of MSA 1002 profiling results using different databases. Left panel: the ground truth of MSA 1002. Middle panel: profiling results by the standard 2bRAD-M pipeline (using unique 2bRAD tags as reference database). Right panel: profiling results by using all 2bRAD tags or all microbial genomes (as reference databases).

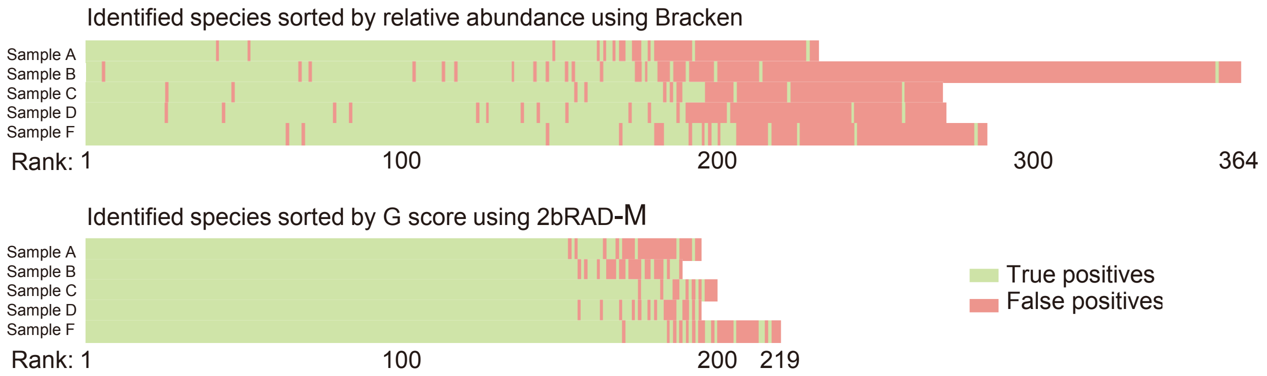

Fig S12. G score provides a higher precision in taxonomic profiling than the relative abundance based on simulated sequencing data. The identified species is ranked by abundance (by Bracken in the upper plot) or G score (by 2bRAD-M in the lower plot), with color indicating whether it is a false positive (FP). Bracken generates far more FPs than 2bRAD-M, as many FPs are in high abundance for Bracken. In contrast, the G score boundary between true positives and FPs are more prominent when using 2bRAD-M.

Supplementary Tables

Table S1. Availability of 2bRAD-M markers for taxonomic profiling at each of the taxonomic levels. In each row, the value indicates the average percentage of taxa-specific 2bRAD-M tags in all 2bRAD-M tags produced by a given type IIB enzyme. Those 2bRAD-M marker tags are all single-copy in a microbial genome and specific to a given taxon. Thus for each of the type IIB enzymes and at each of the taxonomic levels, 2bRAD-M markers or taxonomic profiling are abundant.

IIB enzyme	Phylum	Class	Order	Family	Genus	Species
**AlfI**	89.12%	86.98%	82.84%	79.44%	72.94%	39.79%
**AloI**	86.81%	84.30%	79.41%	75.37%	68.75%	36.85%
**BaeI**	87.50%	85.35%	81.16%	77.76%	71.53%	39.19%
**BcgI**	88.85%	86.64%	82.54%	79.23%	72.71%	39.68%
**BplI**	87.14%	84.58%	80.57%	76.96%	70.17%	38.16%
**BsaXI**	87.39%	85.08%	80.62%	77.03%	70.61%	38.62%
**BslFI**	86.17%	83.96%	79.65%	76.30%	69.95%	37.90%
**Bsp24I**	87.16%	84.88%	80.41%	76.85%	70.22%	38.10%
**CjeI**	87.58%	85.35%	80.99%	77.46%	70.89%	38.57%
**CjePI**	87.85%	85.56%	81.15%	77.51%	70.94%	38.29%
**CspCI**	88.88%	86.68%	82.95%	80.09%	74.09%	41.89%
**FalI**	86.77%	84.24%	79.21%	75.37%	68.45%	37.08%
**HaeIV**	87.43%	85.17%	80.89%	77.35%	70.93%	38.56%
**Hin4I**	86.98%	84.79%	80.52%	77.01%	70.64%	38.56%
**PpiI**	87.93%	85.24%	80.56%	76.55%	69.97%	37.33%
**PsrI**	84.96%	82.43%	77.53%	73.60%	66.78%	36.28%

Table S2. Expected abundance of bacterial species in simulation data and profiling results from the 2bRAD-M computational pipeline.

Organism Name	Assembly Accession	Relative abundance	2bRAD-M
***Archaeoglobus fulgidus* DSM 4304**	GCF_000008665.1	0.667%	0.673%
***Clostridium acetobutylicum* ATCC 824**	GCF_000008765.1	0.667%	0.000%
***Lactobacillus salivarius* UCC118**	GCF_000008925.1	0.667%	0.716%
***Ralstonia solanacearum* GMI1000**	GCF_000009125.1	0.667%	0.663%
***Nitrosomonas europaea* ATCC 19718**	GCF_000009145.1	0.667%	0.697%
***Helicobacter acinonychis str.* Sheeba**	GCF_000009305.1	0.667%	0.704%
***Yersinia enterocolitica subsp. enterocolitica* 8081**	GCF_000009345.1	0.667%	0.659%
***Alcanivorax borkumensis* SK2**	GCF_000009365.1	0.667%	0.657%
***Streptococcus uberis* 0140J**	GCF_000009545.1	0.667%	0.648%
***Staphylococcus haemolyticus* JCSC1435**	GCF_000009865.1	0.667%	0.758%
Symbiobacterium thermophilum IAM 14863	GCF_000009905.1	1.333%	1.370%
***Chlamydia felis* Fe/C-56**	GCF_000009945.1	1.333%	1.315%
***Thermococcus kodakarensis* KOD1**	GCF_000009965.1	1.333%	1.368%
***Magnetospirillum magneticum* AMB-1**	GCF_000009985.1	1.333%	1.372%
***Synechococcus elongatus* PCC 6301**	GCF_000010065.1	1.333%	1.230%
Sodalis glossinidius str. 'morsitans'	GCF_000010085.1	1.333%	1.311%
***Finegoldia magna* ATCC 29328**	GCF_000010185.1	1.333%	1.428%
***Gemmatimonas aurantiaca* T-27**	GCF_000010305.1	1.333%	1.317%
***Nitratiruptor sp.* SB155-2**	GCF_000010325.1	1.333%	1.298%
***Sulfurovum sp.* NBC37-1**	GCF_000010345.1	1.333%	1.330%
***Bifidobacterium adolescentis* ATCC 15703**	GCF_000010425.1	2.000%	1.908%
***Porphyromonas gingivalis* ATCC 33277**	GCF_000010505.1	2.000%	1.981%
***Azorhizobium caulinodans* ORS 571**	GCF_000010525.1	2.000%	2.018%
***Macrococcus caseolyticus* JCSC5402**	GCF_000010585.1	2.000%	2.076%
Candidatus Azobacteroides pseudotrichonymphae genomovar. CFP2	GCF_000010645.1	2.000%	2.590%
***Acetobacter pasteurianus IFO* 3283-01**	GCF_000010825.1	2.000%	2.048%
***Deferribacter desulfuricans* SSM1**	GCF_000010985.1	2.000%	2.011%
***Pyrococcus horikoshii* OT3**	GCF_000011105.1	2.000%	2.058%
***Thermoplasma volcanium* GSS1**	GCF_000011185.1	2.000%	1.996%
***Mycoplasma penetrans* HF-2**	GCF_000011225.1	2.000%	2.035%
***Oceanobacillus iheyensis* HTE831**	GCF_000011245.1	2.667%	2.651%
***Thermosynechococcus elongatus* BP-1**	GCF_000011345.1	2.667%	2.695%
***Gloeobacter violaceus* PCC 7421**	GCF_000011385.1	2.667%	2.657%
***Ruegeria pomeroyi* DSS-3**	GCF_000011965.2	2.667%	2.626%
***Rickettsia felis* URRWXCal2**	GCF_000012145.1	2.667%	2.720%
***Psychrobacter arcticus* 273-4**	GCF_000012305.1	2.667%	2.640%
***Thermobifida fusca* YX**	GCF_000012405.1	2.667%	2.803%

***Dechloromonas aromatica* RCB**	GCF_000012425.1	2.667%	2.641%
***Pelodictyon luteolum* DSM 273**	GCF_000012485.1	2.667%	2.627%
***Synechococcus sp.* CC9902**	GCF_000012505.1	2.667%	2.642%
***Methanosphaera stadtmanae* DSM 3091**	GCF_000012545.1	3.333%	3.357%
***Ehrlichia canis str.* Jake**	GCF_000012565.1	3.333%	3.232%
***Chlorobium chlorochromatii* CaD3**	GCF_000012585.1	3.333%	3.308%
***Nitrobacter winogradskyi* Nb-255**	GCF_000012725.1	3.333%	3.073%
***Nitrosococcus oceani* ATCC 19707**	GCF_000012805.1	3.333%	3.387%
***Carboxydothermus hydrogenoformans* Z-2901**	GCF_000012865.1	3.333%	3.351%
***Pelobacter carbinolicus* DSM 2380**	GCF_000012885.1	3.333%	3.375%
***Sulfurimonas denitrificans* DSM 1251**	GCF_000012965.1	3.333%	3.292%
Alternaria arborescens	GCF_004154835.1	3.333%	3.334%
Apiotrichum porosum	GCF_003942205.1	3.333%	3.345%
Alternaria_alternata	GCF_001642055.1	0.000%	0.006%

Table S3. The relative enrichment of 2bRAD reads originated from microbial species versus those originated from host in the high-host-contamination (HoC) group.

Group	*Sample*	*Raw reads*	*Host reads*	*Multi-and un-mapped reads*	*Reads mapped to the microbiome species*	*Read utility rate*
90% human DNA	Repeat1	11,709,616	5,042,885	3,654,108	3,012,623	25.73%
	Repeat2	8,311,076	2,931,226	2,762,238	2,617,612	31.50%
	Repeat3	8,337,960	2,896,376	2,739,925	2,701,659	32.40%
99% human DNA	Repeat1	8,290,184	5,515,764	2,469,455	304,965	3.68%
	Repeat2	11,699,970	7,742,314	3,565,094	392,562	3.36%
	Repeat3	11,736,005	8,118,716	3,150,525	466,764	3.98%
FFPE	Ca_1	6,052,247	1,718,081	72,573	4,261,593	1.20%
	Ca_2	4,940,545	1,033,198	361,893	3,545,454	7.32%
	Ca_3	5,346,458	1,573,697	76,608	3,696,153	1.43%
	Ca_4	4,986,484	1,404,204	128,123	3,454,157	2.57%
	Ca_5	5,645,600	1,514,926	267,627	3,863,047	4.74%
	Ca_6	7,208,166	2,061,466	187,901	4,958,799	2.61%
	Ca_7	7,069,044	1,623,372	560,097	4,885,575	7.92%
	Ca_8	7,527,292	2,335,492	221,045	4,970,755	2.94%
	Ca_9	7,134,800	1,619,165	595,266	4,920,369	8.34%
	Ca_10	6,806,243	1,855,923	212,906	4,737,414	3.13%
	Ca_11	6,895,623	2,002,943	180,056	4,712,624	2.61%
	Ca_12	6,871,262	1,651,782	478,093	4,741,387	6.96%
	Ca_13	7,298,871	1,966,508	376,338	4,956,025	5.16%
	Ca_14	6,891,844	1,622,370	541,501	4,727,973	7.86%
	Ca_15	6,494,796	1,452,886	419,561	4,622,349	6.46%
	CIN_1	5,879,280	1,520,802	152,322	4,206,156	2.59%
	CIN_2	6,018,984	1,339,554	381,999	4,297,431	6.35%
	CIN_3	1,231,440	283,532	49,456	898,452	4.02%
	CIN_4	5,893,353	1,389,855	300,987	4,202,511	5.11%
	CIN_5	5,861,334	1,237,725	279,815	4,343,794	4.77%
	CIN_6	1,069,641	205,399	82,358	781,884	7.70%
	CIN_7	4,763,819	988,823	237,228	3,537,768	4.98%
	CIN_8	5,654,689	1,316,310	236,328	4,102,051	4.18%
	CIN_9	4,844,447	1,305,035	131,859	3,407,553	2.72%
	CIN_10	5,674,624	1,406,368	207,917	4,060,339	3.66%
	CIN_11	1,864,811	430,781	67,901	1,366,129	3.64%
	CIN_12	5,520,880	1,351,240	233,013	3,936,627	4.22%
	CIN_13	948,043	227,049	31,840	689,154	3.36%
	CIN_14	5,795,588	1,465,060	189,249	4,141,279	3.27%
	CIN_15	4,985,844	1,296,516	127,127	3,562,201	2.55%
	Nor_1	2,068,027	440,215	67,120	1,560,692	3.25%
	Nor_2	6,237,924	1,528,373	175,528	4,534,023	2.81%

FFPE	Nor_3	6,292,584	1,595,949	153,430	4,543,205	2.44%
	Nor_4	6,365,808	1,505,140	253,251	4,607,417	3.98%
	Nor_5	6,206,474	1,621,247	91,532	4,493,695	1.47%
	Nor_6	6,343,568	1,708,611	129,338	4,505,619	2.04%
	Nor_7	5,665,788	1,227,457	263,104	4,175,227	4.64%
	Nor_8	4,633,962	1,021,751	141,919	3,470,292	3.06%
	Nor_9	5,109,883	1,252,051	93,516	3,764,316	1.83%
	Nor_10	5,921,836	1,383,617	273,317	4,264,902	4.62%
	Nor_11	5,447,467	1,346,470	162,437	3,938,560	2.98%
	Nor_12	5,565,365	1,306,203	244,443	4,014,719	4.39%
	Nor_13	4,063,180	782,890	79,408	3,200,882	1.95%
	Nor_14	4,901,906	1,053,036	159,043	3,689,827	3.24%
	Nor_15	5,588,470	1,279,742	156,579	4,152,149	2.80%

Table S4. The relative abundance of major taxa identified in the three fecal samples at the species level using 2bRAD-M or WMS or at the genus level using 16S rRNA gene amplicon sequencing.

Real fecal sample A							
**Top species in 2bRAD-M**	**Relative abundance**	**Rank**	**Corresponding relative abundance in WMS**	**Rank**	**Corresponding genus in 16S**	**Relative abundance**	**Rank**
Prevotella copri	61.86%	1	60.98%	1	*Prevotella*	71.14%	1
Prevotella sp BCRC	10.81%	2	9.46%	2	*Prevotella*	71.14%	1
Prevotella stercorea	4.85%	3	6.12%	3	*Prevotella*	71.14%	1
Bacteroides plebeius	2.97%	4	2.97%	4	*Bacteroides*	16.44%	
Bacteroides coprophilus	1.46%	5	1.48%	6	*Bacteroides*	16.44%	
Bacteroides uniformis	1.37%	6	1.72%	5	*Bacteroides*	16.44%	2
Bacteroides coprocola	1.31%	7	1.22%	7	*Bacteroides*	16.44%	
Bacteroides thetaiotaomicron	0.95%	8	0.79%	10	*Bacteroides*	16.44%	
Acinetobacter baumannii	0.81%	9	1.18%	8	*NA*	NA	NA
Bacteroides stercoris	0.71%	10	0.69%	11	*Bacteroides*	16.44%	2
Alistipes putredinis	0.69%	11	1.03%	9	*Alistipes*	0.89%	6
Prevotella sp AM23 5	0.65%	12	0.45%	15	*Prevotella*	71.14%	1
Parabacteroides merdae	0.61%	13	0.64%	12	*Parabacteroides*	1.93%	4
Parabacteroides distasonis	0.59%	14	0.54%	13	*Parabacteroides*	1.93%	4
Bacteroides massiliensis	0.57%	15	0.50%	14	*Bacteroides*	16.44%	2
Bacteroides vulgatus	0.47%	16	0.43%	16	*Bacteroides*	16.44%	2
Eubacterium rectale	0.41%	17	0.39%	17	*NA*	NA	NA
Megamonas funiformis	0.38%	18	0.21%	30	*Megamonas*	0.71%	9
Phascolarctobacterium succinatutens	0.33%	19	0.38%	18	*Phascolarctobacterium*	0.56%	11
Bacteroides salyersiae	0.31%	20	0.27%	23	*Bacteroides*	16.44%	2
**SUM**	92.10%		91.43%			91.67%	
**Real fecal sample B**							
**Top species in 2bRAD-M**	**Relative abundance**	**Rank**	**Corresponding relative abundance in WMS**	**Rank**	**Corresponding genus in 16S**	**Relative abundance**	**Rank**
Prevotella copri	58.00%	1	55.21%	1	*Prevotella*	69.47%	1
Prevotella sp BCRC	7.23%	2	7.44%	2	*Prevotella*	69.47%	1
Prevotella stercorea	4.36%	3	6.25%	3	*Prevotella*	69.47%	1
Bacteroides coprophilus	4.26%	4	4.57%	4	*Bacteroides*	13.49%	2
Acinetobacter baumannii	3.13%	5	1.38%	9	*NA*	NA	NA
Eubacterium rectale	2.83%	6	2.47%	5	*NA*	NA	NA
Bacteroides plebeius	1.72%	7	1.77%	6	*Bacteroides*	13.49%	2

Bacteroides vulgatus	1.59%	8	1.54%	8	*Bacteroides*	13.49%	2
Bacteroides dorei	1.16%	9	1.05%	13	*Bacteroides*	13.49%	2
Bacteroides uniformis	1.10%	10	1.24%	11	*Bacteroides*	13.49%	2
Parabacteroides merdae	1.00%	11	1.19%	12	*Parabacteroides*	1.86%	4
Alistipes putredinis	0.93%	12	1.57%	7	*Alistipes*	0.81%	9
Lachnospira pectinoschiza	0.71%	13	0.46%	19	*NA*	NA	NA
Sutterella sp KLE1602	0.64%	14	1.29%	10	*Sutterella*	1.26%	7
Bacteroides stercoris	0.58%	15	0.65%	14	*Bacteroides*	13.49%	2
Parabacteroides distasonis	0.56%	16	0.58%	16	*Parabacteroides*	1.86%	4
Prevotella sp Marseille	0.51%	17	0.60%	15	*Prevotella*	69.47%	1
Bacteroides caccae	0.49%	18	0.47%	18	*Bacteroides*	13.49%	2
Dialister sp Marseille	0.46%	19	0.36%	25	*Dialister*	0.01%	36
Megamonas funiformis	0.43%	20	0.40%	20	*Megamonas*	1.70%	5
**SUM**	91.67%		90.47%			88.60%	

Real fecal sample C

Top species in 2bRAD-M	Relative abundance	Rank	Corresponding relative abundance in WMS	Rank	Corresponding genus in 16S	Relative abundance	Rank
Prevotella copri	29.07%	1	28.97%	1	*Prevotella*	29.30%	1
Bacteroides massiliensis	8.35%	2	6.69%	3	*Bacteroides*	24.98%	2
Bacteroides dorei	6.28%	3	5.05%	5	*Bacteroides*	24.98%	2
Faecalibacterium prausnitzii	5.75%	4	9.16%	2	*Faecalibacterium*	11.65%	3
Bacteroides plebeius	5.17%	5	5.68%	4	*Bacteroides*	24.98%	2
Bacteroides uniformis	3.78%	6	3.92%	7	*Bacteroides*	24.98%	2
Roseburia intestinalis	3.29%	7	2.49%	8	*Roseburia*	8.48%	4
Alistipes putredinis	3.03%	8	5.04%	6	*Alistipes*	2.05%	8
Roseburia inulinivorans	2.95%	9	1.90%	9	*Roseburia*	8.48%	4
Lachnospira pectinoschiza	2.43%	10	1.32%	10	*NA*	NA	NA
Clostridium sp AM42	1.35%	11	1.29%	11	*Clostridium*	3.12%	7
Clostridium sp AF43	1.23%	12	0.89%	17	*Clostridium*	3.12%	7
Bacteroides caccae	1.13%	13	0.95%	16	*Bacteroides*	24.98%	2
Roseburia faecis	1.11%	14	0.79%	21	*Roseburia*	8.48%	4
Bacteroides sp	1.08%	15	1.07%	13	*Bacteroides*	24.98%	2
Eubacterium rectale	1.03%	16	1.02%	14	*NA*	NA	NA
Firmicutes bacterium OM08	1.02%	17	0.60%	25	*NA*	NA	NA
Paraprevotella clara	1.00%	18	1.13%	12	*Paraprevotella*	1.41%	11
Firmicutes bacterium AF22	0.96%	19	0.59%	26	*NA*	NA	NA
Clostridium sp OM08	0.93%	20	0.99%	15	*Clostridium*	3.12%	7
**SUM**	80.94%		79.53%			80.97%	

Table S5. The relative abundance of microbial species that are uniquely detected in the WMS or 2bRAD-M data of fecal samples. These species account for a very small proportion (<0.5%) in the fecal microbiota.

WMS only (relative abundance of species)					
**fecal sample A**		**fecal sample B**		**fecal sample c**	
**Megamonas rupellensis**	0.000417	*Pseudomonas aeruginosa*	0.000388	*Prevotella sp AM23_5*	0.000563
**Fusicatenibacter saccharivorans**	0.000226	*Alistipes sp AL_1*	0.000343	*Clostridium sp AF37_5AT*	0.000401
**Bacteroides sp 3_1_40A**	0.000201	*Collinsella sp AF39_11AT*	0.000329	*Clostridiales bacterium VE202_03*	0.000242
**Blautia sp AM23_13AC**	0.000156	*Butyricicoccus sp AF24_19AC*	0.000323	*Faecalibacterium sp_An58*	0.000241
**Prevotella multiformis**	0.000142	*Megamonas hypermegale*	0.000250	*Prevotella bryantii*	0.000233
**Ruminococcus sp AF25_19**	0.000140	*Butyricicoccus sp OM06_6AC*	0.000221	*Alistipes sp 3BBH6*	0.000184
**Ruminococcus torques**	0.000132	*Ruminococcus sp AF19_29*	0.000213	*Collinsella aerofaciens*	0.000171
**Ruminococcus sp AF21_11**	0.000132	*Butyricicoccus sp AM29_23AC*	0.000166	*Blautia sp AM47_4*	0.000161
**Bilophila wadsworthia**	0.000122	*Oscillospiraceae bacterium_VE202_24*	0.000162	*Clostridium sp AF27_5AA*	0.000158
**Collinsella aerofaciens**	0.000119	*Evtepia gabavorous*	0.000162	*Faecalibacterium sp_An121*	0.000148
**Blautia sp AF22_5LB**	0.000113	*Bacteroides sp AM56_10ce*	0.000149	*Phocea massiliensis*	0.000147
**Butyricicoccus sp AM27_36**	0.000111	*Ruminococcus sp AF37_3AC*	0.000146	*Prevotella sp Marseille P4119*	0.000144
**Subdoligranulum sp OF01_18**	0.000102	*Prevotella sp P5_60*	0.000145	*Alistipes ihumii*	0.000142
		Streptococcus salivarius	0.000137	*Clostridium sp_SN20*	0.000136
		Faecalibacterium sp OM04_11BH	0.000136	*Butyricicoccus sp AM27_36*	0.000131
		Desulfotomaculum sp OF05_3	0.000132	*Alistipes obesi*	0.000127
		Blautia sp OF03_13	0.000128	*Tidjanibacter massiliensis*	0.000120
		Blautia sp OM06_15AC	0.000121	*Bacteroides sp AM16_13*	0.000108
		Bacteroides fragilis	0.000118	*Clostridium asparagiforme*	0.000107
		Collinsella sp OF03_4AA	0.000116	*Ruminococcaceae bacterium AM28_23LB*	0.000106
		Clostridiaceae bacterium TF01_6	0.000108	*Butyricicoccus sp AF10_3*	0.000103
		Prevotella sp Marseille P4119	0.000105	*Parabacteroides goldsteinii*	0.000101
		Lachnospiraceae bacterium 7_1_58FAA	0.000104		
**Sum**	**0.21%**	**Sum**	**0.42%**	**Sum**	**0.40%**
**2bRAD-M only (relative abundance of species)**					
**fecal sample A**		**fecal sample B**		**fecal sample c**	
**Romboutsia timonensis**	0.000181	*Bacteroides sp AF34_31BH*	0.000206	*Eubacterium sp AM49_13BH*	0.000235
**Eubacteriaceae bacterium**	0.000138	*Collinsella sp OM08_14AT*	0.000145	*Subdoligranulum sp AM16_9*	0.000121
**Bacteroides sp 1_1_14**	0.000137	*Bacteroides sp A1C1*	0.000137	*Bacteroides sp AM56_10ce*	0.000120
**Clostridiales bacterium KLE1615**	0.000131	*Subdoligranulum sp AM16_9*	0.000115	*Prevotella sp AM34_19LB*	0.000104
**Bacteroides nordii**	0.000108	*Clostridium phoceensis*	0.000110	*Prevotella sp BCRC_81118*	0.000101
**Blautia sp AF19_10LB**	0.000106	*Blautia sp TM10_2*	0.000109		
**Sum**	**0.08%**	**Sum**	**0.08%**	**Sum**	**0.07%**

Table S6. The initial DNA content and metadata for underarm skin, home and car samples.

Sample ID	Gender	Age	Ethnicity	Sampling Site	Concentration ng/μL(Qubit)
**UA_01**	Female	26-35	Chinese	Underarm	0.5414
**UA_02**	Female	46-55	Indian	Underarm	0.09839
**UA_03**	Male	26-35	Chinese	Underarm	0.06578
**UA_04**	Female	46-55	Chinese	Underarm	1.489
**UA_05**	Male	26-35	Indian	Underarm	2.421
**UA_06**	Male	26-35	Filipino	Underarm	3.924
**UA_07**	Female	36-45	Indian	Underarm	0.5529
**UA_08**	Male	26-35	Chinese	Underarm	4.182
**UA_09**	Female	26-35	Indian	Underarm	24.49
**UA_10**	Male	36-45	Chinese	Underarm	1.535
**UA_11**	Female	36-45	Chinese	Underarm	1.472
**UA_12**	Male	26-35	Chinese	Underarm	0.1819
**UA_13**	Female	26-35	Filipino	Underarm	4.339
**UA_14**	Male	18-25	Indian	Underarm	2.096
**UA_15**	Female	46-55	Filipino	Underarm	4.911
**UA_16**	Male	46-55	Filipino	Underarm	3.938
**UA_17**	Male	46-55	Filipino	Underarm	1.793
**UA_18**	Male	26-35	Chinese	Underarm	1.214
**UA_19**	Male	36-45	Indian	Underarm	1.284
**UA_20**	Male	36-45	Indian	Underarm	1.379
**Car_KC05T3C-1**	NA	NA	NA	Cushion in car	10.99
**Car_KC06BgP-1**	NA	NA	NA	Floor mat in car	20.28
**Car_KC06T1P-1**	NA	NA	NA	Floor mat in car	35.84
**Car_KC06T2P-1**	NA	NA	NA	Floor mat in car	9.04
**Car_KC06T3P-1**	NA	NA	NA	Floor mat in car	11.83
**Car_KCT0P-1**	NA	NA	NA	Floor mat in car	19.22
**Car_KT05BgC-1**	NA	NA	NA	Cushion in car	11.96
**Car_KT05T2C-1**	NA	NA	NA	Cushion in car	10.43
**Home_SMM-2-3**	NA	NA	NA	Child’s book	5.231
**Home_SY-41**	NA	NA	NA	Toilet	13.49
**Home_WJM-I**	NA	NA	NA	Child’s toy	9.917
**Home_WX-9**	NA	NA	NA	Toilet mat	21.77

Table S7. The relative abundance of bacteria, fungi and archaea in the indoor built-environmental samples.

Kingdom	Underarm	Home	Car
**Bacteria**	99.69%	99.93%	98.71%
**Fungi**	0.31%	0.07%	1.26%
**Archaea**	0.00%	0.001%	0.03%

Table S8. Species-level microbial organismal markers for the highly reliable diagnosis of cervical cancer from FFPE samples.

**Species name**	**Mean abundance**			**Importance**
	InvaC	PreC	Health	
Porphyrobacter cryptus	0.297%	0.000%	0.002%	0.200911002
Pelomonas puraquae	1.487%	0.437%	0.039%	0.057330947
Methyloversatilis discipulorum	13.050%	2.573%	0.547%	0.056879324
Methyloversatilis universalis	0.024%	0.011%	0.016%	0.052514646
Pseudomonas aeruginosa	1.161%	0.233%	0.039%	0.029690798
Mycobacterium tuberculosis	25.149%	28.064%	7.960%	0.02625783
Escherichia coli	31.957%	33.057%	62.531%	0.018157477
Lactobacillus paracasei	1.251%	1.748%	0.686%	0.012936985
Ferrovibrio sp K5	1.187%	0.350%	0.129%	0.010616483

Table S9. The adaptors and primers used in 2bRAD-M sequencing (5’-3’).

Name	Adaptor sequence
**Adap-1 sense**	ACACTCTTTCCCTACACGACGCTCTTCCGATCTNN
**Adap-2 sense**	GTGACTGGAGTTCAGACGTGTGCTCTTCCGATCTNN
**Adap antisense**	AGATCGGAAGAGC
	**Primer sequence**
**Primer1**	ACACTCTTTCCCTACACGACGCT
**Primer2**	GTGACTGGAGTTCAGACGTGTGCT
**Primer3**	AATGATACGGCGACCACCGAGATCTACACTCTTTCCCTACACGACGCT
**Index primer**	CAAGCAGAAGACGGCATACGAGATXXXXXXGTGACTGGAGTTCAGACGTGT
