# Supplementary material for: Species-resolved sequencing of low-biomass or degraded microbiomes using 2bRAD-M
Source: Genome Biol. 2022 Jan 26;23:36. doi: 10.1186/s13059-021-02576-9 (PMC8789378; doi:10.1186/s13059-021-02576-9)
Supplement: Supplementary file 3 — Additional file 3. Review history [file 13059_2021_2576_MOESM3_ESM.docx]

Review History

**First round of review**

**Reviewer 1**

**Were you able to assess all statistics in the manuscript, including the appropriateness of statistical tests used?**

Yes

**Were you able to directly test the methods?**

No

**Comments to author:**

In this manuscript, Sun et al. performed a comprehensive comparison of the 2bRAD-M method with whole-metagenome sequencing (WMS). The new method utilizes type IIB restriction endonucleases for sequencing of metagenomes and identifying microbial compositions. The tested method was previously introduced in 2012 for genotyping (Shi Wang et al., Nat. Methods 2012), and was optimized later on for NGS (Shi Wang et al. Nat. Protoc 2016), and generates short and identical length fragments (tags) that are used as genus- and species-specific markers. Sequencing of these markers is less costly than shotgun sequencing, while allowing for increased sensitivity that out-performs the 16s amplicon sequencing. The method proposes to tackle challenges related to I. samples with low amounts of DNA (LoA) II. Host DNA contamination (HoC) and III. High DNA degradation (HiD). The authors created a unique marker-specific database generated on ~170,000 bacteria, archaea and fungi genomes from the refseq database, by extracting the segments where the restriction enzymes would cut. Then they performed a computational simulation to validate the precision and recall of the proposed method and database in comparison to existing shotgun. They then performed a mock sample-based sequencing (on five bacterial genomes) and compared WMS, amplicon and 2bRAD-M sequencing in a 20-species known-composition community followed by validation of stool samples with WMS and low-biomass samples with amplicon sequencing. Finally, the authors performed 2bRAD-M on biopsies from cervical cancer preserved in paraffin and compared healthy, benign and malignant-sequenced microbiome compositions.

Overall, the 2bRAD-M method has the potential to elegantly improve the above challenges. However, there are major concerns in the current manuscript that must be addressed to reach an adequate stringency and reproducibility of the presented method.

Major issues

1. Host removal - While the method is proposed to tackle host contamination (HoC), and is shown to do so in controlled environments (a five-species bacterial community), the authors do not disclose how the method would perform under these circumstances. Importantly, the authors neither report on a step of host removal, which we assume is mandatory in this scenario, nor do they report the funnel of reads from raw reads -> host mapped reads -> bacteria mapped reads and percentage of unmapped reads, which are all vital in benchmarking this method. The authors are requested to:

a. report how host removal was done, or perform host removal steps if it was not done.

b. report the described reads funnel for each sequenced sample in order to allow for a fair comparison.

c. Provide motivation and explanation to why this method has the potential to overcome shotgun metagenomic ability to handle HoC data.

2. Computational simulations - several critical issues need to be addressed:

a. Present simulation seems not to constitute a fair comparison, as the database was built in the exact manner as the simulated sequences were made, giving it an unfair advantage. As such, this approach does not shed additional corroborative light on the 2bRAD-M method, but solely on the database itself. To solve this inherent flaw, the tested method should be compared to the most similar database possible. We request the authors to use the ~170,000 that were used to create the 2bRAD-M database to build a database to be used by kraken/bracken and Centrifuge.

b. There is no description in the methods to which databases kraken, bracken and Centrifuge were applied to and which parameters were used (neither for Metaphlan, but a pre-built database exist in this scenario). We ask the authors to describe the full exact nature in which this simulation was performed.

c. It is not clear how kraken and Centrifuge had such different results as they are practically algorithmically identical.

3. Fecal sample sequencing - There are major data inconsistencies in the data provided in table S3 and figure 4, and missing information related to shotgun sequenced samples.

a. In S3, Eubacterium rectale is has 0.41% relative abundance and ranked 178 in the shotgun sequenced method, while there are bacteria with lower abundance that ranked higher. Please clarify this issue.

b. In figure 4d-f, it seems that there are only a few bacteria that had high relative abundance and were not detected by 2bRAD-M, while the tables suggest otherwise. Please clarify this issue.

c. It is not clear which database was used in this section and which method. In order to provide the proper comparison, with the same exact taxa, we request you use the kraken/bracken/Centrifuge methods, built using the same database that use used for the 2bRAD-M database.

d. Please provide detailed information regarding the bacteria that were only detected in the shotgun method.

4. 2bRAD-M database - While the generation of a specific database for the 2bRAD-M is indeed useful, it is not explored enough to understand its validity, specifically. Please disclose the following:

a. Distribution of the amount of tags for each species/genus/phylum.

b. Distribution of unique amount of tags for each species/genus/ phylum to detect under- and over-represented bacterial genomes.

Minor issues

1. Metrics - while the usage of l2 similarity is not discouraged, please inlcude alpha- and beta-diversity when handling biological and mock samples, as these are commonly used.

2. It is mentioned in the introduction that the amount of DNA needed for sequencing is at least 20ng, while it is common knowledge that even 1ng is enough for shotgun, and even less is sufficient for 16s. please correct.

3. Why weren't all the WMS tools tested against the 20-bacteria mock-community? Please add a comparison for all tools.

4. Other tags are mentioned but not practically tested. What would be expected if a few tags would be used in a combined manner? Wouldn't such an approach increase specificity?

5. As mentioned above, it is unclear which database reference was used from only the citation of the shallow metagenomic paper. A reference should be built for both kraken/bracken and Centrifuge from the refseq (the same exact genomes that were used for the tag db) for a complete and fair comparison.

6. On what data was the Pearson correlation calculated? Does it include bacteria that weren't discovered? It seems that it doesn't take them into account, and it should contain them, or properly explained in the text if it does not.

7. It seems that the rarefaction curves in S2 are not in conjunction with the text. In the Shannon diversity, the samples are not close to the alpha diversity of the full length, while the species richness does not reach a stratification (neither for WMS). Could OTUs be calculated following filtration of extremely low abundant species?

8. The scales of the rarefaction curves are unfairly plotted against the shotgun sequencing method. As the diversity measures are measured with respect to 400M reads or 15M reads. In order to provide a fair comparison, 15M samples should be sequenced and calculated or subsampled for 15M reads.

9. The dendrograms for the hierarchical clustering in figures 5a-b should be provided and explained

10. was species resolution demonstrated for 16s? It is claimed that the signal of s. epidermidis wouldn't be seen, but it might be captured by 16s.

11. The 266 line supports robustness of the 2bRAD-M method, but the figures suggest otherwise.

**Reviewer 2**

**Were you able to assess all statistics in the manuscript, including the appropriateness of statistical tests used?**

Yes: All statistics used was appropriate

**Were you able to directly test the methods?**

No

**Comments to author:**

The authors of GBIO-D-21-00210 submission, Sun et al, present a novel method 2bRAD-M for profiling microbial taxonomic composition particularly suitable for samples characterized by low biomass, poor DNA quality (high fragmentation) and highly contaminated with host DNA. The method combines clever experimental and computational design that builds of authors' previous work on enzymatic cleavage of DNA from any organism into predictable fragments suitable for amplification, sequencing and classification at species and higher taxonomic levels. As such, it is a timely a timely addition to the repertoire of reference-based microbiome profiling approaches particularly suited for attractive but hard to work with samples (e.g. biopsies).

While the authors present the intuition behind 2bRAD-M very clearly and I am convinced about the applicability of their approach to the proposed scenarios, they made some questionable choices when evaluating it against mock communities. Also, the presentation of the microbiome profiling in the preserved tissue samples (FFPE) lacks any validation or comparison with other profiling approach (e.g. 16S rRNA), making it impossible to evaluate the 2bRAD-M's performance in these valuable samples. I feel strongly that both these points would need to be addressed so that the manuscript presents a coherent story. These and other comments are elaborated on below.

Main points:

1. The choice of Centrifuge for profiling the mock community ATCC MSA1002 with 20 species in Fig. 3b is unfortunate, as you yourself presented in the simulated data in Fig. 2b that Centrifuge is the worst performing among all methods you tested (poor recall, precision and L2 similarity). Rather, please present the results for one of the better performing profilers (e.g. MetaPhlan2), which will give a more accurate representation for precision (and likely L2 similarity).

2. Why does precision drop so much for 2bRAD-M as the biomass drops (Fig. 3b)? Since you see high recall, it means that you are capturing most of the ATCC MSA1002 species. But drop in precision, unless I interpret it incorrectly, indicates you are picking up unique, species specific markers from "unforeseen microbes". Is it a matter of thresholding on species detection or contamination of the mock community or other? A closer inspection of what you are picking up in the low biomass samples and discussion of the "unforeseen microbes" is important.

3. Potentially related, you observe a large inflation of detected species (16-23% more) inflation by 2B-RAD-M in the real stool sample as compared to mOTUs profiling in Fig. 4. How do you explain it? The highly abundant species by 2B-RAD-M but missing from mOTUs, are they present in the mOTUs database?

4. For the analysis of mock communities, what was the rationale to look at 5 microbe community under varying perturbation setting (Fig. 3a), and not ATCC MSA1002 that contains 20 microbes? Validation of 2bRAD-M on the ATCC MSA1002 in the varying host contamination scenario (95% - 99% host DNA) would be particularly valuable for proposing this method in the setting of terminal ileum or colon biopsies, where we expect a much more complex microbial community and a high host DNA contamination.

5. For the analysis of formalin fixed, paraffin embedded tissues (FFPE, from line 290 without ground truth and comparison to other profiling approach (e.g. 16S rRNA profiling) this section of the manuscript does not present as a part of a benchmark / validation but rather a biological exploration. As the authors write, sample preparation damages the DNA, introduces cross-linking, samples are of low microbial biomass. Could such conditions be reproduced by FFPE'ing a mock microbial community that is highly contaminated by host DNA? The current validation of a highly degraded DNA presented in Fig. 3a does not seem be a sufficient representation of the FFPE setup. Otherwise, could you benchmark against a set of samples pre- and post-FFPE? The final alternative would be to benchmark 2bRAD-M readout on FFPE samples against a 16S rRNA readout as in the section on environmental and skin samples.

Minor:

1. Please, provide a more detailed method section on the validation sections, i.e. how simulated or sequenced samples are mapped (e.g. alignment, classification, what method).

2. The choice of mapping against database of species unique tags as opposed to all tags or complete genomes is hard to question. But it would be interesting to see the increasing benefit (recall, precision, L2) of this approach as compared to all tags and complete genomes on simulated data or 5 / 20 species mock communities.

3. Lines 283-288 and 318-327. I think the strength of the manuscript is the method, its validation and applicability in various difficult settings. The attempt at a biological interpretation is distracting, does not add much value to the overall story and could be taken out.

4. In panel 4d, please rename y-axis from shotgun to WMS (or choose other consistent naming scheme).

**Authors Response**

**Point-by-point responses to the reviewers’ comments:**

Responses to Reviewer #1

In this manuscript, Sun et al. performed a comprehensive comparison of the 2bRAD-M method with whole-metagenome sequencing (WMS). The new method utilizes type IIB restriction endonucleases for sequencing of metagenomes and identifying microbial compositions. The tested method was previously introduced in 2012 for genotyping (Shi Wang et al., Nat. Methods 2012), and was optimized later on for NGS (Shi Wang et al. Nat. Protoc 2016), and generates short and identical length fragments (tags) that are used as genus- and species-specific markers. Sequencing of these markers is less costly than shotgun sequencing, while allowing for increased sensitivity that out-performs the 16s amplicon sequencing. The method proposes to tackle challenges related to I. samples with low amounts of DNA (LoA) II. Host DNA contamination (HoC) and III. High DNA degradation (HiD). The authors created a unique marker-specific database generated on ~170,000 bacteria, archaea and fungi genomes from the refseq database, by extracting the segments where the restriction enzymes would cut. Then they performed a computational simulation to validate the precision and recall of the proposed method and database in comparison to existing shotgun. They then performed a mock sample-based sequencing (on five bacterial genomes) and compared WMS, amplicon and 2bRAD-M sequencing in a 20-species known-composition community followed by validation of stool samples with WMS and low-biomass samples with amplicon sequencing. Finally, the authors performed 2bRAD-M on biopsies from cervical cancer preserved in paraffin and compared healthy, benign and malignant-sequenced microbiome compositions.

Overall, the 2bRAD-M method has the potential to elegantly improve the above challenges. However, there are major concerns in the current manuscript that must be addressed to reach an adequate stringency and reproducibility of the presented method.

*Response 2: We thank Reviewer #1 for the positive assessment on the relevance, robustness, and general interest of our work.*

Major issues:

1.	Host removal - While the method is proposed to tackle host contamination (HoC), and is shown to do so in controlled environments (a five-species bacterial community), the authors do not disclose how the method would perform under these circumstances. Importantly, the authors neither report on a step of host removal, which we assume is mandatory in this scenario, nor do they report the funnel of reads from raw reads -> host mapped reads -> bacteria mapped reads and percentage of unmapped reads, which are all vital in benchmarking this method. The authors are requested to:

a.	report how host removal was done, or perform host removal steps if it was not done.

*Response 3: Current approaches to deplete host reads can be conducted prior to DNA extraction or after DNA extraction. Pre-extraction approaches selectively lyse fragile mammalian cells and removes exposed DNA enzymatically, leaving only the intact microbial cells for downstream analysis. However, the multiple wash steps have hindered application of this method on low-biomass samples [1] and both loss and bias in bacterial identification were reported [2]. As for post-extraction approaches which target methylated nucleotides [3] or host-specific sequences for hybridization-based depletion with CRISPR/Cas9 [4], a significant impact and potential bias in bacterial detection was reported [5]. To avoid the bias introduced by host gene removal, we did not perform any depletion methods before or after DNA extraction. Actually, the bioinformatic pipeline can avoid the influence of host gene contamination, simply because there is little overlap between 2bRAD fragments originated from the human genome and from microbiome (for more details about the rationale, please refer to the following two responses).*

*To clarify this point, we have now inserted the following into Methods section (Line 387-389): “To avoid the bias introduced by human DNA removal, we did not perform any depletion methods before or after DNA extraction in this study.”*

b.	report the described reads funnel for each sequenced sample in order to allow for a fair comparison.

*Response 4: As advised, the funnel of “raw reads > host mapped reads > bacteria mapped reads and percentage of unmapped reads” was now supplemented (Table R1) in the revised manuscript to illustrate the read-utilization rate under high host DNA contamination situation. As we can see from this table, the 2bRAD reads originated from the five microbial species will be triple amplified under 90% and 99% host DNA contamination status. This is because the microbiome gene set is much larger than the human gene complement [6] (so does the Type IIB restriction enzyme sites), resulting an amplification of microbiome-originated reads after enzyme digestion. However, although larger genome size leads to more enzyme sites, the abundance estimation will not be influenced since the 2bRAD-M pipeline is a marker based taxonomic abundance profiler (please see our next response for the details).*

*Table R1 (Table S3 in the revised manuscript). The relative enrichment of 2bRAD reads originated from microbial species versus those originated from host in the high-host-contamination (HoC) group.*

c.	Provide motivation and explanation to why this method has the potential to overcome shotgun metagenomic ability to handle HoC data.

*Response 5: We attribute its rationality to the following three points. (i) Whole-metagenome shotgun sequencing (WMS) suffers from high host contamination, e.g., the microbiome-originated reads can be as low as 0.05% [7], while sequencing depth toward 1 million or 2 million reads is encouraged in WMS [8]. If 99% of reads are from human, then the sequencing depth should be 100~200 million reads, which is too expensive for large-scale microbiome studies. By sequencing the representative 1% of the human genome and microbial genomes, 2bRAD-M can dramatically reduce the cost and hence greatly increase the sequencing depth.*

*(ii) As we explained in Response #4 above, due to the highly imbalanced presentation of enzyme sites in microbial genomes and the human genome, microbial genomes can generate many more 2bRAD tags than the human genome. For example, if we set the 1:99 as the microbial DNA vs human DNA ratio in the mock samples, the sequencing reads ratio between them would be 3:97 in the real sequencing results. However, the abundance estimation of microbial species is NOT influenced since the 2bRAD-M use a DNA-to-Marker method for taxonomic profiling. For a typical DNA-to-Marker method, the abundance estimation of a species only depends on the read coverage and depth of a set of species-specific markers but has no association with how many reads (or 2bRAD tags) belong to this species [9].*

*(iii) The dominating host DNA in microbial samples not only inflates effective sequencing depth for microbial DNA, but also introduces high false positive (FP) in microbial identification, when using conventional WMS. However, the 2bRAD fragments in human genome are completely different from those in microbial genomes (as suggested by Fig. R1). Consequently, qualitative analyses such as abundance estimation for microbial species will be robust against host DNA interference, and the FPs would be greatly reduced.*

*Figure R1 (Fig. S10 in the revised manuscript). Very few 2bRAD fragments are shared across kingdoms. This in silico analysis was attempted to investigate how many 2bRAD fragments are shared across kingdoms or are uniquely identified in certain Kingdom. We collected the complete genomes in RefSeq (8588 virus genomes, 282 archaea genomes, 290 fungi genomes, and 12668 bacteria genomes), and applied BcgI as a representative 2bRAD enzyme to perform this analysis. Human shares almost no 2bRAD fragments with the microbes. Thus, the sequenced 2bRAD fragments from the human host will not interfere with the microbial identification.*

*To clarify this point, we have now inserted the rationale of 2bRAD-M for HoC in the Methods section, which reads (Line 467-477): “Rationale of 2bRAD-M in overcoming high host contamination We attribute the ability of 2bRAD-M to overcome high host contamination to the following. (i) By sequencing the representative 1% of the human genome and microbial genomes, 2bRAD-M can dramatically reduce the cost and hence greatly increase the sequencing depth. (ii) Due to the highly imbalanced presentation of enzyme sites in microbial genomes and the human genome, microbial genomes can generate many more 2bRAD tags than the human genome. For example, if we set the 1:99 as the microbial DNA vs human DNA ratio in the mock samples, the sequencing reads ratio would change to 3:97 in the actual sequencing results. (iii) The 2bRAD fragments in human genome are completely different from those in microbial genomes (Fig. S10). Consequently, qualitative analyses such as abundance estimation for microbial species are robust against host DNA interference, and the FPs would be greatly reduced.”*

2.	Computational simulations - several critical issues need to be addressed:

a.	Present simulation seems not to constitute a fair comparison, as the database was built in the exact manner as the simulated sequences were made, giving it an unfair advantage. As such, this approach does not shed additional corroborative light on the 2bRAD-M method, but solely on the database itself. To solve this inherent flaw, the tested method should be compared to the most similar database possible. We request the authors to use the ~170,000 that were used to create the 2bRAD-M database to build a database to be used by kraken/bracken and Centrifuge.

*Response 6: It was well known that the performance evaluation is impacted by the reference database used in the profilers [10]. Hence, to avoid the bias introduced by the database, a uniform database is recommended for benchmarking studies. We indeed attempted to customize the database with 173,165 microbial genomes (that were used in 2bRAD-M) for those best performing methods (especially MetaPhlAn2 and mOTUs2). But it is difficult to unify the databases. Firstly, although MetaPhlAn2 does provide the documents on the customizing MetaPhlAn2 database, it only allows users to add new genomes into existing database. This has led to many conflicted taxonomic annotations between MetaPhlAn2 and 2bRAD-M databases. Secondly, mOTUs2 also provides scripts to customize the marker database, which relies on a set of 40 universal single copy marker genes (MGs) for microbial identification or abundance estimation. According to the tutorial, we fetched mOTU markers for each of 173,165 microbial genomes using fetchMGs (https://github.com/motu-tool/fetchMGs). However, 157,395 of 173,165 microbial genomes in our database have no or fewer than 40 markers that are required by mOTUs2 for the taxonomic profiling. These genomes belong to 22,659 species which only account for 86.6% of all species (N= 26,163) in the 2bRAD-M database. This again leads to the inconsistent database composition between two tools. As a result, a perfectly fair comparison between 2bRAD-M and others by unifying database composition is unfortunately not practical.*

*Alternatively, to minimize such database bias in our benchmarking work, we have selected genomes from the intersection among the reference databases of MetaPhlAn2, mOTUs2, and Kraken2 (Bracken and Kraken2 use the same reference database) to generate the simulation sequencing data. This enables us to evaluate the performance of different profilers in an unbiased manner [9]. To better compare the performance, we have increased the sample size of simulation data for evaluation in the revised manuscript. Specifically, we expanded the original five simulation data to a 25-sample simulation dataset [9] that were generated using an identical method. The benchmarking results still support an outstanding performance of 2bRAD-M, as shown in Figure R2 below.*

*Figure R2 (Figure 2d in the revised manuscript). Performance comparison of 2bRAD-M with Kraken2, Bracken, MetaPhlAn2 and mOTUs2 based on 25 simulated communities. Two types of abundance are used as the ground truth of the simulation data to evaluate the performance: sequence abundance is used to evaluate Bracken and Kraken2, while taxonomic abundance is used to evaluate 2bRAD-M, mOTUs2, and MetaPlAn2.*

*Accordingly, we have now inserted below into Results (Line 131-142): “The accuracy and computational efficiency of 2bRAD-M was assessed by comparison with highly-cited WMS profiling tools such as Kraken2 [18], Bracken [19], mOTUs2 [20] and MetaPhlAn2 [21], using the in silico simulated data of 25 microbial communities for benchmarking (2bRAD-M applied BcgI-derived species-unique markers to profile the simulated data, while the others produced the abundance profiles from simulated pair-end 150-bp WMS reads; Methods). In terms of average Shannon similarity, Bray-Curtis similarity, L2 similarity (accuracy metrics evaluated based on the ground truth), precision and recall, 2bRAD-M showed a level of 0.998, 0.97, 0.98, 0.89, 0.98 respectively, which either outperformed or are equivalent to others (Fig. 2b).”*

b.	There is no description in the methods to which databases kraken, bracken and Centrifuge were applied to and which parameters were used (neither for Metaphlan, but a pre-built database exist in this scenario). We ask the authors to describe the full exact nature in which this simulation was performed.

*Response 7: As advised, we have now updated the Methods section to include detailed description of how we applied taxonomic profilers and how we simulated metagenome data for benchmarking, as shown below.*

*“Usage of metagenomic profilers For shotgun metagenomic sequencing data, we validated the taxonomic profiles with state-of-the-arts profilers, such as Kraken2 [18], Bracken [19], mOTUs2 [20], MetaPhlAn2 [21] and Centrifuge [22]. The detailed procedures are listed below.*

*Kraken2 (v2.0.8-beta) is a k-mers based taxonomic classification method. It searches for 35bp k-mers from the query sequence in a precomputed database that matches k-mers to the lowest common ancestor (LCA) taxon of all genomes that contain that taxon. The default database was constructed using complete bacterial and viral genomes from NCBI RefSeq (2019 Oct). A filtering abundance threshold of 0.01 (default) was selected. The Kraken2 command below was used: “kraken2 --threads 32 --fastq-input --gzip-compressed --paired input_1.fastq.gz input_2.fastq.gz –output output.reads --report output.report”.*

*Bracken (v2.5) utilizes the read classification output from standard Kraken for a Bayesian re-estimation of taxonomic abundances, which significantly improves the false-positive issue of standard Kraken and implicitly normalizes for genome length. The kraken-filter was used to filter raw classifications at the 0.01 threshold. The Bracken command of “est_abundance.py -i input -k db -o output” was used.*

*MetaPhlAn2 (2.96.1) is a marker-gene alignment approach that relies on a precomputed databases containing clade-specific marker genes. Query reads are aligned via bowtie2 to the marker genes for microbial identification and abundance estimation. The database version used is mpa v296 CHOCOPhlAn 201901. The MetaPhlAn2 command of “metaphlan2.py input_1.fastq.gz, input_2.fastq.gz --input_type fastq --nproc threads --bowtie2out output_bowtie2out.txt -o output.report” was used.*

*mOTUs2 (v2.5.1, database version 2.5.0) is a marker-based method that compiles a large variety of phylogenetic marker genes from multiple biomes. Query reads are aligned using bwa mem and further processed to generate an abundance profile. The mOTUs2 command of “motus profile -f input_1.fastq.gz -r input_2.fastq.gz -o output.report -t threads” was used.*

*Centrifuge (v1.0.3-beta) is a microbial classification engine based on FM-index which enables rapid, accurate, and sensitive taxonomic labeling of short reads. To analyze 2bRAD sequencing data of MOCK CAS (Fig. 3a), we employed Centrifuge to perform the 2bRAD-tag-level taxonomic classification that none of other existing profilers can handle. We started by customizing a database for Centrifuge that only contains genomes of the five species in MOCK CAS. Next, Centrifuge was used as a search engine to calculate the 2bRAD read-level proportion of five microbial species. Accordingly, we repeated this process using WMS for benchmarking purpose.”*

*Moreover, as explained in our Response #6 above, we generated a 25-sample simulation metagenome dataset [9] based on the same method described in our previous paper. “We first simulated 25 abundance profiles representing microbiota from distinct habitats (gut, oral, skin, vagina, and building, with five profiles for each) with known abundance profiles (abundance was created randomly from a log-normal distribution using the function rlnorm in the R language with the following parameters: meanlog = 0 and sdlog = 1). Given a specified species composition (taxonomic abundance), their sequence abundance can be inferred accordingly (taxonomic abundance equals sequence abundance divided by their genome length), and then Wgsim (https://github.com/lh3/wgsim) was used (with default parameters) to simulate the sequences. The intersection of organisms in the mOTU2, MetaPhlaAn2 and Kraken2 reference databases was used as the source genomes for the simulation. The simulation scripts for metagenomic data can be found in https://github.com/shihuang047/re-benchmarking.”*

*All these details now have been added in Methods (Line 501-532, and Line 491-500). For more information about the difference between “taxonomic abundance” and “sequence abundance”, please refer to Response #11 below.*

c.	It is not clear how kraken and Centrifuge had such different results as they are practically algorithmically identical.

*Response 8: The poor performance of Centrifuge was also reported in a previous benchmark study for metagenome taxonomic profilers. The reason is that “Centrifuge’s default database is significantly lossily compressed (throwing away database sequences to save space), which also results in poor recall.” [11] However, its performance can be greatly improved by customizing the database with complete genomes from NCBI RefSeq as mentioned in the same study. To avoid any potential confusion and misleading, we decided to remove Centrifuge from the comparison due to its default database issue (Figure R2).*

*In Figure 3a, to validate the 2bRAD-M’s performance and minimize the performance bias introduced by bioinformatic pipelines, we employed Centrifuge to map both WMS (150-bp) and 2bRAD-M (32-bp) generated reads to the reference microbial genomes for sequence abundance estimation for MOCK CAS. Specifically, we replaced the Centrifuge’s default database with a customized database comprising of only genomes of those five species in the MOCK CAS. Moreover, the taxonomic profiling results generated by Centrifuge were not directly used, instead we only employed Centrifuge as a search engine (essentially bowtie2) to estimate the sequence proportion of the five microbial species. Centrifuge was deliberately used in this analysis due to its unique features. To our knowledge, Centrifuge is the only metagenomic profiler that provides alignment results for extremely short reads (as short as ~30 bp), while it can also handle regular Illumina reads. Other main-stream k-mers based classification methods e.g., Kraken2 are not able to generate read-level annotation for 2bRAD fragments.*

3.	Fecal sample sequencing - There are major data inconsistencies in the data provided in table S3 and figure 4, and missing information related to shotgun sequenced samples.

a.	In S3, Eubacterium rectale is has 0.41% relative abundance and ranked 178 in the shotgun sequenced method, while there are bacteria with lower abundance that ranked higher. Please clarify this issue.

*Response 9: We apologize for this typo, which is corrected. It should be “17” instead of “178” in Table S3.*

b.	In figure 4d-f, it seems that there are only a few bacteria that had high relative abundance and were not detected by 2bRAD-M, while the tables suggest otherwise. Please clarify this issue.

*Response 10: We are sorry for the misleading log-scale transformation here. The main issue is that X-axis and Y-axis are all ln (loge)-transformed relative abundance, i.e., loge (relative abundance). Now in the scatter plots both X-axis and Y-axis are in log10-scaled relative abundance, as shown in the revised figure panels in Response #11 below.*

c.	It is not clear which database was used in this section and which method. In order to provide the proper comparison, with the same exact taxa, we request you use the kraken/bracken/Centrifuge methods, built using the same database that use used for the 2bRAD-M database.

*Response 11: We agree that taxonomic profiling result of WMS data is heavily influenced by the metagenomic profiler and the database used.*

*For the issue of selecting taxonomic profiler for WMS data, as suggested, we did carefully consider the proper way of comparison between 2bRAD-M and other state-of-the-arts. However, for the various metagenomic profilers (mOTUs2, MetaPhlAn2, Kraken, Bracken, Centrifuge, etc.), their performance on WMS data is highly variable, due to algorithm difference or even different output data types. In the end, we chose mOTUs2 as the most suitable metagenomic profiler for WMS data (original Figure 4) and benchmarking our 2bRAD-M, but not Kraken/Bracken/Centrifuge, for two reasons. (i) We selected the profiler/method based on the updated knowledge of metagenomic profilers from our recent study on Nature Methods [9]. In that study, we systemically benchmarked the performance of mOTUs2, MetaPhlAn2, Kraken, and Bracken on the same simulation metagenome samples. Overall, we found that outputs from metagenomic profilers in different categories can be totally incomparable as they represent two fundamentally different types of abundance data. Specifically, some tools were designed to report relative sequence abundance while others report relative taxonomic abundance [9]. For example, DNA-to-DNA methods (e.g., Kraken and Bracken) output the relative abundance of a given taxon calculated as the proportion of sequences assigned to it out of the total number of sequences—that is, sequence abundance. By contrast, DNA-to-marker methods (e.g., mOTUs2 and MetaPhlAn2) directly output the relative abundance of each taxon calculated as the number of genomes of that taxon relative to the total number of genomes detected—that is, taxonomic abundance. Most importantly, these two types of abundance are not possible to transform from one to another for a real sequencing sample. (ii) 2bRAD-M is a DNA-to-Marker method. To avoid introducing any bioinformatic bias here, DNA-to-Marker profilers such as mOTUs2, MetaPhlAn2 were selected to analyze WMS data and validated 2bRAD-M from the same biological samples, not DNA-to-DNA methods (e.g., Kraken and Bracken). Notably, mOTUs2 is a typical DNA-to-Marker method that performs best in this category in the previous benchmarking work (such as the CAMI challenge [12, 13]), and it includes an NCBI RefSeq database that is close to what we are using here.*

*As for the database issue, it is still technically difficult to customize a reference database for mOTUs2 or MetaPhlAn2 that have the same set of species-level taxa as 2bRAD-M. When mOTUs2 was selected, we understand that there is still a discrepancy in the database contents between 2bRAD-M and mOTUs2. As we explained in Response #6 above, we did attempt to customize the database with 173,165 microbial genomes (that were used in 2bRAD-M) for those two best performing DNA-to-Marker methods (MetaPhlAn2 and mOTUs2). However, it is not practical. Firstly, although MetaPhlAn2 does provide documents on customizing database, MetaPhlAn2 only allows users to add new genomes into existing database. This results to many conflicted taxonomic annotations between MetaPhlAn2 and 2bRAD-M databases. Secondly, mOTUs2 relies on a set of 40 universal single copy marker genes (MGs) for microbial identification or abundance estimation. According to its tutorial, we fetched mOTU markers for each of 173,165 microbial genomes using fetchMGs (https://github.com/motu-tool/fetchMGs). However, 157,395 of 173,165 microbial genomes in our database has no or fewer than 40 markers that required by mOTUs2 for the taxonomic profiling. These genomes belong to 22,659 species which only account for 86.6% species (N= 26,163) in the 2bRAD-M database. This will again lead to the inconsistent database composition between two tools. Thus, a perfectly fair comparison in terms of database is not practical here.*

*Therefore, we have proposed an alternative approach for fair comparison of taxonomic profiling performance between 2bRAD-M sequencing and direct WMS without introducing any bias from bioinformatic tools. From above tests, we learned that it is not feasible to unify the marker database composition between bioinformatic tools aforementioned. Even we can do so, the bias introduced by the algorithm difference or effectiveness of marker gene sets between bioinformatic tools remains inevitable. Therefore, we attempted to fetch the 2bRAD reads from the WMS data and then performed the standard 2bRAD-M pipeline for the WMS-originated 2bRAD reads and real 2bRAD sequencing data. By comparing the difference between the profiling results of WMS-originated 2bRAD reads and real 2bRAD sequencing data, we can fairly evaluate 2bRAD-M performance with WMS, preventing introducing noise from the different profilers and databases (as shown in Figure R3 below). As shown in this figure, a linear relationship was found between WMS and 2bRAD-M abundance data, with an average Pearson correlation coefficient of 0.99. Moreover, the correlation of alpha diversity (Shannon index) between these two data types as well as L2 similarity and BC similarity of the profiling results are consistently high. These results support the performance of 2bRAD-M in taxonomic profiling by only using 1% of the WMS sequencing data.*

*Figure R3 (Figure 4d, 4e, 4f in the revised manuscript). Comparison of taxonomic profiles at the species level between 2bRAD-M and WMS. To perform the comparison in a fair manner, we extracted 2bRAD reads from WMS data and then used these WMS-originated 2bRAD reads as input for the 2bRAD-M computational pipeline. Then species abundance generated by 2bRAD-M (using 2bRAD-M sequencing data) is used as X-axis while the species abundance generated by 2bRAD-M (using WMS data) as Y-axis for the scatter plot.*

*Accordingly, we have inserted the following into Results (Line 212-218), which reads: “As for the species level, to perform the comparison in a fair manner, we extracted 2bRAD reads from WMS data and then used these WMS-originated 2bRAD reads as input for the 2bRAD-M computational pipeline, then we found that WMS-originated 2bRAD reads and real 2bRAD sequencing data are also concordant in species level profiling results, as evident by a high Pearson correlation (R=0.99) and high L2 similarity (up to 97.8%). Moreover, only 0.40% of the taxa in WMS were not identified in 2bRAD-M (Table S5). Thus 2bRAD-M can produce highly complete and accurate species-level profiles that are equivalent to WMS.”*

d.	Please provide detailed information regarding the bacteria that were only detected in the shotgun method.

*Response 12: As advised, we have now summarized the proportions (as the Venn diagram shown in Figure R3) of bacteria that were only identified by WMS data or 2bRAD-M data (Table R2) in a supplementary table, which clearly showed the difference in the microbial identification between these two data types. Notably, 13, 23, and 22 species are only found in WMS-originated 2bRAD data of the three fecal samples, and 6, 6, and 5 species only found in the actual 2bRAD data. These uniquely identified species by WMS data or 2bRAD-M have an extremely low relative abundance (averagely 0.01%) which is usually close to or under the filtering threshold and would generally be discarded in downstream statistical analysis. These indicate a high consistency of taxonomic profiling results between 2bRAD-M data for WMS data.*

*Table R2 (Table S5 in the revised manuscript). The relative abundance of microbial species that are uniquely detected in the WMS or 2bRAD-M data of fecal samples. These species account for a very small proportion (<0.5%) in the fecal microbiota.*

4.	2bRAD-M database - While the generation of a specific database for the 2bRAD-M is indeed useful, it is not explored enough to understand its validity, specifically. Please disclose the following:

a.	Distribution of the amount of tags for each species/genus/phylum.

*Response 13: As advised, we have now analyzed the distribution of 2bRAD tags that theoretically existent in all microbial genomes (N=173,165) and illustrated the distribution at each taxonomy level (Figure R4). Regardless of the Type IIB restriction enzyme used, all 26,163 microbial species in the database possess a certain amount of 2bRAD tags. Moreover, 99.8% (digested by BplI) to 100% (digested by BsaXI, BslFI, CjeI, CjePI, HaeIV, and Hin4I) species contain more than 10 2bRAD tags, and 92.4% (digested by PpiI) to 99.9% (digested by CjeI, CjePI, HaeIV, and Hin4I) species contain more than 100 2bRAD tags. The average 2bRAD tag number within a microbial species ranges from 500 (CspCI) to 9,066 (CjePI), which is mainly dependent on the Type IIB restriction enzyme used. For example, for BcgI (the Type IIB restriction enzyme used in our manuscript), all 26,163 microbial species contain BcgI enzyme sites, and among them, 26,156 species (99.9%) contain more than 10 2bRAD tags, 25,835 species (98.7%) contain over 100 2bRAD tags, and 21,588 species (82.4%%) contain over 1000 2bRAD tags. As for the upper taxonomy level (such as genus), the amount of 2bRAD tags for each taxon is much larger than the species level, as we have demonstrated in Figure R4.*

*We want to emphasize that although in silico analysis revealed that BcgI is not the only top choice for microbiome sequencing, it is commercially available. Therefore, our future work will evaluate additional Type IIB restriction enzymes such as CjePI and Hin4I for 2bRAD-M sequencing.*

*Figure R4 (Figure S1b in the revised manuscript). Distribution of theoretically existent 2bRAD tags on different taxonomy levels. The 2bRAD tags were first generated by in silico digestion of 173,165 microbial genomes, and then non-redundantly merged based on their taxonomy annotation. The 2bRAD tags generated by the different enzymes were then compared at various taxonomy levels. The Type IIB restriction enzymes in X-axis are ordered by alphabet.*

*Accordingly, we have inserted the distribution of 2bRAD tags in Results section, which reads (Line 116-120): “Notably, 2bRAD tags are detected in all genomes regardless of the Type IIB restriction enzyme used (Fig. S1b). Moreover, the number of 2bRAD tags within a genome and their GC content are highly consistent with the length and the GC% of genomes (r>0.98) (Fig. S2) for all the 16 Type IIB restriction enzymes, suggesting unbiased and broadly applicable representation of the microbial genomes by these tags.”*

b.	Distribution of unique amount of tags for each species/genus/ phylum to detect under- and over-represented bacterial genomes.

*Response 14: As advised, we have now analyzed the distribution of unique 2bRAD tags that can be used to build the reference database and displayed the distribution on each taxonomy level (Figure R5). We found that unique 2bRAD tags can be detected in most species, from 88.1% (digested by PsrI) to 95.1% (digested by CjePI). Moreover, 81.0% (digested by PsrI) to 88.7% (digested by CjePI) species contain over 10 unique 2bRAD tags, and 56.2% (digested by PsrI) to 83.1% (digested by CjePI) species contain over 100 unique 2bRAD tags. The average number of unique 2bRAD tags ranges from 219 (PsrI) to 4,671 (CjePI), depending on the Type IIB restriction enzyme used. Taking BcgI as an example again, we found that 24,406 microbial species contain BcgI enzyme sites, and among them, 22,552 species (86.2%) contain over 10 unique 2bRAD tags, and 20,234 species (77.3%) contain over 100 unique 2bRAD tags. Similar to the distribution of theoretically existent 2bRAD tags, the number of unique 2bRAD tags at upper taxonomy levels (such as genus) is much larger than that at the species level (Figure R5).*

*The main possible cause of missing unique 2bRAD tags in some species is the problematic taxonomy annotation in RefSeq. For example, over one third of the NCBI-classified representative microbial genomes would have a different species-level taxonomy if they use average nucleotide identity as criteria to set taxonomic boundaries between species [14]. The problematic annotations on the species structure introduce noise to the selection of unique 2bRAD tags, and this is a potential motivation for us to choose GTDB as the genome source in our future releases (99.99% of microbial species in GTDB contain more than 10 unique 2b tags). Notably, despite the limitation that some species aren’t identified due to the missing of unique 2bRAD tags, 2bRAD-M (e.g., BcgI as enzyme) is still the method with the largest number of identifiable species among existing DNA-to-Marker metagenomic profilers (as compared to 7,700 of mOTUs2 [15], 7,500 of MetaPhlAn2 [16], and 13,500 of MetaPhlAn3 [17]).*

*Figure R5 (Figure S1a in the revised manuscript). Distribution of unique 2bRAD tags at various taxonomy levels. The 2bRAD tags were first generated by in silico digestion of 173,165 microbial genomes, and then non-redundantly merged based on their taxonomy annotation. We selected those 2bRAD tags that are not duplicated between any two taxa and named them as unique 2bRAD tags. The numbers of unique 2bRAD tags generated by different enzymes are shown at various taxonomy levels. The Type IIB restriction enzymes in X-axis are ordered by alphabet.*

*Accordingly, to highlight this issue, we have now modified below in Results section, which reads (Line 112-114): “Besides BcgI, species-specific 2bRAD tags (unique 2bRAD tags) were also identified for each of the other 15 Type IIB enzymes (AlfI, AloI, BaeI, BplI, BsaXI, BslFI, Bsp24I, CjeI, CjePI, CspCI, FalI, HaeIV, Hin4I, PpiI and PsrI; Table S1, Fig. S1a) to establish the usability of all Type IIB enzymes for 2bRAD-M.”*

Minor issues:

1.	Metrics - while the usage of l2 similarity is not discouraged, please include alpha- and beta-diversity when handling biological and mock samples, as these are commonly used.

*Response 15: We agree that although L2 similarity was used in the previous benchmarking work, it has not been commonly used in microbiome studies. Thus, we added the evaluation of alpha diversity (Shannon index) and beta diversity (Bray-Curtis similarity) in the benchmarking of 2bRAD-M. For example, we have now added these measurements in our new Figure 2 (please see our Response #6), Figure 3 (Response #17), Figure 4 (Response #11), and Figure S5. Notably, the comparisons based on alpha and beta diversity also support the excellent performance of 2bRAD-M in microbial species identification.*

2.	It is mentioned in the introduction that the amount of DNA needed for sequencing is at least 20ng, while it is common knowledge that even 1ng is enough for shotgun, and even less is sufficient for 16s. please correct.

*Response 16: Regarding the minimum amount of DNA for shotgun metagenomic sequencing, most suggestions are made in the context of fecal samples, where most DNA are derived from microbes. For other sample types (such as low-microbial-biomass samples), it can be more difficult to obtain high-quality sequencing results via the same amount of “minimal DNA”. Based on our knowledge, higher starting DNA material (20 ng) is usually desirable or even necessary and will certainly benefit downstream analysis.*

*As advised, we have now revised the sentence as (Line 49-52): “However, although 1ng is applicable via certain specialized kits [4], WMS usually requires a high amount of DNA as the starting material (≥50 ng preferred, 20 ng at a minimum), and moreover is not efficient in tackling DNA samples that are low in biomass, heavily degraded or dominated by host DNA [1, 5]”.*

3.	Why weren't all the WMS tools tested against the 20-bacteria mock-community? Please add a comparison for all tools.

*Response 17: As advised, we have now supplemented profiling results generated by state-of-the-art metagenomic profilers, e.g., MetaPhlAn2, mOTUs2, Bracken, and Kraken2 in our revised manuscript (Figure R6). In the original version, we only demonstrated the abundance of the species within the 20 species in MOCK MSA 1002. To better illustrate the performance of different methods, we have now added 2bRAD-M profiling results of MSA 1002 with host DNA contamination ranging from 90% to 99% (please see our Response #30), and we included false positive identifications (as white blank in the bar plots in Figure R6) in the revised manuscript. As expected, 2bRAD-M still offers outstanding performance in decoding the mock community, which is comparable with MetaPlAn2 which shows the best performance among all the WMS profilers used. We have revised the manuscript accordingly, now it reads (Line 181-201):*

*“Samples with total DNA amount ranging from 10 ng, 1 ng, 100 pg, 10 pg, 1 pg, and DNA host contamination from 90%, 95% and 99% were prepared from this mock community for 2bRAD-M profiling. As a reference control, a 100-ng sample was profiled with the 16S-rRNA and WMS approaches (the 1pg ~10ng samples failed). The precision for 2bRAD-M is 90.9%, 76.0%, 46.3%, 40.0%, 14.0%, 82.6%, 79.2%, 90.5% for the 10 ng, 1 ng, 100 pg, 10 pg, 1pg, 90% host DNA, 95% host DNA, and 99% host DNA samples respectively. This is in contrast to the 53.7% (average number of different profilers) for WMS-100ng and the 17.3% for 16S-rRNA-100ng (Fig. 3b). Therefore, 2bRAD-M offers much lower false positives in detecting the species than 16S-rRNA and WMS. As for recall, 2bRAD-M is 100.0%, 95.0%, 95.0%, 80.0%, 75.0%, and 95% for 10 ng, 1 ng, 100 pg, 10 pg, 1 pg, and host-DNA contaminated samples respectively, as compared to the 93.7% for WMS-100ng and the 90.0% for 16S-rRNA-100ng. Therefore, 2bRAD-M offers a comparable level of sensitivity to WMS and 16S-rRNA when starting with down to 100 pg of DNA. Moreover, the L2 similarity for 2bRAD-M is 91.0%, 89.1%, 88.5%, 84.8%, 48.5%, 91.8%, 89.4%, and 67.7% for 10 ng, 1 ng, 0.1 ng, 10 pg, 1pg, 90% host DNA, 95% host DNA, and 99% host DNA samples respectively, as compared to 82.1% for WMS-100ng and 70.0% for 16S-rRNA-100ng. As for alpha diversity (Shannon index) and beta diversity (Bray-Curtis similarity), profiling results of the low biomass samples by 2bRAD-M are comparable to the corresponding high biomass samples by WMS and 16S as well (alpha diversity: averagely 92.5% by 2bRAD-M, 97.4% by WMS, 95.5% by 16S; beta diversity: averagely 73.8% by 2bRAD-M, by 74.6% WMS, 60.6% by 16S). Taken as a whole, these results demonstrated 2bRAD-M’s ability to profile complex microbiota with a high level of sensitivity and specificity.”*

*Figure R6 (Figure 3b in the revised manuscript). Benchmarking 2bRAD-M against conventional metagenomic approaches using the mock community of MSA 1002. Each stacked bar plot in the left panel shows the resulting taxonomic profile from a benchmarked method in a library-preparation or bioinformatic setting. The white blank refers to false positive identifications. For WMS data, we employed various bioinformatic tools (e.g., MetaPhlAn2, Bracken, Kraken2, and mOTUs2) to generate taxonomic profiles on MSA 1002. For 2bRAD-M data, we applied our own bioinformatic pipeline to generate the taxonomic profiles of MSA 1002 with low to high DNA amounts, and under various degrees of host DNA contamination. In the right panel, bars in each row indicate the corresponding precision, recall, L2 similarity, Bray-Curtis dissimilarity and Shannon index similarity of the predicted taxonomic profile as compared to the ground truth. The bars in the “Shannon” column represent the correlation between Shannon index of the profiling results and the ground truth (see Methods).*

4.	Other tags are mentioned but not practically tested. What would be expected if a few tags would be used in a combined manner? Wouldn't such an approach increase specificity?

*Response 18: We agree that the resource of 2bRAD marker tags is imperative for microbial species identification and abundance estimation. Each Type IIB restriction enzymes can produce a highly specific set of 2bRAD tags to other Type IIB restriction enzymes for the same microbial genome. These sets of 2bRAD tags vary greatly in number and thus in genome coverage. Usually, for a Type IIB restriction enzyme used, the more 2bRAD tags produced for each genome, the more accurate taxonomic profiling performance will be observed. The combined use of 2bRAD tags from 2 to 16 Type IIB restriction enzymes can theoretically increase the profiling performance (such as specificity) in certain circumstances. Due to limited availability of all the 16 Type IIB restriction enzymes in the lab, we didn’t have a chance to systematic evaluate the performance gain using a combined set in the 2bRAD sequencing on real samples. However, we did this with a simulated 50-species metagenomic data (shown in Figure 2) in an in silico analysis. Specifically, we produced 2b-Tag-DB for each of 16 Type IIB restriction enzymes for taxonomic profiling on this sample as well as the 2b-Tag-DB for a combined set of 16 Type IIB restriction enzymes. We firstly benchmarked the taxonomic performance of each type IIB restriction enzyme against the combined set. Next, we explored the relationship between the tag number or genome coverage of a type IIB restriction enzyme and taxonomic profiling performance (L2 similarity or Pearson correlation; Figure R7). The main conclusions are below.*

*(i) Combining tags from all the 16 types IIB restriction enzymes slightly outperformed some individual Type IIB restriction enzymes alone, e.g., PsrI and PpiI in terms of Pearson correlation. However, these IIB enzymes (PsrI and PpiI) only produce 2bRAD fragments represented lower than 1% original microbial genomes. (ii) Combining all Type IIB restriction enzymes didn’t necessarily produce significantly better performance than other individual Type IIB restriction enzymes in this simulation. For instance, performance of Hin4I alone and CjePI alone is almost identical to that of the combined set in both L2 similarity and Pearson correlation. (iii) Individual Type IIB restriction enzyme that can represent over 1.99% of genome, e.g., BcgI (the one we used in our study), performs sufficiently well as compared to the combined set.*

*On the other hand, the process of digestion using multiple Type IIB restriction enzymes on real samples will be limited by the low amount and quality DNA (e.g., trace DNA) in low microbial biomass samples, and multi-digestion also significantly increase the DNA library preparation time. Since little performance gain was observed by the combined set in the simulation, we are not recommending applying multiple enzymes for the real samples. In fact, a single Type IIB restriction enzyme such as BcgI shall meet the requirement for decoding microbial taxonomic profiles with a high accuracy in a cost-effective manner.*

*Figure R7 (Figure S3 in the revised manuscript). Comparison of the profiling performance between individual Type IIB restriction enzymes and a combined set of them. The bar plot shows the taxonomic profiling performance (L2 similarity and Pearson correlation) of the 2bRAD marker set from one of the 16 Type IIB restriction enzymes or a combined set. The X-axis shows a Type IIB restriction enzyme and the percentage of original microbial genomic content that corresponding 2bRAD fragments can represent, whereas Y-axis shows the profiling performance. The yellow bars represent the L2 similarity between predicted and ground-truth abundances, and the green bars refers to the Pearson correlation between them. The combined marker set from 16 Type IIB restriction enzymes does not significantly improve performance in abundance estimation as compared to that from individual Type IIB restriction enzymes.*

5.	As mentioned above, it is unclear which database reference was used from only the citation of the shallow metagenomic paper. A reference should be built for both kraken/bracken and Centrifuge from the refseq (the same exact genomes that were used for the tag db) for a complete and fair comparison.

*Response 19: Since 2bRAD-M is a DNA-to-Marker metagenomic profiler, it is inappropriate to use the sequence abundance generated by DNA-to-DNA metagenomic profilers (e.g., Bracken, Kraken2, and Centrifuge) for benchmarking. However, the two best-performing DNA-to-Marker metagenomic profilers do not support customization of reference database well [11]. Thus, we fetched the 2bRAD reads from WMS data and performed the profiling using the standard 2bRAD-M computational pipeline to avoid the influence from the bioinformatic process. By comparing the profiling results for WMS-originated 2bRAD reads and real 2bRAD-M sequencing data, we finally have a fair strategy to compare the two data types. The results still support the outstanding consistency of 2bRAD-M and WMS data. For additional details, please refer to Response #11 above.*

6.	On what data was the Pearson correlation calculated? Does it include bacteria that weren't discovered? It seems that it doesn't take them into account, and it should contain them, or properly explained in the text if it does not.

*Response 20: We have indeed included all the microbial species into account when calculating the Pearson correlation in our original Fig. 2, Fig. 3, Fig. 4 and Fig. S3. Specifically, for those unique species that is identified only in one method (or is in the ground truth that not identified by other methods), we replaced NA with 0 as their abundance in the Pearson correlation calculation. If we only focus on the “shared” microbial composition, the Pearson correlation could have even higher than the current ones. The extremely high Pearson coefficients indicate outstanding stability, repeatability, and consistency of 2bRAD-M.*

*As advised, to avoid any confusion, we have inserted below into Methods section (Line 566-567): “In addition, in Pearson correlation analysis, for those unique species that are identified only in one method, we replaced their abundance with 0 in calculating the Pearson correlation coefficient.”*

7.	It seems that the rarefaction curves in S2 are not in conjunction with the text. In the Shannon diversity, the samples are not close to the alpha diversity of the full length, while the species richness does not reach a stratification (neither for WMS). Could OTUs be calculated following filtration of extremely low abundant species?

*Response 21: After checking the script for random subsampling in the original Figure S2, we found that an inappropriate strategy (sampling with replacement) was used in this process (instead, sampling without replacement is more suitable for such subsampling process). We have now fixed this subsampling issue and generated new profiling results (Figure R8), which fully support our statements that the robustness of 2bRAD-M outperforms WMS. To filter those extremely low-abundance species identified, we have used 0.001% abundance threshold for WMS data and a G score of 10 for 2bRAD sequencing data.*

*Figure R8 (Figure S4 in the revised manuscript). Rarefaction analysis reveals the desirable sequencing depth for 2bRAD-M and WMS for reliable taxonomic profiling. In each scatter plot, we compared the profiling results of a fecal sample based on a method (either 2bRAD-M or WMS) at deep or shallow (by subsampling) depth of sequencing, via Shannon diversity, beta diversity (on the Bray-Curtis similarity), and species richness. Based on the Shannon diversity and Bray-Curtis similarity, profiling performance of 2bRAD-M quickly saturates at a shallow sequencing depth (2-3 million reads per sample). In contrast, for the same metrics, WMS-based taxonomic profiles saturate at a far deeper sequencing depth (about 50 million reads per sample), suggesting much higher sequencing costs. The microbial richness (number of species-level taxa detected) based on both methods still grows as the sequencing depth increases, which is consistent with current knowledge on metagenomic diversity analysis.*

8.	The scales of the rarefaction curves are unfairly plotted against the shotgun sequencing method. As the diversity measures are measured with respect to 400M reads or 15M reads. In order to provide a fair comparison, 15M samples should be sequenced and calculated or subsampled for 15M reads.

*Response 22: For a fair comparison between 2bRAD-M and WMS, we first performed in silico analysis to explore the quantitative correlation between WMS data and 2bRAD-M data. Specifically, 2bRAD tags were computationally fetched from the ultra-deep WMS data. From averagely ~15 reads in the WMS data, one 2bRAD tag (digested by BcgI) can be identified and fetched, suggesting the read number ratio between 2bRAD-M and WMS data is roughly 1:15. Considering this intrinsic difference in the data volume (reads ratio) between the two data types, it is not fair to compare their performance at the same sequencing depth (e.g., 15M vs 15M). Therefore, we compared the rarefaction curves with different scales of sequencing depth (X-axis), that is, setting a maximum value as 13.5M for 2bRAD-M while 200M for WMS on the X-axis) (Figure R8, which is shown above). As we can see from this figure, the Shannon diversity, BC similarity and L2 similarity hit a plateau with only 2-3M reads of 2bRAD-M while WMS still needs about 50M reads to reach the same level.*

*Accordingly, to highlight this issue, we have now modified below in Results section, which reads (Line 219-224): “To test how it influences 2bRAD-M performance, the fecal sequencing datasets were subsampled to various depths for 2bRAD-M (average 13.5 million reads per sample, subsampled at 0.1, 1, 2, 3, 5 to 13.5 million sequences per sample) or WMS (subsampled at 0.5, 10, 20, 30, 40, 50, 100, and 200 million sequences per sample since one 2b read can be identified and fetched from about 15 WMS reads) respectively.”*

9.	The dendrograms for the hierarchical clustering in figures 5a-b should be provided and explained

*Response 23: As advised, we have now added the dendrograms for the hierarchical clustering in Figure 5a and 5b. We first showed that unsupervised clustering at the genus level uncovered two distinct clusters among the 20 underarm samples, with one cluster featuring higher relative abundance of Staphylococcus observed in both 16S rRNA and 2bRAD-M. Then, the species-level profile from 2bRAD-M readily revealed that the observed difference in Staphylococcus was in fact underlaid by the higher relative abundance of S. epidermidis, with no significant change in other Staphylococcus spp. between the clusters (Figure R9). Thus, the ability of 2bRAD-M to resolve such species-level heterogeneity (e.g., Staphylococcus spp.) in low-biomass samples can potentially enable novel biological insights.*

*Figure R9. 2bRAD-M analysis of low biomass samples collected from underarm. (a) Relative abundance of bacteria at genus-level (>1%) identified in underarm by 16S rRNA sequencing (left panel) and 2bRAD-M (right panel) with the samples stratified by cluster group. (b) Relative abundance of bacteria at species-level taxa (>1%) identified in the underarm by 2bRAD-M with the samples stratified by cluster group.*

*We agree with Reivewer2’s comment (Response #34) that the attempt at a biological interpretation might be distracting and does not add much value to the overall story. Thus, we decided to remove the biological interpretation of underarm profiling results (original Figure 5a, 5b) and focus on the method itself.*

10.	was species resolution demonstrated for 16s? It is claimed that the signal of s. epidermidis wouldn't be seen, but it might be captured by 16s.

*Response 24: We have indeed attempted to classify 16S rRNA sequencing reads at the species level. Specifically, we employed Parallel-Meta 3.5 with the GreenGene 138 as reference database to perform the taxonomic classification at different levels (from phylum to species). However, as expected, the performance at the species level is poor. S. epidermidis was not observed from the 16S rRNA data, and in fact about 91% of the reads remain unclassified at the species level (Table R3).*

*Table R3. Taxonomic profiling results of underarm samples at the species level with 16S rRNA sequencing data. We identified 14 species among 20 underarm samples using GreenGenes 138 database. Notably, about 91% of 16S rRNA sequencing reads can’t be assigned with species-level taxonomic annotation.*

*Again, we agree with Reivewer2’s comment (Response #34) that the attempt at a biological interpretation might be distracting and does not add much value to the overall story. Thus, we decided to remove the biological interpretation of underarm profiling results (original Figure 5a, 5b) and focus on the method itself.*

11.	The 266 line supports robustness of the 2bRAD-M method, but the figures suggest otherwise.

*Response 25: The confusion was mainly caused by the narrow range of Y-axis, e.g., L2 similarity ranges from 0.97 to 0.99 in a few panels. Thus, we have now adjusted the figure by unifying the range of Y-axis across different sample types (i.e., Car, FFPE, Home, Underarm) (Figure R9, as shown below). The updated figure below supports the robustness of the 2bRAD-M. Regardless of sample types, all alpha or beta diversity metrics consistently plateau at relatively low sequencing depth.*

*Figure R9 (Figure S6 in the revised manuscript). Rarefaction analysis reveals the desirable sequencing depth of 2bRAD-M for taxonomic profiling of the representative built-environment and FFPE samples. From each of the sample categories (car surfaces, FFPE, home surfaces, and underarm skin), three representative samples were shown. For each sample, we compared key performance metrics of the taxonomic profiling (i.e., alpha diversity, beta diversity, and species-level compositions) at several shallow sequencing depths (by subsampling) with those at deep sequencing depth. The scatter plot in each panel indicates the relationship between a performance metric (Y-axis) and the sequencing depth (X-axis).*

Responses to Reviewer #2

The authors of GBIO-D-21-00210 submission, Sun et al, present a novel method 2bRAD-M for profiling microbial taxonomic composition particularly suitable for samples characterized by low biomass, poor DNA quality (high fragmentation) and highly contaminated with host DNA. The method combines clever experimental and computational design that builds of authors' previous work on enzymatic cleavage of DNA from any organism into predictable fragments suitable for amplification, sequencing and classification at species and higher taxonomic levels. As such, it is a timely a timely addition to the repertoire of reference-based microbiome profiling approaches particularly suited for attractive but hard to work with samples (e.g. biopsies).

While the authors present the intuition behind 2bRAD-M very clearly and I am convinced about the applicability of their approach to the proposed scenarios, they made some questionable choices when evaluating it against mock communities. Also, the presentation of the microbiome profiling in the preserved tissue samples (FFPE) lacks any validation or comparison with other profiling approach (e.g. 16S rRNA), making it impossible to evaluate the 2bRAD-M's performance in these valuable samples. I feel strongly that both these points would need to be addressed so that the manuscript presents a coherent story. These and other comments are elaborated on below.

*Response 26: We thank Reviewer #2 for reviewing our manuscript and giving us positive comments on the applicability, robustness, and potentially broad interest of our approach in the community. We next addressed each of the comments in order.*

Main points:

1.	The choice of Centrifuge for profiling the mock community ATCC MSA1002 with 20 species in Fig. 3b is unfortunate, as you yourself presented in the simulated data in Fig. 2b that Centrifuge is the worst performing among all methods you tested (poor recall, precision and L2 similarity). Rather, please present the results for one of the better performing profilers (e.g. MetaPhlan2), which will give a more accurate representation for precision (and likely L2 similarity).

*Response 27: In the original Figure 2b, we did benchmark with Centrifuge and observed its poor performance using the default settings. It was previously reported that Centrifuge’s poor performance is largely due to the use of default reference database [11]. If the customized database of complete genomes from NCBI RefSeq is used, its performance can be greatly improved and can be as good as state-of-the-art tools such as Bracken or Kraken2 [11]. To avoid any potential confusion and misleading, we decided to remove Centrifuge from the comparison due to its default database issue (Figure R2).*

*In Figure 3a, to validate the 2bRAD-M’s performance and minimize the performance bias introduced by bioinformatic pipelines, we employed Centrifuge to map both WMS (150-bp) and 2bRAD-M (32-bp) generated reads to the reference microbial genomes for sequence abundance estimation for MOCK CAS. Specifically, we replaced the Centrifuge’s default database with a customized database comprising of only genomes of those five species in the MOCK CAS. Moreover, the taxonomic profiling results generated by Centrifuge were not directly used, instead we only employed Centrifuge as a search engine (essentially bowtie2) to estimate the sequence proportion of the five microbial species. Centrifuge was deliberately used in this analysis due to its unique features. To our knowledge, Centrifuge is the only metagenomic profiler that provides alignment results for extremely short reads (as short as ~30 bp), while it can also handle regular Illumina reads. Other main-stream k-mers based classification methods e.g., Kraken2 are not able to generate read-level annotation for 2bRAD fragments.*

*As for the benchmark using MOCK MSA 1002 (original Figure 3b), we agree that using state-of-the-art metagenomic profilers e.g., Bracken, Kraken2, mOTUs2, and MetaPhlAn2 can improve the performance evaluation between 2bRAD-M and WMS. Therefore, we have now supplemented profiling results of MSA 1002 generated by Bracken, Kraken2, mOTUs2, and MetaPhlAn2 (Figure R6). As expected, 2bRAD-M still offers an outstanding performance in decoding MOCK MSA 1002, with its L2 similarity (91.0%), BC similarity (83.1%), precision (90.9%), recall (100%) and Shannon diversity (97.3%) comparable with MetaPlAn2 (who has the best performance among all the WMS profilers used, its L2 similarity, BC similarity, precision, recall and Shannon diversity are 89.1%, 86.7%, 79.2%, 95.0% and 97.5% respectively). We have revised the manuscript accordingly. For additional details, please refer to Response #17 above.*

2.	Why does precision drop so much for 2bRAD-M as the biomass drops (Fig. 3b)? Since you see high recall, it means that you are capturing most of the ATCC MSA1002 species. But drop in precision, unless I interpret it incorrectly, indicates you are picking up unique, species specific markers from "unforeseen microbes". Is it a matter of thresholding on species detection or contamination of the mock community or other? A closer inspection of what you are picking up in the low biomass samples and discussion of the "unforeseen microbes" is important.

*Response 28: High FP rate is a common issue in metagenomic sequencing [11], which will lead to the low precision. Besides the high similarity of some DNA fragments between host genome and microbial genomes, another major reason is the dilemma for end-users to set up an abundance threshold [10]. The balanced point in the tradeoff between precision and recall is the minimum abundance in the sample, which will never be known to end-users. In 2bRAD-M, instead of setting up an abundance threshold, a G-score is used to filter out the presence of FP strains to tackle this challenge. For each species identified within a microbiome sample, its G-score is the geometric mean of the proportion of the species-specific markers that have been captured (by sequencing) and the number of all detected species-specific markers (by sequencing) of this species. We showed that G-score can efficiently evaluate the probability of presence for species (Figure R10). In addition, multi-alignment of conserved reads to different species is one of the major reasons causing FPs, which is naturally avoided in 2bRAD-M since only species-specific markers are used in microbial species identification and abundance estimation (please see Response #5 above).*

*The current 2bRAD-M pipeline only supports the G score as threshold and we used default G score throughout this study to ensure the consistency of our analyses, but ideally, the G score can be automatically generated or adjusted by the sequencing data itself. We think it is the inappropriate G score that has caused the drop of precision in the low biomass MSA 1002 profiling process. To confirm this, we manually adjusted the G score (to 5 and 3) in the pipeline for low biomass MSA 1002 samples and found that the F1 score (precision times recall) increases to 0.9. In fact, we are currently working on an auto-fit G score algorithm using machine learning method, which will be integrated into the next release of 2bRAD-M (v 2.0).*

*Figure R10 (Figure S12 in the revised manuscript). G-score provides a higher precision in taxonomic profiling than the relative abundance based on simulated sequencing data. The identified species is ranked by abundance (by Bracken in the upper plot) or G score (by 2bRAD-M in the lower plot), with color indicating whether it is a false positive (FP). Bracken generates far more FPs than 2bRAD-M, as many FPs are in high abundance for Bracken. In contrast, the G score boundary between true positives and FPs are more prominent when using 2bRAD-M.*

*Accordingly, we have now inserted the following into Methods section (Line 536-541): “To control the false-positive in the species identification, G score was derived for each species identified within a sample as below, which is a geometric mean of the proportion of the species-specific markers that have been captured (by sequencing) and the number of all detected species-specific markers (by sequencing) of this species. G score is more sensitive as the threshold than the relative abundance used in conventional WMS (Fig. S12)”.*

3.	Potentially related, you observe a large inflation of detected species (16-23% more) inflation by 2B-RAD-M in the real stool sample as compared to mOTUs profiling in Fig. 4. How do you explain it? The highly abundant species by 2B-RAD-M but missing from mOTUs, are they present in the mOTUs database?

*Response 29: We agree that it is not fair to compare the profiling results generated by different reference databases, which may lead to problems such as the one here. However, a unified database for benchmarking is not feasible in this study because: (1) 2bRAD-M is a DNA-to-Marker metagenomic profiler (who produce taxonomic abundance as output), thus it is not appropriate to use the sequence abundance generated by DNA-to-DNA metagenomic profilers (e.g., Bracken and Kraken) for benchmarking [9]. (2) The only two DNA-to-Marker metagenomic profilers (e.g., mOTUs2 and MetaPhlAn2) do not support customizing reference database well [11]. Therefore, we fetched 2bRAD tags from WMS data and performed the profiling with real 2b sequencing data using the same 2bRAD-M computational pipeline to rule out the influence from the bioinformatic process (especially the reference database). By comparing the profiling results of WMS-originated and real 2bRAD-M sequencing data, we finally can compare the two data types in a fair manner (Figure R3). In this new benchmarking experiment, a linear correlation was found between WMS data and 2bRAD-M data with an average of 0.99 Pearson coefficient. The alpha diversity (Shannon index) and beta diversity (L2 similarity and BC similarity) of the profiling results are highly similar between these two data types. Thus, we conclude that 2bRAD-M is highly consistent with WMS while only using 1% of the sequencing work. For additional details about this topic, please refer to Response #11 above.*

4.	For the analysis of mock communities, what was the rationale to look at 5 microbe community under varying perturbation setting (Fig. 3a), and not ATCC MSA1002 that contains 20 microbes? Validation of 2bRAD-M on the ATCC MSA1002 in the varying host contamination scenario (95% - 99% host DNA) would be particularly valuable for proposing this method in the setting of terminal ileum or colon biopsies, where we expect a much more complex microbial community and a high host DNA contamination.

*Response 30: In the DNase I digestion method to obtain the degraded DNA samples, a large amount of input DNA (at least 5-10ug) is usually needed, as obtaining the purified degraded DNA by the phenol/chloroform extraction method would result in heavy loss of starting DNA material. However, the concentration of commercially available ATCC MSA1002 DNA sample was very low, ranging from 3 ng/µL to 5 ng/µL, with a total volume of only 55uL in a single purchased tube (https://www.atcc.org/products/msa-1002). Obtaining 30-60 ug (5-10ug * three repeats for two groups) degraded ATCC MSA1002 DNA would require at least 110-360 tubes of starting ATCC MSA1002 DNA which itself costs 268,165 - 877,500 RMB (1 RMB = 0.15 USD). Therefore, we didn’t choose ATCC MSA1002 for simulating the degraded DNA samples in validating 2bRAD-M.*

*On the other hand, we agree that a more complex mock community e.g., MSA 1002 can better demonstrate the robustness of 2bRAD-M in high host DNA contamination. Therefore, we supplemented the 2bRAD sequencing results of 90%, 95%, and 99% host contaminated MSA 1002 mock community in our revised manuscript. As shown in Figure R6 (at the bottom of the bar plots), although the L2 and BC similarity slightly decreased as human DNA contamination increases, 2bRAD-M still outperform 16S rRNA sequencing and the results are highly comparable to WMS sequencing (MetaPhlAn2). This suggested a high sensitivity and repeatability of 2bRAD-M in handling high host-contamination samples. We have revised our manuscript accordingly. Please also refer to Response #17 above.*

5.	For the analysis of formalin fixed, paraffin embedded tissues (FFPE, from line 290 without ground truth and comparison to other profiling approach (e.g. 16S rRNA profiling) this section of the manuscript does not present as a part of a benchmark / validation but rather a biological exploration. As the authors write, sample preparation damages the DNA, introduces cross-linking, samples are of low microbial biomass. Could such conditions be reproduced by FFPE'ing a mock microbial community that is highly contaminated by host DNA? The current validation of a highly degraded DNA presented in Fig. 3a does not seem be a sufficient representation of the FFPE setup. Otherwise, could you benchmark against a set of samples pre- and post-FFPE? The final alternative would be to benchmark 2bRAD-M readout on FFPE samples against a 16S rRNA readout as in the section on environmental and skin samples.

*Response 31: As advised, we have attempted to use both MOCK MSA 1002 and pre- and post-FFPE samples for the validation. However, the experiment with MSA 1002 failed, and this is because the DNA mixture of MSA 1002 is not applicable for the FFPE treatment (which is designed for processing tissue samples instead). Therefore, we now benchmarked against a set of healthy lung tissues from lung adenocarcinoma patients (typically of low microbial biomass) that were derived both before (fresh tissue) and after FFPE. As Figure R11 suggests, although the microbial composition slightly shifted after the FFPE treatment, each paired fresh and FFPE samples from the same tissue maintained a high BC and L2 similarity (over 90.63%) between the pre- and post-FFPE samples. These results thus support the ability of 2bRAD-M to accurately reconstruct microbiome structure from FFPE samples.*

*Figure R11 (Figure S7 in the revised manuscript). Comparing the taxonomic profiling results of 2bRAD-M between fresh (i.e., pre-FFPE) and post-FFPE lung tissues. Three pairs of healthy lung tissues from lung adenocarcinoma patients, both before and after FFPE processing, were sequenced via 2bRAD-M. Bar plots illustrate their microbial composition. The bars on the same row indicate L2 similarity and BC similarity between each pair of fresh and FFPE samples.*

*Accordingly, we have now inserted the following into Results (Line 296-301): “To test whether 2bRAD-M can tackle FFPE samples, we started with three pairs of healthy lung tissues from lung adenocarcinoma patients (typical of low microbial biomass), each derived both before (i.e., fresh tissue) and after FFPE (Methods). For each of the three pairs of lung tissue samples, a high consistency of microbial profiles derived using 2bRAD-M was reported between pre- and post-FFPE samples (Fig. S7). These results thus support the ability of 2bRAD-M to accurately reconstruct microbiome structure from FFPE samples.”*

*We have also inserted below into Methods (Line 401-403): “Three pairs of fresh and FFPE samples from healthy lung tissues of lung adenocarcinoma patients, and 45 cervical cancer-related sections in FFPE blocks (with a thickness of 4μm and an area of 3cm2), underwent microbiome profiling via 2bRAD-M.”*

Minor:

1.	Please, provide a more detailed method section on the validation sections, i.e. how simulated or sequenced samples are mapped (e.g. alignment, classification, what method).

*Response 32: As advised, we have now expanded the Methods section to detail how simulated or sequenced samples were mapped using the various taxonomic profilers. Briefly, to compare the performance between 2bRAD-M and current state-of-the-art metagenomic profilers, we used default database and parameters for Kraken2 (version: v2.0.8-beta, database version: RefSeq, 2019 Oct), Bracken (version: Bracken v2.5, database version: RefSeq, 2019 Oct), MetaPhlAn2 (version: 2.96.1, database version: mpa v296 CHOCOPhlAn 201901), and mOTUs2 (version: 2.5.1, database version: 2.5.0), which are in line with those used in Ye et al. Cell paper [11]. For details, please see Response #7 above.*

2.	The choice of mapping against database of species unique tags as opposed to all tags or complete genomes is hard to question. But it would be interesting to see the increasing benefit (recall, precision, L2) of this approach as compared to all tags and complete genomes on simulated data or 5 / 20 species mock communities.

*Response 33: As advised, to illustrate the benefit of using unique 2bRAD tags as a reference database, we have compared the performance of using different reference databases to decode taxonomic composition in MOCK MSA 1002. Specifically, all microbial genomes and all theoretically existent 2bRAD tags were separately used for reference database construction (species level) and replaced the unique 2b tag database for taxonomy classification. Then, profiling results of 2bRAD-M sequencing of MOCK MSA 1002 by the three approaches (unique 2b tag database, all 2bRAD tags, and all genomes as reference) were compared. As expected, no difference was found between using all 2bRAD tags and all microbial genomes as the reference database (since 2bRAD reads will only be mapped to the restriction enzyme site in the genomes), yet their performance was lower than using unique 2bRAD tags as the reference (Figure R12). Specifically, although the recall is similar among the three approaches, the precision and L2 are much improved while using unique 2bRAD tags as reference database. Therefore, the 2b unique reference database has great potential in solving false-positive issue in microbial identification and in increasing the accuracy of abundance estimation.*

*Figure R12 (Figure S11 in the revised manuscript). Comparison of MSA 1002 profiling results using different databases. Left panel: the ground truth of MSA 1002. Middle panel: profiling results by the standard 2bRAD-M pipeline (using unique 2bRAD tags as reference database). Right panel: profiling results by using all 2bRAD tags or all microbial genomes (as reference databases).*

*Accordingly, we have now inserted the following into Results section (Line 488-489): “The species-specific marker database has been shown to outperform other reference databases using all 2bRAD tags or full genomes in taxonomic profiling (Fig. S11).”*

3.	Lines 283-288 and 318-327. I think the strength of the manuscript is the method, its validation and applicability in various difficult settings. The attempt at a biological interpretation is distracting, does not add much value to the overall story and could be taken out.

*Response 34: We agree that the attempt at a biological interpretation might be distracting. So, we decided to remove some biological interpretations e.g., explanations of underarm profiling results (original Figure 5a, 5b and relevant text).*

4.	In panel 4d, please rename y-axis from shotgun to WMS (or choose other consistent naming scheme).

*Response 35: As advised, we have now changed Y-axis label and the figure legends from “shotgun” to “WMS” (please see Figure R3 above) and kept this terminology consistent throughout the whole paper.*

*Reference cited in this response letter:*

*1.	Ferretti, P., et al., Experimental metagenomics and ribosomal profiling of the human skin microbiome. Exp Dermatol, 2017. 26(3): p. 211-219.*

*2.	Horz, H.P., et al., Selective isolation of bacterial DNA from human clinical specimens. J Microbiol Methods, 2008. 72(1): p. 98-102.*

*3.	Feehery, G.R., et al., A method for selectively enriching microbial DNA from contaminating vertebrate host DNA. PLoS One, 2013. 8(10): p. e76096.*

*4.	Gu, W., et al., Depletion of Abundant Sequences by Hybridization (DASH): using Cas9 to remove unwanted high-abundance species in sequencing libraries and molecular counting applications. Genome Biol, 2016. 17: p. 41.*

*5.	Ganda, E., et al., DNA Extraction and Host Depletion Methods Significantly Impact and Potentially Bias Bacterial Detection in a Biological Fluid. mSystems, 2021. 6(3): p. e0061921.*

*6.	Qin, J., et al., A human gut microbial gene catalogue established by metagenomic sequencing. Nature, 2010. 464(7285): p. 59-65.*

*7.	Huang, Y.F., et al., Analysis of microbial sequences in plasma cell-free DNA for early-onset breast cancer patients and healthy females. BMC Med Genomics, 2018. 11(Suppl 1): p. 16.*

*8.	Hillmann, B., et al., Evaluating the Information Content of Shallow Shotgun Metagenomics. mSystems, 2018. 3(6).*

*9.	Sun, Z., et al., Challenges in benchmarking metagenomic profilers. Nat Methods, 2021. 18(6): p. 618-626.*

*10.	Breitwieser, F.P., J. Lu, and S.L. Salzberg, A review of methods and databases for metagenomic classification and assembly. Brief Bioinform, 2019. 20(4): p. 1125-1136.*

*11.	Ye, S.H., et al., Benchmarking Metagenomics Tools for Taxonomic Classification. Cell, 2019. 178(4): p. 779-794.*

*12.	Meyer, F., et al., Critical Assessment of Metagenome Interpretation - the second round of challenges. bioRxiv, 2021: p. 2021.07.12.451567.*

*13.	Fritz, A., et al., CAMISIM: simulating metagenomes and microbial communities. Microbiome, 2019. 7(1): p. 17.*

*14.	Parks, D.H., et al., A complete domain-to-species taxonomy for Bacteria and Archaea. Nat Biotechnol, 2020. 38(9): p. 1079-1086.*

*15.	Milanese, A., et al., Microbial abundance, activity and population genomic profiling with mOTUs2. Nat Commun, 2019. 10(1): p. 1014.*

*16.	Truong, D.T., et al., MetaPhlAn2 for enhanced metagenomic taxonomic profiling. Nat Methods, 2015. 12(10): p. 902-3.*

*17.	Beghini, F., et al., Integrating taxonomic, functional, and strain-level profiling of diverse microbial communities with bioBakery 3. Elife, 2021. 10.*

**Second round of review**

**Reviewer 1**

In their revised manuscript, the authors have nicely addressed most of my concerns.

A few final minor requests would be necessary, to enable interpretability by the scientific community:

1. Table R1(S3) provides an impressive input, yet it is not based on “real-life” data. Could you please add this funnel to the “real-life” samples you have sequenced?

2. The authors should provide a 2bRAD-M database on top of the GTDB reference and make it available for the entire scientific community, as it is the most comprehensive and is supported by the results in the PBP. I believe this will be a valuable asset to promote the usage of the authors’ method.

3. Figure S1b – I believe it is meant to be a log2 scale

**Reviewer 2**

Thank you for your efforts in revising the manuscript. All my concerns have been addressed satisfactory.

**Authors Response**

**Point-by-point responses to the reviewers’ comments:**

Responses to Reviewer #1

In their revised manuscript, the authors have nicely addressed most of my concerns. A few final minor requests would be necessary, to enable interpretability by the scientific community:

1. Table R1(S3) provides an impressive input, yet it is not based on “real-life” data. Could you please add this funnel to the “real-life” samples you have sequenced?

*We thank Reviewer #1 for the positive assessment on our revision. Now Table S3 has been updated with “real-life” samples (FFPE samples) included. Please see our Additional file 1: Table S3.*

2. The authors should provide a 2bRAD-M database on top of the GTDB reference and make it available for the entire scientific community, as it is the most comprehensive and is supported by the results in the PBP. I believe this will be a valuable asset to promote the usage of the authors’ method.

*As advised, we have released a new reference database based on the GTDB. Now the users can choose to use RefSeq or GTDB for profiling by setting up the parameter “-d <dir>”. Please see our Github repo (https://github.com/shihuang047/2bRAD-M) for more details.*

3. Figure S1b – I believe it is meant to be a log2 scale

*Sorry for the typo, we accidentally performed ln transformation of Y-axis for Figure S1a and S1b. Now we have revised the figures using a log-10 transformation of Y-axis in our new manuscript (please see Additional file1: Fig.S1).*

Responses to Reviewer #2

Thank you for your efforts in revising the manuscript. All my concerns have been addressed satisfactory.

*We thank Reviewer #2 for investing time to review this manuscript, we really appreciate all her/his comments and suggestions.*
